# Whey Protein Supplementation with or without Vitamin D on Sarcopenia-Related Measures: A Systematic Review and Meta-Analysis

**DOI:** 10.1016/j.advnut.2023.05.011

**Published:** 2023-05-15

**Authors:** Nasrin Nasimi, Zahra Sohrabi, Everson A. Nunes, Erfan Sadeghi, Sanaz Jamshidi, Zohreh Gholami, Marzieh Akbarzadeh, Shiva Faghih, Masoumeh Akhlaghi, Stuart M. Phillips

**Affiliations:** 1 Department of Community Nutrition, School of Nutrition and Food Sciences, Shiraz University of Medical Sciences, Shiraz, Iran; 2 Nutrition Research Center, School of Nutrition and Food Sciences, Shiraz University of Medical Sciences, Shiraz, Iran; 3 Exercise Metabolism Research Group, Department of Kinesiology, McMaster University, Hamilton, Ontario, Canada; 4 Laboratory of Investigation of Chronic Diseases, Department of Physiological Sciences, Federal University of Santa Catarina, Florianópolis, Brazil; 5 Research Consultation Center (RCC), Shiraz University of Medical Sciences, Shiraz, Iran; 6 Department of Nutrition, School of Public Health, Iran University of Medical Sciences, Tehran, Iran

**Keywords:** resistance exercise, frailty, muscle, mobility, physical function

## Abstract

The effects of supplementation with whey protein alone or with vitamin D on sarcopenia-related outcomes in older adults are unclear. We aimed to assess the effect of whey protein supplementation alone or with vitamin D on lean mass (LM), strength, and function in older adults with or without sarcopenia or frailty. We searched PubMed, Web of Science, and SCOPUS databases. Randomized controlled trials (RCT) that investigated the effect of whey protein supplementation with or without vitamin D on sarcopenia outcomes in healthy and sarcopenic or frail older adults were included. Standardized mean differences (SMDs) were calculated for LM, muscle strength, and physical function data. The analysis showed that whey protein supplementation had no effect on LM and muscle strength; nevertheless, a significant improvement was found in physical function (SMD = 0.561; 95% confidence interval [CIs]: 0.256, 0.865, *n* = 33), particularly gait speed (GS). On the contrary, whey protein supplementation significantly improved LM (SMD = 0.982; 95% CI: 0.228, 1.736; *n* = 11), appendicular lean mass and physical function (SMD = 1.211; 95% CI: 0.588, 1.834; *n* = 16), and GS in sarcopenic/frail older adults. By contrast, co-supplementation with vitamin D enhanced LM gains (SMD =0.993; 95% CI: 0.112, 1.874; *n* = 11), muscle strength (SMD =2.005; 95% CI: 0.975, 3.035; *n* = 11), and physical function (SMD = 3.038; 95% CI: 2.196, 3.879; *n* = 18) significantly. Muscle strength and physical function improvements after whey protein supplementation plus vitamin D were observed without resistance exercise (RE) and short study duration subgroups. Moreover, the combination of whey protein and vitamin D with RE did not enhance the effect of RE. Whey protein supplementation improved LM and function in sarcopenic/frail older adults but had no positive effect in healthy older persons. By contrast, our meta-analysis showed that co-supplementation with whey protein and vitamin D is effective, particularly in healthy older adults, which is likely owing, we propose, to the correction of vitamin D insufficiency or deficiency.

The trial was registered at https://inplasy.com as INPLASY202240167.

**Statements of Significance**

This meta-analysis examined the effect of whey protein supplementation with or without vitamin D on lean mass and sarcopenia-related outcomes. We report that whey and vitamin D supplementation could augment lean mass and sarcopenia-related outcomes in certain populations and some circumstances. These are novel findings because whey protein and vitamin D are frequently included ingredients in oral nutritional supplements targeted at older persons requiring skeletal muscle function.

## Introduction

Sarcopenia is a geriatric syndrome characterized by the progressive loss of skeletal muscle mass, strength, and physical function. It is associated with falls, fractures, disability, frailty, and an increased risk of chronic metabolic diseases. People classified as sarcopenic are more frequent users of health care systems [[1], [2], [3]]. Guidelines for sarcopenia treatment [4] include incorporating resistance exercise (RE), optimizing protein intake, and addressing insufficiency or deficiency of vitamin D; however, there is a lack of clarity regarding the effective combination of these variables. Higher habitual protein intakes are associated with lean mass (LM) preservation and improved muscle function in older adults [5,6]. A recent meta-analysis showed that protein intakes of 1.2–1.59 g/kg/d added to RE training positively affected LBM and function in older adults [7]. Whey protein is an easily digestible high-quality protein containing all essential amino acids, particularly leucine, a key activator of muscle protein synthesis [8].

Vitamin D has multiple effects on the muscle, such as regulation of gene expression, differentiation, neuromuscular function, and production of anti-inflammatory cytokines [9,10]. Studies have also shown that optimizing vitamin D status is important in maintaining muscle mass and function in older adults [11], and vitamin D status is related to muscle mass and strength changes [10]. Notably, the normalization of circulating vitamin D concentration from deficient or insufficient to replete may be critical in dictating the efficacy of protein (or other amino acids) supplementation [12,13]. Although some interventions, such as whey protein supplementation and vitamin D optimization, are suggested as effective ways to protect against sarcopenia, the efficacy of such interventions is unclear.

Although several systematic reviews on different populations and interventions have been published in recent years [[14], [15], [16], [17]], a conclusion regarding whether whey protein supplementation alone or with vitamin D is beneficial for sarcopenia-related outcomes in older people is unknown. This systematic review and meta-analysis aimed to comprehensively evaluate the effects of whey protein supplementation with or without vitamin D and with or without RE on LM, muscle strength, and physical function in older adults with or without sarcopenia or frailty.

## Methods

This systematic review and meta-analysis was developed according to the guidelines laid out in the Cochrane Handbook for Systematic Reviews of Interventions [18] and the Preferred Reporting Items for Systematic Reviews and Meta-Analyses (PRISMA) report [19]. The study protocol was registered in the International Platform of Registered Systematic Review and Meta-analysis Protocols (INPLASY; registration number: INPLASY202240167). All research team members agreed on the study’s steps: identification, screening, data extraction, and analysis.

### Eligibility criteria

Inclusion/exclusion criteria based on the PICOS (population, intervention, comparison, outcome, and setting) of the study are detailed in Table 1. To be considered eligible for inclusion, studies were required to be an RCT investigating the effects of whey protein supplementation or whey protein and vitamin D co-supplementation (with or without exercise training) on measures of sarcopenia in older adults with or without sarcopenia or frailty (older than 60 years) and without any other overt disease that may affect the outcome. Randomized controlled trials (parallel or crossover design) published in English were included. Nonhuman studies (animal, in vitro, and in vivo studies), cross-sectional studies, reviews, gray literature (book chapters, abstracts in conferences, editorials, letters, and seminars), studies without any control groups or with protein-based control groups, studies with a special diet, and studies lacking information for extracting mean and standard deviation [SD; or standard error (SE)] were excluded. No restriction was considered on the type of whey protein and its dosage, intervention duration, measurement tools, and type or duration of RE. It should be noted that although we did not limit the type and duration of RE, only those RCTs were included in which both intervention and control groups were exposed to the same RE.

**TABLE 1:** PICOS statement that guided the systematic review

Parameter	Inclusion criteria	Exclusion criteria
Population	Older adults with or without sarcopenia or frailty (older than 60 years) and without any other overt disease that may affect the outcome	Participants with medical conditions affecting outcome measures, such as cancer, diabetes, chronic obstructive pulmonary disease, and neurological disorders (e.g., Parkinson or dementia)
Intervention	Whey protein supplementation (with or without exercise training), and whey protein and vitamin D co-supplementation (with or without exercise training)	Interventions with special diets such as the ketogenic diet or co-supplementation with other nutrients or active ingredients with possible effects on LBM (such as omega-3, vitamin E, creatine, and casein)
Comparator	No intervention (control) or isocaloric and nonisocaloric placebo (carbohydrate based)	No control group or protein-based placebo (such as soy or milk protein)
Outcome	Sarcopenia-relevant measures, such as lean mass or appendicular lean mass, OR muscle strength (upper body strength: handgrip or lower body strength: leg press or knee extension), OR physical function [gait speed (m/s)], SPPB score, or TUG, time stand, chair stand, balance test, sit-to-stand, and walking tests	Not reporting any of the outcome measures
Study design	Randomized control trial (RCT)	Not an RCT

Abbreviations: SPPB, Short Physical Performance Battery; TUG, timed up-and-go.

### Systematic search strategy

A comprehensive literature search was performed for RCT investigating the effects of whey protein supplementation on sarcopenia measures up to June 2022. Electronic databases, such as PubMed, Web of Science, and SCOPUS, were searched to find eligible articles. The search strategy was mentioned in the study protocol (available on https://inplasy.com/inplasy-2022-4-0167/) and the supplementary file “search strategy.” References were imported to the software Endnote X8 for removing duplicates and screening.

### Screening, data extraction, and outcome measures

Studies were screened for eligibility criteria, such as study design, whey protein supplementation intervention, participant’s condition, control group, and measures of sarcopenia, such as LBM, strength, or physical function. The titles and abstracts were reviewed in duplicate by members of the review team (NN, ZG), and any, if marked for inclusion by either reviewer, were reviewed as a full text. Two people conducted full-text inclusion independently (NN, ZG). All disagreements were resolved through discussions. One investigator (NN) extracted data, which were checked for accuracy by a second investigator (SJ). Data on study characteristics, population details, intervention and control conditions, and numerical data for the outcomes of LM [total lean mass (TLM) and appendicular lean mass (ALM)], muscle strength (upper body and lower body strength), and physical function were extracted. Muscle strength outcomes included upper body strength [handgrip strength (HGS)] or lower body strength (leg press or knee extension). Physical function outcomes were as follows: gait speed (GS; in meters per second), Short Physical Performance Battery (SPPB) score, or other physical tests with units of seconds, such as time up-and-go, time chair stand balance test, sit-to-stand, and walking time. Because different studies used multiple tests to measure such outcomes, we used all information reported for muscle mass, strength, and physical function outcomes. The Get Data Graph Digitizer (http://getdata-graph-digitizer.com/) was used to extract data from the figures when the data were not available in the tables, text, or after multiple attempts to contact authors.

### Quality assessment and risk of bias

The Grading of Recommendation Assessment, Development, and Evaluation (GRADE) system was used to evaluate the quality of evidence for the study outcomes. Six criteria were considered: risk of bias, inconsistency, indirectness, imprecision, publication bias, and effect size. A GRADE rating summary was generated using the GRADEpro platform (https://gdt.gradepro.org/). The risk of bias was assessed using a Revised Cochrane Risk of Bias tool for randomized trials (RoB2) and ROBVIS-1 tool [20]. All included studies were evaluated for sources of bias in selection, performance, detection, attrition, and selective reporting. On the basis of the guidelines, studies were classified into low-risk, with some concerns, and high-risk categories. All risk-of-bias assessments were performed in duplicate (NN, MA).

### Statistical analysis

The mean difference (MD) or standardized mean difference (SMD) of change as the effect size with their corresponding SEs were calculated from the primary studies. The 3-level random-effects model was used to estimate the pooled effect size and the 95% confidence intervals (CIs). The SD of change was calculated using the following formula:

$$SDchange=\sqrt{{SD}_{Pre}^{2}+{SD}_{Post}^{2}-\left({SD}_{Pre}\times {SD}_{Post}\right)}$$

Similar to the previous meta-analysis [7], for pooling of physical function outcomes, the direction of effect was adjusted to ensure consistency of the outcome responses, that is, a reduction in GS measured in seconds to perform the task reflects a better outcome. By contrast, increasing GS measured in meter per second reflects a positive outcome. Similarly, a reduction in sit-to-stand test (seconds), 5-chair repetition test (seconds), and timed up-and-go test (in seconds) are considered positive responses, whereas an increase in 1-leg standing test (seconds) and SPPB score reflects a positive outcome. Therefore, when a decrement in the absolute variable was considered the positive or beneficial outcome, we multiplied the δ values by −1. This approach normalizes all effect sizes, meaning all SMDs are positive and reflect improvements. All data were extracted and included in the analysis for studies that evaluated sarcopenia outcomes using various methods. Statistical heterogeneity was examined using Cochran Q test and *I*^2^ statistic. We interpreted *I*^2^ of 30% to 60% as moderate and 60% to 90% as substantial heterogeneity. A subgroup analysis was conducted to determine the sources of heterogeneity according to the measurements, RE (with and without), the dose of supplementation (≤20 g and >20 g), and intervention duration (≤12 weeks and >12 weeks). A sensitivity analysis based on the leave-1-out method was conducted to investigate whether the results were robust. Begg rank correlation test, Egger linear regression test, and funnel plots were used to detect publication bias. All analyses were performed using the *metafor* package in R v.4.2 (R Foundation for Statistical Computing, Vienna, Austria; http://www.R-project.org/).

## Results

### Literature search and study selection

**FIGURE 1:**
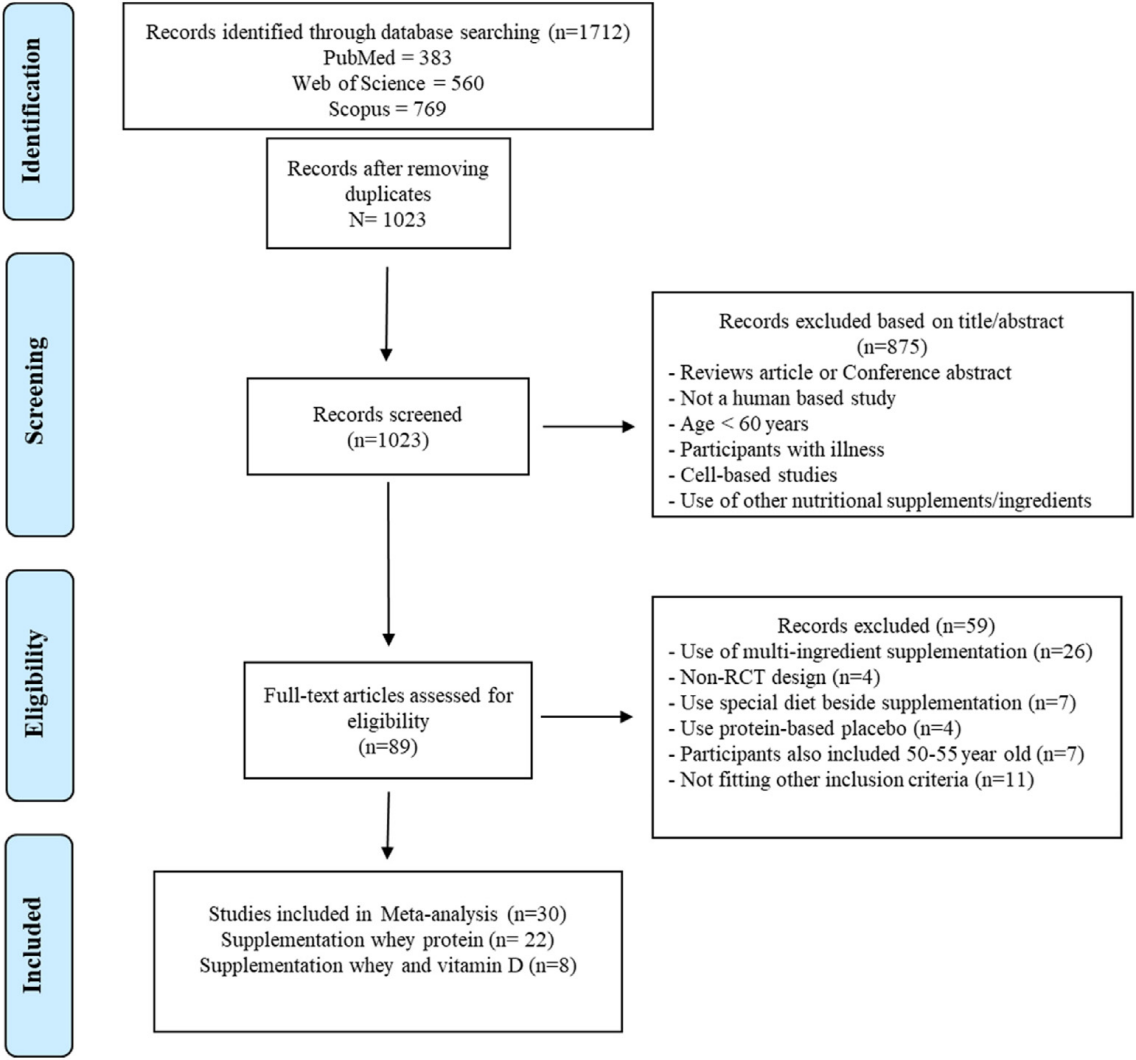
Preferred Reporting Items for Systematic Reviews and Meta-Analysis (PRISMA) flow chart of the study selection process.

The results of the literature search are shown in Figure 1. Three databases were searched, and 1712 records were identified. After screening for duplicate and nonrelevant studies, 89 articles were selected for full-text reviewing and eligibility. Accordingly, 59 studies were excluded owing to the following reasons: using multi-ingredient supplementation (i.e., whey protein and vitamin D, other components such as vitamin E, and omega-3 lipids), applying particular diet therapy besides the supplementation, studying postmenopausal women in the age range of 50–55 years, and using protein-based placebo in the control group. Finally, 30 RCTs remained at the end of the screening process, including 22 studies evaluating whey protein supplementation alone and 8 studies assessing whey protein plus vitamin D supplementation. A meta-analysis was performed separately on each group of studies.

### Study characteristics for RCTs

The summary information from the included studies is presented in Supplemental Table S1. The studies were performed in Brazil [[21], [22], [23], [24], [25]], United States [[26], [27], [28], [29]], United Kingdom [30,31], Europe [11,[32], [33], [34], [35], [36], [37], [38], [39], [40], [41], [42]], Canada [43], Australia [44], China [45], Korea [46], Japan [47,48], and Taiwan [49]. These studies were published between 2012 and 2022. The participants in the studies were healthy [21,23,25,27,[30], [31], [32], [33],^36^,^37^,^39^,[42], [43], [44],^47^,^48^], sarcopenic [11,24,35,40,41,45,49], frail [22,38,46], and mobility-limited [26,28,29,34] older adults (aged 60 years or older). The intervention duration ranged from 12 weeks to 2 years and 8 weeks to 24 weeks in whey protein and combined whey protein and vitamin D studies, respectively. The dose of whey protein varied from 15 to 40 g/d (68% of the studies prescribed >20 g/d) in the studies evaluating whey protein supplementation and from 8.5 to 40 g/d (38% of the studies specified>20 g/d) in studies related to the whey protein plus vitamin D supplementation. Moreover, the dose of vitamin D ranged from 100 to 800 IU. The placebo or control groups received carbohydrate-based nutrients such as maltodextrin, corn starch, collagen, or nothing. However, studies differed on isocaloric or non-isocaloric placebo in the control groups. Furthermore, 20 studies (66% of all included RCTs) had RE in both intervention and control groups [[21], [22], [23], [24], [25],[28], [29], [30], [31],[34], [35], [36], [37], [38],[40], [41], [42], [43],^47^,^48^], including 15 of 22 trials of whey protein supplementation and 5 of 8 trials of whey and vitamin D co-supplementation.

Almost all included studies (28/30) evaluated LM changes regarding TLM [21,22,24,[26], [27], [28], [29], [30],^33^,^34^,[36], [37], [38],^40^,^43^,^45^,^49^] and ALM [11,[21], [22], [23], [24], [25],[29], [30], [31], [32],^36^,^37^,^39^,^41^,^42^,[44], [45], [46],^48^,^49^]. Data regarding the effect of whey protein supplementation alone or with vitamin D on muscle strength were extracted from 17 studies for HGS [11,22,31,32,35,[38], [39], [40], [41], [42], [43], [44], [45], [46], [47], [48], [49]] and 20 studies for lower body strength, such as knee extension [[21], [22], [23], [24], [25],[28], [29], [30], [31], [32], [33], [34],[36], [37], [38],^43^,^44^,^47^,^48^] and leg press [11,22,31,32,35,[38], [39], [40], [41], [42],[44], [45], [46], [47], [48], [49]]. Changes in physical function were extracted from 22 studies, including SPPB [11,[28], [29], [30], [31], [32],^35^,^36^,^39^,^45^,^46^], GS (in meters per second) [11,[28], [29], [30],^32^,^34^,^42^,^43^,[45], [46], [47],^49^], and other physical tests (seconds) [11,22,24,28,32,33,36,37,39,[42], [43], [44], [45], [46],^48^]. Fat mass was also reported in 14 studies [21,24,[26], [27], [28], [29], [30], [31], [32],^36^,^40^,^42^,^43^,^49^].

## Meta-analysis

### Effect of whey protein supplementation on LM

**FIGURE 2:**
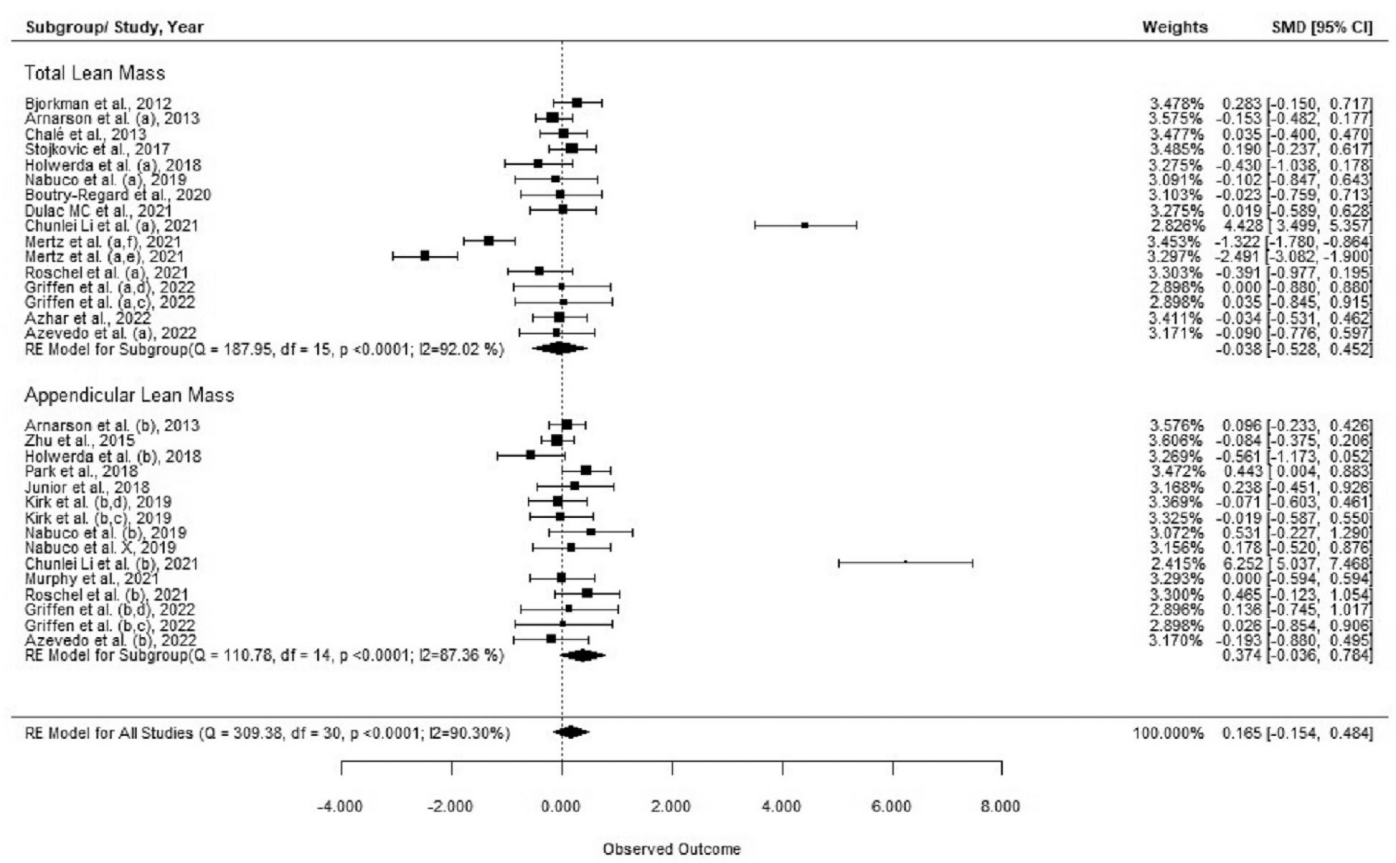
Forest plot of the randomized clinical trials (RCTs) examining the effect of whey protein supplementation on lean mass (LM; subgroups for total and appendicular lean mass). Data are expressed as standardized mean differences (SMDs) between the intervention and control groups with 95% CI. Estimates were pooled using the random-effects Hedges model. Letters between parentheses represent the following: a, TLM; b, ALM; c, with exercise training; and d, without exercise training. Abbreviations: ALM, appendicular lean mass; CI, confidence interval; SMD, standard mean difference; TLM, total lean mass.

All included studies showed that whey protein supplementation had no significant effect on LM (SMD = 0.165; 95% CI: −0.154, 0.484; *n* = 31) (Table 2; Figure 2 and Supplemental Figure S2). On the contrary, the subgroup analysis showed that whey protein supplementation significantly improved LM in sarcopenic/frail older adults (SMD = 0.982; 95% CI: 0.228, 1.736; *n* = 11) (Table 2; Supplemental Figure S3). The results from other subgroup analyses, including TLM/ALM, RE, whey dose, and study duration, showed no significant effect. Analyses also showed that the changes in LM were mostly related to changes in ALM compared with those to TLM (SMD = 0.374; 95% CI: −0.036, 0.784; *n* = 15 vs. SMD = −0.038; 95% CI: −0.528, 0.452; *n* = 16, respectively) (Supplemental Table S1), although this was not statistically significant.

**TABLE 2:** Effect of whey protein supplementation on changes in LM

Groups/subgroups	Effect size (SMD)	95% CI	No. of interventions/outcomes	*I*^2^ (%)	*P*-heterogeneity	*P* between-subgroup heterogeneity
All RCTs	0.165	−0.154, 0.484	31	90.30	<0.001	
RCT conducted on healthy older adults	-0.238	−0.511, 0.034	20	80.04	<0.001	0.321
RCT conducted on sarcopenic/frail older adultsis	0.982	0.228, 1.736	11	94.36	<0.001	
RCT measuring TLM	-0.038	−0.528, 0.452	16	92.02	<0.001	0.206
RCT measuring ALM	0.374	−0.036, 0.784	15	87.36	<0.001	
RCT with RE	0.165	−0.154, 0.484	19	0.00	0.650	0.193
RCT without RE	0.536	−0.266, 1.338	12	96.26	<0.001	
RCT with high dose of whey (>20 g/d)	0.001	−0.126, 0.127	21	0.00	0.671	0.174
RCT with low dose of whey (<20 g/d)	0.619	−0.265, 1.502	10	96.92	<0.001	
RCT with high duration (>12 wk)	0.463	−0.240, 1.167	13	95.94	<0.001	0.210
RCT with low duration (≤12 wk)	0.005	−0.131, 0.140	18	0.00	0.712	

Abbreviations: ALM, appendicular lean mass; RCT, randomized controlled trial; RE, resistance exercise; SMD, standardized mean difference; TLM, total lean mass.

The separate analysis of TLM and ALM are also summarized in Supplemental Tables S4 and S5, respectively. The results indicated that whey protein supplementation did not significantly affect TLM (MD = −0.069; 95% CI: −0.499, 0.362; *n* = 16) (Supplemental Figure S4) or ALM (MD = 0.166; 95% CI: −0.093, 0.426; *n* = 15 (Supplemental Figure S5). However, the analysis showed a significant increase in ALM in the sarcopenic or frail older adult subgroup (MD = 0.564; 95% CI: 0.520, 0.609; *n* = 4) (Supplemental Table S5).

### Effect of whey protein supplementation on muscle strength

The overall effects of whey protein supplementation on muscle strength are presented in Table 3 and Supplemental Figure S6. The analysis revealed no change in strength with whey protein supplementation subgrouping sarcopenic or frail and healthy older adults (Supplemental Figure S7) and lower and upper strength (Supplemental Figure S8). However, when performing the analysis in the subgroup with RE, the positive effect of whey protein supplementation on strength was detected in the subgroup in which both intervention and control groups were exposed to RE (SMD = 0.238; 95% CI: 0.001, 0.474; *n* = 21) (Supplemental Figure S9) compared with that in the subgroup without RE. Moreover, whey protein at doses higher than 20 g improved muscle strength (SMD = 0.252; 95% CI: 0.051, 0.453; *n* = 25) (Supplemental Figure S10). The analysis of HGS showed that whey protein supplementation had no significant effect (MD = 0.534; 95% CI: −0.742, 1.810; *n* = 11) (Supplemental Table S6; Supplemental Figure S11). In addition, after analyzing the effects on lower body strength, whey protein supplementation was ineffective in increasing strength (MD = 1.187; 95% CI: −0.861, 3.235; *n* = 21) (Supplemental Table S7; Supplemental Figure S12).

**TABLE 3:** Effect of whey protein supplementation on changes in muscle strength

Groups/subgroups	Effect size (SMD)	95% CI	No. of interventions/outcomes	*I*^2^ (%)	*P*-heterogeneity	*P* between-subgroup heterogeneity
All RCTs	0.149	−0.086, 0.383	32	83.81	0.214	
RCT conducted on healthy older adults	0.178	**−**0.097, 0.454	21	82.29	<0.001	0.250
RCT conducted on sarcopenic/frail older adults	0.085	**−**0.375, 0.546	11	87.13	<0.001	
RCT measuring upper body strength	0.079	**−**0.336, 0.494	11	86.97	<0.001	0.671
RCT measuring lower body strength	0.189	**−**0.104, 0.482	21	82.48	<0.001	
RCT with RE	0.238	0.001, 0.474	21	70.99	<0.001	0.296
RCT without RE	**−**0.052	**−**0.540, 0.436	11	91.56	<0.001	
RCT with high dose of whey (>20 g/d)	0.252	0.051, 0.453	25	69.66	<0.001	0.141
RCT with low dose of whey (<20 g/d)	**−**0.349	**−**1.123, 0.426	7	94.12	<0.001	
RCT with high duration (>12 wk)	0.079	-0.230, 0.388	20	87.39	<0.001	0.472
RCT with low duration (≤12 wk)	0.253	-0.105, 0.610	12	72.97	<0.001	

Abbreviations: RCT, randomized controlled trial; RE, resistance exercise; SMD, standardized mean difference.

### Effect of whey protein supplementation on physical function

**FIGURE 3:**
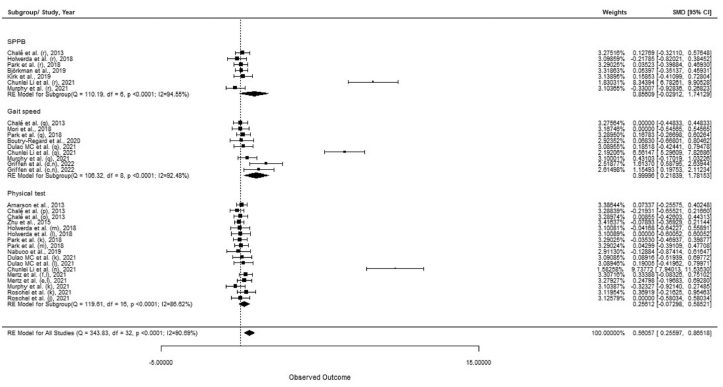
Forest plot of the randomized clinical trials (RCTs) examining the effect of whey protein supplementation on physical function [subgrouping SPPB (score), gait speed (GS; m/s), and other physical tests (s)]. Data are expressed as SMDs between the intervention and control groups with 95% CI. Estimates were pooled using the random-effects Hedges model. Letters between parentheses represent the following: c, with exercise training; d, without exercise training; e, control (CHO); f, control (collagen), j, timed stand; k, TUG; l, walking time (s); m, sit-to-stand; n, chair stand; o, chair rise; p, stair climb; q, GS; and r, SPPB. Abbreviations: CI, confidence interval; GS, gait speed; SMD, standard mean difference; SPPB, Short Physical Performance Battery; TUG, timed up-and-go.

The analysis revealed that whey protein supplementation improved physical performance (SMD = 0.561; 95% CI: 0.256, 0.865; *n* = 33) (Table 4; Figure 3 and Supplemental Figure S13). Moreover, significant effects were found when considering sarcopenic or frail participants (SMD = 1.211; 95% CI: 0.588, 1.834; *n* = 16) (Supplemental Figure S14), GS (SMD = 1.00; 95% CI: 0.218, 1.782; *n* = 9), without RE (SMD = 1.551; 95% CI: 0.834, 2.267; *n* = 14) (Supplemental Figure S15), dose lower than 20 g of whey protein supplement (SMD = 3.379; 95% CI: 1.765, 4.994; *n* = 7) (Supplemental Figure S16), and study duration of >12 weeks (SMD = 1.042; 95% CI: 0.503, 1.582; *n* = 18) (Supplemental Figure S17). Effects of whey protein supplementation on SBBP, GS, and other physical tests are presented in Supplemental Tables S8–S10. The analysis considering all RCTs showed no significant main effects in SPPB (MD = 0.186; 95% CI: −0.507, 0.879; *n* = 7) (Supplemental Figure S18) and other physical tests (MD = 0.209; 95% CI: −0.434, 0.853; *n* = 17) (Supplemental Figure S20). There was no effect of whey protein supplementation on GS (MD = 0.061; 95% CI: −0.001, 0.122; *n* = 9) (Supplemental Table S9; Supplemental Figure S19). However, a subgroup analysis by RE and >20 g doses of whey protein showed that GS was higher in participants supplemented with whey protein (MD = 0.034; 95% CI: 0.004, 0.064; *n* = 5, and MD = 0.047; 95% CI: 0.018, 0.076; *n* = 4, respectively). Furthermore, a significant effect was found for subgroups of sarcopenic or frail participants (MD = 0.051; 95% CI: 0.016, 0.086; *n* = 5) and study duration ≤12 weeks (MD = 0.064; 95% CI: 0.034, 0.095; *n* = 5). In addition, results showed that supplementation with whey had no significant effect on fat mass (MD = −0.033; 95% CI: −0.465, 0.398; *n* = 12) (Supplemental Table S11).

**TABLE 4:** Effect of whey protein supplementation on changes in physical function

Groups/subgroups	Effect size (SMD)	95% CI	No. of interventions/outcomes	*I*^2^ (%)	*P*-heterogeneity	*P* between-subgroup heterogeneity
All RCTs	0.561	0.256, 0.865	33	90.69	<0.001	
RCT conducted on healthy older adults	0.113	−0.042, 0.268	17	30.22	0.115	0.000
RCT conducted on sarcopenic/frail older adults	1.211	0.588, 1.834	16	95.30	<0.001	
RCT measuring SPPB	0.856	−0.029, 1.741	7	94.55	<0.001	0.135
RCT measuring GS	1.000	0.218, 1.782	9	92.48	<0.001	
RCT measuring other physical tests	0.256	−0.073, 0.585	17	86.62	<0.001	
RCT with RE	0.055	−0.066, 0.175	19	0.00	0.943	0.000
RCT without RE	1.551	0.834, 2.267	14	96.04	<0.001	
RCT with high dose of whey (>20 g/d)	0.040	−0.059, 0.140	26	0.00	0.502	0.000
RCT with low dose of whey (<20 g/d)	3.379	1.765, 4.994	7	97.96	<0.001	
RCT with high duration (>12 wk)	1.042	0.503, 1.582	18	94.80	<0.001	0.001
RCT with low duration (≤12 wk)	0.100	−0.048, 0.248	15	10.19	0.339	

Abbreviations: GS, gait speed; RCT, randomized controlled trial; RE, resistance exercise; SPPB, Short Physical Performance Battery; SMD, standardized mean difference.

### Effect of whey protein and vitamin D supplementation on LM

Contrary to the nonsignificant effect of whey protein supplementation on LM, whey protein plus vitamin D co-supplementation positively affected LM (SMD = 0.993; 95% CI: 0.112, 1.874; *n* = 11). Moreover, there was a significant main effect in subgrouping regarding study duration ≤12 weeks (SMD = 1.651; 95% CI: 0.118, 3.183; *n* = 7) (Supplemental Table S12; Supplemental Figure S21). However, no significant effect was observed after subgrouping participants (Supplemental Figure S22).

### Effect of whey protein and vitamin D supplementation on muscle strength

The effect of whey protein plus vitamin D supplementation on muscle strength was significant when all RCTs were analyzed (SMD = 2.005; 95% CI: 0.975, 3.035; *n* = 11) (Supplemental Table S13; Supplemental Figure S23). This effect was also found in the subgroups of HGS (SMD = 2.943; 95% CI: 1.431, 4.455; *n* = 8), without RE (SMD = 2.800; 95% CI: 1.075, 4.526; *n* = 5), and study duration ≤12 weeks (SMD = 3.078; 95% CI: 1.299, 4.857; *n* = 8). This effect was also seen in the participant subgroup, whereas the effect size in the healthy group was higher than that in the sarcopenic or frail group (SMD = 2.386. 95% CI 0.741, 4.032; *n* = 6, vs. SMD = 1.722; 95% CI: 0.170, 3.274; *n* = 5) (Supplemental Table S13; Supplemental Figure S24).

### Effect of whey protein and vitamin D supplementation on physical function

The evidence showed that whey protein and vitamin D supplementation positively affected physical function changes (SMD = 3.038; 95% CI: 2.196, 3.879; *n* = 18) (Supplemental Table S14; Supplemental Figure S25). A similar effect was also found in both participant subgroups, whereas the subgroup with healthy older adults showed a greater effect size than the subgroup with sarcopenic or frail older adults (SMD = 4.290; 95% CI: 2.713, 5.867; *n* = 10, vs. SMD = 1.666; 95% CI: 0.676, 2.656; *n* = 6) (Supplemental Table S14; Supplemental Figure S26). Moreover, the subgroup analysis considering SPPB (SMD = 7.195; 95% CI: 3.922, 10.469; *n* = 4), other physical tests (SMD = 3.187; 95% CI: 1.879, 4.494; *n* = 10), without RE (SMD = 4.572; 95% CI: 3.297, 5.847; *n* = 10), low dose of whey (SMD = 3.728; 95% CI: 2.390, 5.066; *n* = 12), and short study duration (SMD = 5.738; 95% CI: 3.791, 7.686; *n* = 11) also revealed significant effects. Contrary to the results obtained from the effect of whey protein on GS, whey protein plus vitamin D supplementation had no positive effect on GS.

### Risk of bias and heterogeneity of the included studies

The summary of the risk-of-bias analysis and traffic light figure of each domain of the risk-of-bias assessment are shown in Supplemental Figure S1A,B. The risk-of-bias analysis demonstrated that selection bias was reported in 6 studies, 5 studies with some concerns, and 1 high-risk study owing to the lack of information regarding randomization or allocation methods. Regarding performance bias, 6 studies were scored as having some concerns, and 2 had a high risk of bias. Regarding the third domain, 2 studies were identified with some risk of detection bias due to missing data. Almost half of the studies (15 RCTs) reported a risk of attrition bias due to a lack of information regarding outcome assessor awareness of the intervention received. Furthermore, all included studies provided clear results for all outcomes per the prespecified analysis plan and were considered at low risk of reporting bias. Heterogeneity for the main effects of whey protein supplementation alone and with vitamin D on all evaluated sarcopenia outcomes was considerably high (*I*^2^ > 80%; *P* < 0.0001) (TABLE 2, TABLE 3, TABLE 4). A potential source of heterogeneity is the difference between study participants, healthy [15,17,19,21,[24], [25], [26], [27],^30^,^31^,^33^,[36], [37], [38],^41^,^42^], sarcopenic [9,18,29,34,35,39,43], frail [16,32,40], and mobility-limited [20,22,23,28] older adults. However, differences in duration of the studies and the range of supplement doses tested might have contributed as sources of heterogeneity (Supplemental Table S1). After excluding studies from all analyses using the leave-1-out method, there were no significant changes in all our findings (data not shown).

### Quality of evidence

The GRADEpro evidence profile rating results for changes in sarcopenia measures with whey protein supplementation alone or with vitamin D in healthy older adults are presented in Supplemental Tables S2 and S3. In the studies reporting the effect of whey protein supplementation, the GRADE rating was found to be low and very low for included variables. Studies reporting the effects of whey protein and vitamin D supplementation were also of low and very low quality. The funnel plots for LM, muscle strength, muscle function, TLM, ALM, HGS, lower body strength, SPPB, GS, and other physical tests for whey protein supplementation and LM, muscle strength, and function for co-supplementation of whey protein and vitamin D are shown in Supplemental Figures S27–S39. Asymmetry was found in the pooled models.

## Discussion

We examined the effects of whey protein with and without vitamin D supplementation on measures relevant to sarcopenia. We found that whey protein supplementation did not increase LM in healthy older participants but did affect LM in sarcopenic or frail older persons. The result of this meta-analysis concurs with a recent analysis on protein supplementation, showing that the changes in LM were largely (if not exclusively) related to the effects of RE rather than protein supplementation [7]. Many trials have confirmed that recommending protein supplementation as a stand-alone intervention for older adults has no significant effects on muscle mass or strength [21,22,30,33]. Our results are noteworthy because older adults exhibit impaired muscle anabolism in response to protein intake [50,51]. However, in a subgroup analysis, whey protein supplementation significantly improved LM in sarcopenic or frail older adults (particularly ALM). Older adults with low LM may have higher requirements for protein, which could underpin the effects of whey protein in sarcopenic or frail persons [52].

The effect of vitamin D plus whey protein on increasing LM was significant. This result was pronounced in the subgroups with a study duration of ≤12 weeks. Bauer et al. [11] reported an effect of co-supplementation with whey protein with vitamin D on LM. We propose that correcting vitamin D deficiency or insufficiency likely underpins this co-supplementation effect [53]. Vitamin D can improve muscle mass through genomic and nongenomic pathways [54]. The main mechanisms of the anabolic effects of vitamin D on muscle anabolism are not fully understood; nevertheless, its positive influence on muscle protein anabolism has been shown [53,55].

Analyzing all included RCTs showed no significant change in muscle strength gains with whey protein supplementation; however, significant increases were seen in subgroups who engaged in RE and consumed whey protein doses higher than 20 g. In another meta-analysis, the effects of protein supplementation on muscle strength were mainly observed in RE-trained participants [56]. According to 2 other studies, improvement in muscle strength after whey protein ingestion was enhanced in those with RE, which is in line with the results of previous work [57,58]. Resistance training enhances functional capacity and muscle strength [59] due to the anabolic responses to RE separate from dietary supplementation [60,61]. Training or exercise is an important inducer of muscle hypertrophy, particularly in older adults [62]. Other studies have shown that higher protein doses of 30–35 g of whey protein may positively affect muscle protein synthesis [63,64], indicating that higher doses may be advantageous, particularly in older persons [65].

Our analysis showed that co-supplementation with whey protein and vitamin D significantly affected muscle strength gains. This finding is in contrast to the effect of whey protein supplementation alone. Significant results were also seen in the subgroups without RE in studies lasting 12 weeks or less, highlighting the effect of vitamin D supplementation in the short term. However, we did not observe an effect of supplementation when RE was used, which is due to the much greater influence of RE in improving LM and strength [66]. Because both the control and intervention groups were exposed to RE, the nutritional supplement-mediated effect is markedly attenuated, or completely obscured, by the RE training-mediated effect.

However, whey protein supplementation improved physical function significantly, which was mainly related to the changes in GS. Results were pronounced in the subgroups without RE, with lower doses of whey protein (<20 g), in sarcopenic or frail older adults, and studies with durations of >12 weeks. We are unsure how whey protein supplementation would promote such an effect, but it may relate to subtle changes in LM or a previously unrecognized mechanism.

Supplementation with vitamin D plus whey protein significantly improved physical function. This finding was also confirmed by a study reviewing the effect of nutrition supplementation (containing protein and vitamin D) on physical performance in older people [67]. In that meta-analysis, the effects of nutritional supplementation were mainly pronounced on physical outcomes rather than muscle strength [67]. In line with this study, in another review, vitamin D plus protein supplementation did not affect LM in sarcopenic or frail participants [68]. There is an idea that muscle mass and strength changes do not happen concurrently with changes in physical function. Instead, other mechanisms could improve physical function, such as neural adaptations, enhanced metabolic function, and nonhypertrophic remodeling of the contractile machinery [69,70]. In subgroup analyses, the change was significant in SPPB and other physical tests in the subgroups consuming lower doses of whey (<20 g), without RE, and in studies of shorter durations (<12 weeks). Physical function could be affected by protein or vitamin D ingestion. Optimizing vitamin D status is an important factor affecting muscle mass and function [71], and it can boost muscle strength and function by improving muscle anabolism when combined with leucine [53].

One of our main findings is that whey protein plus vitamin D affected muscle strength and physical function in the subgroup without RE. This result contrasts with some data showing a beneficial effect of whey protein plus RE [72]. However, this was not observed in our meta-analysis because the effects were seen in those without RE. The differences between our results and others [72] could be for various reasons. Our analysis showed that most of the effects of whey protein supplementation on muscle strength and function are shown in longer durations (>12 weeks) studies. However, the effects of whey protein plus vitamin D supplementation were observed in shorter-duration studies (≤12 weeks). We propose that these findings are related to the fact that short-term supplementation with vitamin D corrects deficient or insufficient levels of the vitamin and exerts its beneficial effects similarly rapidly [73]. Nonetheless, heterogeneity was found to be very high, and the overall quality of the evidence was low or very low for most outcomes in this domain.

We need to acknowledge the limitations of our meta-analysis. Various nonuniform muscle strength and physical function measures were used in different studies, making the comparison or interpretation unclear. The same is true for using multiple methods for measuring LM, which may have added to the high degree of heterogeneity in some domains we analyzed. Moderate to high heterogeneity seems to be a persistent finding in meta-analyses involving protein ingestion studies depending on the outcome; for example, an *I*^2^ of 72% for LM in Tagawa et al. [74]; an *I*^2^ of 20% for LM and 50%–60% for strength and physical function in Nunes et al. [7]; an *I*^2^ of 50%–56% for strength in Tagawa et al. [75]; and an *I*^2^ of 90% or more for strength and physical function in Chang et al. [15]. We also found asymmetry in the funnel plots of the pooled model, although this is likely related more to heterogeneity than publication bias. Subgroup-level analyses resulted in some domains containing fewer studies, and thus smaller populations, and should be interpreted cautiously. The overall quality of the evidence was mostly low or very low; hence, our findings need to be interpreted with this caveat in mind. A strength of our analysis was the comprehensive and simultaneous assessment of the effects of whey protein or combined whey and vitamin D supplementation on all sarcopenia measures in healthy and sarcopenic or frail older adults.

In summary, whey protein supplementation did not increase LM, even when combined with RE in healthy older adults but did improve LM (mainly ALM) in sarcopenic or frail older adults. Similarly, whey protein supplementation did not affect muscle strength, but improvements were seen with higher doses of whey (>20 g) when combined with RE. Longer duration (>12 weeks) whey protein supplementation significantly improved physical function, particularly GS, but the addition of RE superseded the effects of the protein supplementation and supplementation provided no further benefit. Nonetheless, in all domains, the quality of evidence was low. However, whey protein did affect sarcopenia-related measures when combined with a vitamin D supplement, showing improved LM in the short term (<12 weeks). Whey protein plus vitamin D co-supplementation increased LM, muscle strength, and physical function in non-RE supplementation. We hypothesize that this effect was due to correcting deficient/insufficient vitamin D status. Further studies are warranted to better elucidate the exact mechanisms of action of vitamin D combined with whey protein on sarcopenia measures and muscle protein anabolism, particularly in various durations or doses.

## Funding

There were no specific sources of funding for this work. EAN is a tier 2 Research Productivity Fellow supported by the Brazilian National Council for Scientific and Technological Development (CNPq) (grant number 308584/2019-8). SMP is a tier 1 Canada Research Chair and acknowledges the funding from that agency. SMP also holds grants from the National Science and Engineering Council (NSERC) of Canada (RGPIN-2020-06346) and the Canadian Institutes of Health Research (CIHR).

## Author disclosures

SMP reports grants or research contracts from the US National Dairy Council, Canadian Institutes for Health Research, Dairy Farmers of Canada, Roquette Freres, Ontario Centre of Innovation, Nestle Health Sciences, Myos, National Science and Engineering Research Council and the US NIH during the conduct of the study; personal fees from Nestle Health Sciences, and nonfinancial support from Enhanced Recovery, outside the submitted work. SMP has patents licensed to Exerkine but reports no financial gains from any patent or related work. The other authors have no conflicts of interest to report.

## Appendix A Supplementary data

Multimedia component1 Multimedia component1
