# Supplementary material for: Whey Protein Supplementation with or without Vitamin D on Sarcopenia-Related Measures: A Systematic Review and Meta-Analysis
Source: Adv Nutr. 2023 May 15;14(4):762–73. doi: 10.1016/j.advnut.2023.05.011 (PMC10334153; doi:10.1016/j.advnut.2023.05.011)
Supplement: Multimedia component1 [file mmc1.pdf]

SUPPLEMENTAL TABLES FOR

Whey protein supplementation with or without vitamin D on sarcopenia-related measures: A systematic review and meta-analysis

Nasrin Nasimi, [1,2] Zahra Sohrabi, [1,2] Everson A. Nunes, [3,4] Erfan Sadeghi, [5] Sanaz Jamshidi, [6] Zohreh Gholami,[1] Marzieh Akbarzadeh,[1,2] Shiva Faghih,[1,2] Masoumeh Akhlaghi, [1,2] and Stuart M. Phillips [3*]

[1] Department of Community Nutrition, School of Nutrition and Food Sciences, Shiraz University of Medical Sciences, Shiraz, Iran. [2] Nutrition Research Center, School of Nutrition and Food Sciences, Shiraz University of Medical Sciences, Shiraz, Iran. [3] Exercise Metabolism Research Group, Department of Kinesiology, McMaster University, Hamilton, Ontario, Canada. [4] Laboratory of Investigation of Chronic Diseases, Department of Physiological Sciences, Federal University of Santa Catarina, Florianópolis, Brazil. [5] Research Consultation Center (RCC), Shiraz University of Medical Sciences, Shiraz, Iran. [6] Department of Nutrition, School of Public Health, Iran University of Medical Sciences, Tehran, Iran.

Table S1 Characteristics of all included studies in the meta-analysis

Whey protein supplementation										
**Studies**	**Country**	**Study population**	**Age**	**Sex**	**Duration**	**Groups**	**Intervention**	**Dose whey**	**Dose vitamin D**	**Outcomes**
Azevedo et al. (2022) (1)	Brazil	T= 31 older adults	Mean 66.9 years	Mixed	12 wks.	EX+NUT<br>EX+PLA	NUT= whey<br>PLA= Maltodextrin	40	----	TLM<br>ALM<br>LS<br>FAT
Azhar et. al. (2022) (2)	USA	T= 61 older adults with low physical functioning	> 66 years	Mixed	12 wks.	NUT<br>CON	NUT= whey<br>CON= education only	15	----	TLM<br>FAT
Griffen et. al. (2022) (3)	UK	T= 36 healthy older adults	Mean= 67 years	Men	12 wks.	EX+NUT<br>EX+PLA<br>NUT<br>CON	NUT= whey<br>PLA= maltodextrin	25	----	TLM<br>ALM<br>LS<br>SPPB<br>GS<br>FAT
Roschel et. al (2021) (4)	Brazil	T= 66 pre- frail or frail older adults	Mean= 72 years	Wome n	16 wks.	EX+NUT<br>EX+PLA	NUT= Whey<br>PLA 2= Corn starch	30	----	TLM<br>ALM<br>HGS<br>LS<br>TUG<br>Time stand
Murphy et. al. (2021) (5)	Italy	T= 42 well-nourished older adults	> 60 years	Mixed	24 wks.	NUT<br>PLA	NUT= whey<br>PLA= isoenergetic control	21.2	----	ALM<br>HGS<br>LS<br>SPPB<br>GS<br>TUG<br>FAT
Mertz et. al. (2021) (6)	Denmark	T= 122 healthy older adults	> 60 years	Mixed	48 wks.	NUT<br>PLA	NUT: whey<br>PLA 1: CHO<br>PLA 2: collagen	20	----	TLM<br>LS<br>Walking (s)

Chunlei Li et. al. (2021) (7)	China	T= 62 older adults with low lean mass	65-79 years	Mixed	24 wks.	NUT<br>CON	NUT= whey<br>PLA 2= control	16	----	TLM<br>ALM<br>HGS<br>GS<br>SPPB<br>Chair stand
Dulac MC et. al. (2021) (8)	Canada	T= 60 community dwelling older adults	Mean= 69 years	Men	12 wks.	EX+NUT<br>EX+PLA	NUT=whey<br>PLA= maltodextrin	30	----	TLM<br>HGS<br>LS<br>GS<br>TUG<br>Walking (s)<br>Balance (s)<br>FAT
Boutry-Regard et. al. (2020) (9)	Switzerland	T= 27 older adults with limited mobility	Mean= 78 years	Mixed	12 wks.	EX+NUT<br>EX+PLA	NUT= whey<br>PLA=dextrin	20	----	TLM<br>LS<br>GS
Nabuco et. al. X (2019) (10)	Brazil	T= 30 healthy older adults	Mean= 68.8 years	Wome n	12 wks.	EX+NUT<br>EX	NUT= whey	35	----	ALM<br>LS
Nabuco et. al. (2019) (11)	Brazil	T= 26 older adults with sarcopenic obesity	> 60 years	Wome n	12 wks.	EX+NUT<br>EX	NUT= whey	35	----	TLM<br>ALM<br>LS<br>Walking (s)<br>FAT
Kirk et. al. (2019) (12, 13)	UK	T= 100 community dwelling older adults	60-86 years	Mixed	16 wks.	EX+NUT<br>EX<br>NUT<br>CON	NUT= whey		----	ALM<br>HGS<br>LS<br>SPPB<br>FAT

Bjo¨rkman et. al. (2019) (14)	Finland	T= 92 sarcopenic older adults	> 74 years	Mixed	48 wks.	EX+NUT<br>EX+PLA	NUT= whey<br>PLA= Iso- caloric	40	----	HGS<br>SPPB
Junior et. al. (2018) (15)	Brazil	T= 31 pre-conditioned older adults	Mean= 67.4 years	Women	12 wks.	EX+NUT<br>EX+PLA	NUT= whey<br>PLA= maltodextrin	35	----	ALM,<br>LS
Park Y et. al. (2018) (16)	Korea	T= 80 prefrail and frail older adults	70-85 years	Mixed	12 wks.	NUT<br>PLA	NUT= whey<br>PLA= maltodextrin		----	ALM<br>HGS<br>GS<br>SPPB<br>TUG<br>Sit to stand
Mori et. al. (2018) (17)	Japan	T= 50 healthy older adults	65-80 years	Women	24 wks.	EX+NUT<br>EX	NUT= whey	22.3	----	HGS<br>LS<br>GS
Holwerda et. al. (2018) (18)	Netherlands	T= 41 healthy older adults	Mean= 70 years	Men	12 wks.	EX+NUT<br>EX+PLA	NUT= whey<br>PLA= CHO	21	----	TLM<br>ALM<br>LS<br>SPPB<br>TUG<br>Walking (s)<br>Sit to stand<br>FAT
Stojkovic et. al. (2017) (19)	USA	T=84 post-menopausal women	Mean= 69 years	Women	72 wks.	NUT<br>PLA	NUT= whey<br>PLA= maltodextrin	20	----	TLM<br>FAT
Zhu et. al. (2015) (20)	Australia	T= 181 well-nourished healthy older women	70-80 years	Women	96 wks.	NUT<br>PLA	NUT= whey<br>PLA= CHO	30	----	ALM<br>HGS<br>LS<br>TUG

Chalé et. al. (2013) (21)	USA	T= 75 mobility-limited adults aged	70-85 years	Mixed	24 wks.	EX+NUT<br>EX+PLA	NUT= whey<br>PLA= Iso-caloric control	40	----	TLM<br>LS<br>GS<br>SPPB<br>Stair climb<br>Chair rise<br>FAT
Arnarson et. al. (2013) (22)	Iceland	T= 141 healthy older adults	65-91 years	Mixed	12 wks.	EX+NUT<br>EX+PLA	NUT= whey<br>PLA= Iso-caloric CHO	20	----	TLM<br>ALM<br>LS<br>TUG
Bjo¨rkman et. al. (2012) (23)	Finland	T= 97 nursing home residents	Mean= 83 years	Mixed	24 wks.	EX+NUT<br>EX+PLA	NUT= whey<br>PLA= non-isocaloric juice	20	----	TLM<br>HGS<br>LS
**Whey protein + vitamin D supplementation**										
Rondanelli et. al. (2020) (24)	Italy	T= 127 healthy old adults	≥65 years	Mixed	8 wks.	NUT<br>PLA	NUT= whey+ vit D<br>PLA= isocaloric maltodextrin	20	800 IU	ALM<br>HGS<br>SPPB<br>TUG<br>Chair stand
Lin et. al. (2020) (25)	Taiwan	T= 56 sarcopenic elders	> 65 years	Mixed	12 wks.	NUT<br>CON	NUT= whey+ vit D	8.5	120 IU	TLM<br>ALM<br>HGS<br>GS<br>FAT
Yamada et. al. (2019) (26)	Japan	T= 112 community-dwelling older adults	> 65 years	Mixed	12 wks.	EX+NUT<br>EX<br>NUT<br>CON	NUT= whey+ vit D<br>PLA= control	10	800 IU	ALM<br>HGS<br>LS<br>TUG

Englund et. al. (2017) (27, 28)	USA	T=117 Mobility-limited older adults	> 70 years	Mixed	24 wks.	EX+NUT<br>EX+PLA	NUT= whey+ vit D<br>PLA= noncaloric	20	800 IU	TLM<br>ALM<br>LS<br>GS<br>SPPB<br>FAT
Rondanelli et. al. (2016) (29)	Italy	T= 130 sarcopenic older adults	> 65 years	Mixed	12 wks.	EX+NUT<br>EX+PLA	NUT= whey+ vit D<br>PLA= Isocaloric maltodextrin	22	100 IU	TLM<br>HGS<br>LS<br>FAT
Molnár et. al. (2016) (30)	Hungary	T= 34 elderly with high risk for sarcopenia	Mean= 66years	Mixed	12 wks.	EX+NUT<br>EX	NUT= whey+ vit D	20	800 IU	ALM<br>HGS
Bauer et al. (2015) (31-34)	Belgium, Germany, Ireland, Italy, Sweden, and the UK	T= 380 sarcopenic older adults	> 65 years	Mixed	13 wks.	NUT<br>PLA	NUT= whey+ vit D<br>PLA= Isocaloric	40	800 IU	ALM<br>HGS<br>GS<br>Chair stand<br>SPPB
Verreijen et. al. (2014) (35)	Netherlands	T= 60 obese older adults	Mean= 63 years	Mixed	13 wks.	EX+NUT<br>EX+PLA	NUT= whey+ vit D<br>PLA= Isocaloric	20.7	800 IU	ALM<br>HGS<br>GS<br>Chair stand<br>FAT

Abbreviation: EX; exercise training, NUT; nutrition supplementation group, PLA; placebo group, CON; control group; TLM; Total Lean Mass, ALM; Appendicular Lean Mass, HGS; Handgrip Strength, LS; Lower body Strength, GS; Gait Speed, TUG; Time Up and Go, SPPB; Short Physical Performance Battery, s; second, wks.; weeks, IU; International Unit.

References:

1. de Azevedo Bach S, Radaelli R, Schemes MB, Neske R, Garbelotto C, Roschel H, et al. Can supplemental protein to low-protein containing meals superimpose on resistance-training muscle adaptations in older adults? A randomized clinical trial. Experimental Gerontology. 2022;162:111760.
2. Azhar G, Wei JY, Schutzler SE, Coker K, Gibson RV, Kirby MF, et al. Daily consumption of a specially formulated essential amino acid-based dietary supplement improves physical performance in older adults with low physical functioning. The Journals of Gerontology: Series A. 2021;76(7):1184-91.
3. Griffen C, Duncan M, Hattersley J, Weickert MO, Dallaway A, Renshaw D. Effects of resistance exercise and whey protein supplementation on skeletal muscle strength, mass, physical function, and hormonal and inflammatory biomarkers in healthy active older men: a randomised, double-blind, placebo-controlled trial. Experimental Gerontology. 2022;158:111651.
4. Roschel H, Hayashi AP, Fernandes AL, Jambassi-Filho JC, Hevia-Larraín V, de Capitani M, et al. Supplement-based nutritional strategies to tackle frailty: A multifactorial, double-blind, randomized placebo-controlled trial. Clinical Nutrition. 2021;40(8):4849-58.
5. Murphy CH, Flanagan EM, De Vito G, Susta D, Mitchelson KA, de Marco Castro E, et al. Does supplementation with leucine-enriched protein alone and in combination with fish-oil-derived n–3 PUFA affect muscle mass, strength, physical performance, and muscle protein synthesis in well-nourished older adults? A randomized, double-blind, placebo-controlled trial. The American journal of clinical nutrition. 2021;113(6):1411-27.
6. Mertz KH, Reitelseder S, Bechshøft R, Bulow J, Højfeldt G, Jensen M, et al. The effect of daily protein supplementation, with or without resistance training for 1 year, on muscle size, strength, and function in healthy older adults: A randomized controlled trial. The American journal of clinical nutrition. 2021;113(4):790-800.
7. Li C, Meng H, Wu S, Fang A, Liao G, Tan X, et al. Daily supplementation with whey, soy, or whey-soy blended protein for 6 months maintained lean muscle mass and physical performance in older adults with low lean mass. Journal of the Academy of Nutrition and Dietetics. 2021;121(6):1035-48. e6.
8. Dulac M, Pion C, Lemieux F, Carvalho LP, Boutros GEH, Bélanger M, et al. Effects of slow-v. fast-digested protein supplementation combined with mixed power training on muscle function and functional capacities in older men. British Journal of Nutrition. 2021;125(9):1017-33.
9. Boutry-Regard C, Vinyes-Parés G, Breuillé D, Moritani T. Supplementation with whey protein, omega-3 fatty acids and polyphenols combined with electrical muscle stimulation increases muscle strength in elderly adults with limited mobility: a randomized controlled trial. Nutrients. 2020;12(6):1866.
10. Nabuco HC, Tomeleri CM, Fernandes RR, Junior PS, Cavalcante EF, Venturini D, et al. Effects of protein intake beyond habitual intakes associated with resistance training on metabolic syndrome-related parameters, isokinetic strength, and body composition in older women. Journal of aging and physical activity. 2019;27(4):545-52.
11. Nabuco HC, Tomeleri CM, Fernandes RR, Junior PS, Cavalcante EF, Cunha PM, et al. Effect of whey protein supplementation combined with resistance training on body composition, muscular strength, functional capacity, and plasma-metabolism biomarkers in older women with sarcopenic obesity: A randomized, double-blind, placebo-controlled trial. Clinical nutrition ESPEN. 2019;32:88-95.

12. Kirk B, Mooney K, Cousins R, Angell P, Jackson M, Pugh JN, et al. Effects of exercise and whey protein on muscle mass, fat mass, myoelectrical muscle fatigue and health-related quality of life in older adults: a secondary analysis of the Liverpool Hope University—Sarcopenia Ageing Trial (LHU-SAT). European Journal of Applied Physiology. 2020;120(2):493-503.
13. Kirk B, Mooney K, Amirabdollahian F, Khaiyat O. Exercise and dietary-protein as a countermeasure to skeletal muscle weakness: Liverpool Hope University–Sarcopenia Aging Trial (LHU-SAT). Frontiers in Physiology. 2019:445.
14. Björkman MP, Suominen MH, Kautiainen H, Jyväkorpi SK, Finne-Soveri HU, Strandberg TE, et al. Effect of protein supplementation on physical performance in older people with sarcopenia–a randomized controlled trial. Journal of the American Medical Directors Association. 2020;21(2):226-32. e1.
15. Junior PS, Ribeiro AS, Nabuco HC, Fernandes RR, Tomeleri CM, Cunha PM, et al. Effects of whey protein supplementation associated with resistance training on muscular strength, hypertrophy, and muscle quality in preconditioned older women. International journal of sport nutrition and exercise metabolism. 2018;28(5):528-35.
16. Park Y, Choi J-E, Hwang H-S. Protein supplementation improves muscle mass and physical performance in undernourished prefrail and frail elderly subjects: a randomized, double-blind, placebo-controlled trial. The American journal of clinical nutrition. 2018;108(5):1026-33.
17. Mori H, Tokuda Y. Effect of whey protein supplementation after resistance exercise on the muscle mass and physical function o
f healthy older women: A randomized controlled trial. Geriatrics & gerontology international. 2018;18(9):1398-404.
18. Holwerda AM, Overkamp M, Paulussen KJ, Smeets JS, Van Kranenburg J, Backx EM, et al. Protein supplementation after exercise and before sleep does not further augment muscle mass and strength gains during resistance exercise training in active older men. The Journal of Nutrition. 2018;148(11):1723-32.
19. Stojkovic V, Simpson CA, Sullivan RR, Cusano AM, Kerstetter JE, Kenny AM, et al. The effect of dietary glycemic properties on markers of inflammation, insulin resistance, and body composition in postmenopausal American women: An ancillary study from a multicenter protein supplementation trial. Nutrients. 2017;9(5):484.
20. Zhu K, Kerr DA, Meng X, Devine A, Solah V, Binns CW, et al. Two-year whey protein supplementation did not enhance muscle mass and physical function in well-nourished healthy older postmenopausal women. The Journal of nutrition. 2015;145(11):2520-6.
21. Chalé A, Cloutier GJ, Hau C, Phillips EM, Dallal GE, Fielding RA. Efficacy of whey protein supplementation on resistance exercise–induced changes in lean mass, muscle strength, and physical function in mobility-limited older adults. Journals of Gerontology Series A: Biomedical Sciences and Medical Sciences. 2013;68(6):682-90.
22. Arnarson A, Gudny Geirsdottir O, Ramel A, Briem K, Jonsson P, Thorsdottir I. Effects of whey proteins and carbohydrates on the efficacy of resistance training in elderly people: double blind, randomised controlled trial. European journal of clinical nutrition. 2013;67(8):821-6.
23. Björkman M, Finne-Soveri H, Tilvis R. Whey protein supplementation in nursing home residents. A randomized controlled trial. European Geriatric Medicine. 2012;3(3):161-6.
24. Rondanelli M, Cereda E, Klersy C, Faliva MA, Peroni G, Nichetti M, et al. Improving rehabilitation in sarcopenia: a randomized-controlled trial utilizing a muscle-targeted food for special medical purposes. Journal of cachexia, sarcopenia and muscle. 2020;11(6):1535-47.
25. Lin C-C, Shih M-H, Chen C-D, Yeh S-L. Effects of adequate dietary protein with whey protein, leucine, and vitamin D supplementation on sarcopenia in older adults: An open-label, parallel-group study. Clinical nutrition. 2021;40(3):1323-9.

26. Yamada M, Kimura Y, Ishiyama D, Nishio N, Otobe Y, Tanaka T, et al. Synergistic effect of bodyweight resistance exercise and protein supplementation on skeletal muscle in sarcopenic or dynapenic older adults. Geriatrics & gerontology international. 2019;19(5):429-37.
27. Englund DA, Kirn DR, Koochek A, Zhu H, Travison TG, Reid KF, et al. Nutritional supplementation with physical activity improves muscle composition in mobility-limited older adults, the VIVE2 study: a randomized, double-blind, placebo-controlled trial. The Journals of Gerontology: Series A. 2018;73(1):95-101.
28. Fielding RA, Travison TG, Kirn DR, Koochek A, Reid KF, von Berens Å, et al. Effect of structured physical activity and nutritional supplementation on physical function in mobility-limited older adults: Results from the VIVE2 randomized trial. The journal of nutrition, health & aging. 2017;21(9):936-42.
29. Rondanelli M, Klersy C, Terracol G, Talluri J, Maugeri R, Guido D, et al. Whey protein, amino acids, and vitamin D supplementation with physical activity increases fat-free mass and strength, functionality, and quality of life and decreases inflammation in sarcopenic elderly. The American journal of clinical nutrition. 2016;103(3):830-40.
30. Molnár A, Jonasne Sztruhar I, Csontos ÁA, Ferencz C, Várbíró S, Székács B. Special nutrition intervention is required for muscle protective efficacy of physical exercise in elderly people at highest risk of sarcopenia. Physiology International (Acta Physiologica Hungarica). 2016;103(3):368-76.
31. Bauer JM, Verlaan S, Bautmans I, Brandt K, Donini LM, Maggio M, et al. Effects of a vitamin D and leucine-enriched whey protein nutritional supplement on measures of sarcopenia in older adults, the PROVIDE study: a randomized, double-blind, placebo-controlled trial. Journal of the American Medical Directors Association. 2015;16(9):740-7.
32. Verlaan S, Maier AB, Bauer JM, Bautmans I, Brandt K, Donini LM, et al. Sufficient levels of 25-hydroxyvitamin D and protein intake required to increase muscle mass in sarcopenic older adults–The PROVIDE study. Clinical Nutrition. 2018;37(2):551-7.
33. Liberman K, Njemini R, Luiking Y, Forti LN, Verlaan S, Bauer JM, et al. Thirteen weeks of supplementation of vitamin D and leucine-enriched whey protein nutritional supplement attenuates chronic low-grade inflammation in sarcopenic older adults: the PROVIDE study. Aging clinical and experimental research. 2019;31(6):845-54.
34. Hill TR, Verlaan S, Biesheuvel E, Eastell R, Bauer JM, Bautmans I, et al. A vitamin D, calcium and leucine-enriched whey protein nutritional supplement improves measures of bone health in sarcopenic non-malnourished older adults: the PROVIDE study. Calcified tissue international. 2019;105(4):383-91.
35. Verreijen AM, Verlaan S, Engberink MF, Swinkels S, de Vogel-van den Bosch J, Weijs PJ. A high whey protein–, leucine-, and vitamin D–enriched supplement preserves muscle mass during intentional weight loss in obese older adults: a double-blind randomized controlled trial. The American journal of clinical nutrition. 2015;101(2):279-86.

Table S2 GRADE evidence profile rating for sarcopenia measures' changes in studies testing whey protein supplementation in healthy older adults.

Certainty assessment							№ of patients		Effect		Certainty	Importance
**№ of studies**	**Study design**	**Risk of bias**	**Inconsistency**	**Indirectness**	**Imprecision**	**Other considerations**	**Whey protein supplementation**	**Control**	**Relative (95% CI)**	**Absolute (95% CI)**		
**Overall effects of whey protein supplementation on changes in Lean Mass (All RCTs)**												
20	randomised trials	serious[a]	very serious[b]	not serious	not serious	publication bias strongly suspected[c]	627	672	-	SMD **0.165 SD higher** (0.154 lower to 0.484 higher)	⊕◯◯◯ Very low	CRITICAL
**Overall effect of whey protein supplementation on changes in Muscle Strength (All RCTs)**												
20	randomised trials	serious[d]	very serious[b]	not serious	not serious	publication bias strongly suspected[c]	688	710	-	SMD **0.149 SD higher** (0.086 lower to 0.383 higher)	⊕⊕◯◯ Low	CRITICAL
**Overall effect of whey protein supplementation on changes in Physical Function (All RCTs)**												
16	randomised trials	not serious	very serious[b]	not serious	serious[f]	publication bias strongly suspected strong association dose response gradient[c,g]	580	586	-	SMD **0.561 SD higher** (0.256 higher to 0.865 higher)	⊕⊕◯◯ Low	CRITICAL
**Overall effect of whey protein supplementation on changes in Total Lean Mass (All RCTs)**												
14	randomised trials	serious[h]	very serious[b]	not serious	not serious	none	419	438	-	MD **0.069 lower** (0.499 lower to 0.362 higher)	⊕◯◯◯ Very low	IMPORTANT
**Overall effect of whey protein supplementation on changes in Appendicular Lean Mass (All RCTs)**												

Certainty assessment							№ of patients		Effect		Certainty	Importance
№ of studies	Study design	Risk of bias	Inconsistency	Indirectness	Imprecision	Other considerations	Whey protein supplementation	Control	Relative (95% CI)	Absolute (95% CI)		
13	randomised trials	not serious	very serious[b]	not serious	not serious	none	416	413	-	MD **0.166 higher** (0.093 lower to 0.426 higher)	⊕⊕◯◯ Low	IMPORTANT

Overall effect of whey protein supplementation on changes in Handgrip Strength (All RCTs)

10	randomised trials	serious[i]	very serious[b]	not serious	very serious[f]	none	374	380	-	MD **0.534 higher** (0.749 lower to 1.81 higher)	⊕◯◯◯ Very low	IMPORTANT

Overall effect of whey protein supplementation on changes in Lower body strength (All RCTs)

17	randomised trials	serious[j]	very serious[b]	not serious	serious[k]	none	519	528	-	MD **1.187 higher** (0.861 lower to 3.235 higher)	⊕◯◯◯ Very low	IMPORTANT

Overall effect of whey protein supplementation on changes in SPPB (All RCTs)

7	randomised trials	serious[m]	very serious[b]	not serious	not serious	publication bias strongly suspected dose response gradient[c]	221	216	-	MD **0.186 higher** (0.507 lower to 0.879 higher)	⊕◯◯◯ Very low	IMPORTANT

Overall effect of whey protein supplementation on changes in Gait Speed (All RCTs)

8	randomised trials	not serious	very serious[b]	not serious	serious[k]	dose response gradient	211	200	-	MD **0.061 higher** (0.001 lower to 0.122 higher)	⊕⊕◯◯ Low	IMPORTANT

Overall effect of whey protein supplementation on changes in Physical test (All RCTs)

Certainty assessment							№ of patients		Effect		Certainty	Importance
№ of studies	Study design	Risk of bias	Inconsistency	Indirectness	Imprecision	Other considerations	Whey protein supplementation	Control	Relative (95% CI)	Absolute (95% CI)		
11	randomised trials	serious[n]	very serious[b]	not serious	not serious	publication bias strongly suspected[c]	424	434	-	MD **0.209 higher** (0.434 lower to 0.853 higher)	⨁◯◯◯ Very low	IMPORTANT

Overall effect of whey protein supplementation on changes in Fat mass (All RCTs)

12	randomised trials	serious[o]	not serious	not serious	not serious	none	272	264	-	MD **0.033 lower** (0.465 lower to 0.398 higher)	⨁⨁⨁◯ Moderate	IMPORTANT

CI: confidence interval; **MD:** mean difference; **SMD:** standardised mean difference

Explanations

a. Low risk= 41.94%, Some concern= 51.61%, High risk= 6.45%
b. More than 75%
c. P-value for Egger test is significant.
d. Low risk= 37.5%, Some concern= 50%, High risk= 12.5%
e. Low risk= 38.46%, Some concern= 46.15%, High risk= 15.39%
f. After using sensitivity analysis, the significant association was disappeared.
g. P-value for Begg test is significant.
h. Low risk= 37.5%, Some concern= 50%, High risk= 12.5%
i. Low risk= 45%, Some concern= 36%, High risk= 18%
j. Low risk= 33.3%, Some concern= 57.18%, High risk= 9.52%
k. After using sensitivity analysis, the significant association was seen.
l. Low risk= 35%, Some concern= 55%, High risk= 10%
m. Low risk= 42.86%, Some concern= 57.14%
n. Low risk= 41%, Some concern= 59%
o. Low risk= 41.6%, Some concern= 50%, High risk= 8.4%

Table S3 GRADE evidence profile rating for sarcopenia measures' changes in studies testing whey protein and vitamin D supplementation in healthy older adults.

Certainty assessment							№ of patients		Effect		Certainty	Importance
№ of studies	Study design	Risk of bias	Inconsistency	Indirectness	Imprecision	Other considerations	Whey Protein and vitamin D supplementation	Control	Relative (95% CI)	Absolute (95% CI)		
**Overall effect of whey protein and vitamin D supplementation on changes in Muscle Mass (All RCTs)**												
8	randomised trials	serious[a]	very serious[b]	not serious	not serious	publication bias strongly suspected strong association[c]	480	480	-	SMD **0.993 SD higher** (0.112 higher to 1.874 higher)	⊕◯◯◯ Very low	CRITICAL
**Overall effect of whey protein and vitamin D supplementation on changes in Muscle Strength (All RCTs)**												
8	randomised trials	not serious	very serious[b]	not serious	very serious[e]	publication bias strongly suspected very strong association[c,f]	480	480	-	SMD **2.005 SD higher** (0.975 higher to 3.035 higher)	⊕◯◯◯ Very low	CRITICAL
**Overall effect of whey protein and vitamin D supplementation on changes in Physical Function (All RCTs)**												
7	randomised trials	not serious	very serious[b]	not serious	very serious[e]	publication bias strongly suspected very strong association dose response gradient[c,f]	463	463	-	SMD **3.038 SD higher** (2.196 higher to 3.879 higher)	⊕⊕◯◯ Low	CRITICAL

CI: confidence interval; **SMD:** standardised mean difference

Explanations

a. Low risk= 45.45%, Some concern= 27.275%, High risk= 27.275%
b. More than 75%
c. P-value for Egger test is significant.
d. High risk= 50%
e. After using sensitivity analysis, significant association was disappeared.
f. P-value for Begg test is significant.

Table S4 Effect of whey protein supplementation on changes in TLM

Groups/ subgroups	Effect size (MD)	95% CI	Number of intervention/ outcomes	I^2 (%)	*P* heterogeneity	*P* between-subgroup heterogeneity
**All RCTs**	**-0.069**	**-0.499 : 0.362**	**16**	**96.82**	**<0.001**	
RCT conducted on healthy older adults	-0.289	-0.437 : -0.140	9	58.15	0.014	0.757
RCT conducted on sarcopenic/frail older adults	0.239	-0.678 : 1.156	7	58.61	0.024	
RCTs with RE	-0.299	-0.618 : 0.021	10	0.00	0.838	0.246
RCTs without RE	0.100	-0.493 : 0.692	6	98.92	<0.001	
RCTs with high dose of whey (>20g/d)	-0.452	-0.927 : 0.023	8	0.00	1.000	0.141
RCTs with low dose of whey (<20g/d)	0.080	-0.446 : 0.607	8	98.51	<0.001	
RCTs with high duration (>12 weeks)	0.003	-0.545 : 0.551	5	99.14	<0.001	0.443
RCTs with low duration (<12 weeks)	-0.252	-0.603 : 0.100	11	0.00	0.915	

Table S5 Effect of whey protein supplementation on changes in ALM

Groups/ subgroups	Effect size (MD)	95% CI	Number of intervention/ outcomes	I^2 (%)	*P* heterogeneity	*P* between-subgroup heterogeneity
**All RCTs**	**0.166**	**-0.093 : 0.426**	**15**	**81.75**	**<0.001**	
RCT conducted on healthy older adults	-0.044	-0.180 : 0.091	11	0.00	0.902	0.204
RCT conducted on sarcopenic/frail older adults	0.564	0.520 : 0.609	4	0.00	0.506	
RCTs with RE	0.084	-0.130 : 0.298	9	5.43	0.390	0.522
RCTs without RE	0.226	-0.153 : 0.604	6	90.04	<0.001	
RCTs with high dose of whey (>20g/d)	0.051	-0.095 : 0.198	13	7.81	0.368	0.197
RCTs with low dose of whey (<20g/d)	0.367	-0.090 : 0.823	2	86.01	0.007	
RCTs with high duration (>12 weeks)	0.190	-0.192 : 0.573	6	90.30	<0.001	0.754
RCTs with low duration (<12 weeks)	0.117	-0.138 : 0.372	9	18.62	0.277	

Table S6 Effect of whey protein supplementation on changes in HGS

Groups/ subgroups	Effect size (MD)	95% CI	Number of intervention/ outcomes	I^2 (%)	*P* heterogeneity	*P* between-subgroup heterogeneity
**All RCTs**	**0.534**	**-0.742 : 1.810**	**11**	**90.15**	**<0.001**	
RCT conducted on healthy older adults	0.466	-0.415 : 1.348	6	0.00	0.964	0.412
RCT conducted on sarcopenic/frail older adults	0.557	-1.443 : 2.558	5	95.86	<0.001	
RCTs with RE	0.896	-0.977 : 2.769	6	81.67	<0.001	0.286
RCTs without RE	-0.205	-0.967 : 0.557	5	36.56	0.177	
RCTs with high dose of whey (>20g/d)	0.746	-0.545 : 2.038	9	76.71	<0.001	0.028
RCTs with low dose of whey (<20g/d)	-0.715	-0.911 : -0.520	2	0.00	0.374	
RCTs with high duration (>12 weeks)	0.722	-0.683 : 2.127	9	92.11	<0.001	0.362
RCTs with low duration (<12 weeks)	-0.562	-2.941 : 1.817	2	0.00	0.736	

Table S7 Effect of whey protein supplementation on changes in lower body strength (Knee extension and leg press)

Groups/ subgroups	Effect size (MD)	95% CI	Number of intervention/ outcomes	I^2 (%)	*P* heterogeneity	*P* between-subgroup heterogeneity
**All RCTs**	**1.187**	**-0.861 : 3.235**	**21**	**91.99**	**<0.001**	
RCT conducted on healthy older adults	1.351	-1.176 : 3.880	15	94.33	<0.001	0.250
RCT conducted on sarcopenic/frail older adults	0.573	-1.015 :2.161	6	0.00	0.796	
RCTs with RE	1.667	-0.977 : 4.311	15	87.03	<0.001	0.531
RCTs without RE	0.329	-2.912 : 3.569	6	95.55	<0.001	
RCTs with high dose of whey (>20g/d)	1.884	-0.302 : 4.070	16	87.08	<0.001	0.340
RCTs with low dose of whey (<20g/d)	-0.431	-4.650 : 3.789	5	95.68	<0.001	
RCTs with high duration (>12 weeks)	0.455	-1.865 : 2.775	11	90.22	<0.001	0.427
RCTs with low duration (<12 weeks)	2.103	-1.236 : 5.443	10	90.55	<0.001	

Table S8 Effect of whey protein supplementation on changes in SPPB

Groups/ subgroups	Effect size (MD)	95% CI	Number of intervention/ outcomes	I^2 (%)	*P* heterogeneity	*P* between-subgroup heterogeneity
**All RCTs**	**0.186**	**-0.507 : 0.879**	**7**	**94.88**	**<0.001**	
RCT conducted on healthy older adults	-0.050	-0.327 : 0.227	3	0.00	0.722	0.601
RCT conducted on sarcopenic/frail older adults	0.451	-0.390 : 1.293	4	91.50	<0.001	
RCTs with RE	0.047	-0.204 : 0.298	4	0.00	0.789	0.615
RCTs without RE	0.364	-0.846 : 1.574	3	93.74	<0.001	
RCTs with high dose of whey (>20g/d)	Not applicable					
RCTs with low dose of whey (<20g/d)						
RCTs with high duration (>12 weeks)	0.287	-0.513 : 0.879	5	95.23	<0.001	0.398
RCTs with low duration (<12 weeks)	-0.107	-0.549 : 0.335	2	0.00	0.581	

Table S9 Effect of whey protein supplementation on changes in GS

Groups/ subgroups	Effect size (MD)	95% CI	Number of intervention/ outcomes	I^2 (%)	*P* heterogeneity	*P* between-subgroup heterogeneity
**All RCTs**	**0.061**	**-0.001 : 0.122**	**9**	**90.56**	**<0.001**	
RCT conducted on healthy older adults	0.051	0.016 : 0.086	5	36.96	0.174	0.053
RCT conducted on sarcopenic/frail older adults	0.071	-0.041 : 0.185	4	83.43	0.0004	
RCTs with RE	0.034	0.004 : 0.064	5	0.00	0.711	0.106
RCTs without RE	0.102	0.026 : 0.178	4	87.97	<0.001	
RCTs with high dose of whey (>20g/d)	0.047	0.018 : 0.076	4	20.04	0.277	0.175
RCTs with low dose of whey (<20g/d)	0.135	0.011 : 0.259	5	46.06	0.173	
RCTs with high duration (>12 weeks)	0.057	-0.048 : 0.162	4	94.19	<0.001	0.892
RCTs with low duration (<12 weeks)	0.064	0.034 : 0.095	5	0.00	0.469	

Table S10 Effect of whey protein supplementation on changes in other physical tests

Groups/ subgroups	Effect size (MD)	95% CI	Number of intervention/ outcomes	I^2 (%)	*P* heterogeneity	*P* between-subgroup heterogeneity
**All RCTs**	**0.209**	**-0.434 : 0.853**	**17**	**98.00**	**<0.001**	
RCT conducted on healthy older adults	0.018	-0.128 : 0.165	9	0.00	0.677	0.524
RCT conducted on sarcopenic/frail older adults	0.264	-0.841 : 1.370	8	98.78	<0.001	
RCTs with RE	0.058	-0.048 : 0.164	10	0.00	0.988	0.420
RCTs without RE	0.571	-0.674 : 1.816	7	97.40	<0.001	
RCTs with high dose of whey (>20g/d)	0.029	-0.072 : 0.131	13	0.00	0.978	0.154
RCTs with low dose of whey (<20g/d)	1.049	-0.351 : 2.449	4	96.36	<0.001	
RCTs with high duration (>12 weeks)	0.372	-0.601 : 1.345	9	98.76	<0.001	0.507
RCTs with low duration (<12 weeks)	0.038	-0.129 : 0.205	8	0.00	0.999	

Table S11 Effect of whey protein supplementation on changes in fat mass

Groups/ subgroups	Effect size (MD)	95% CI	Number of intervention/ outcomes	I^2 (%)	*P* heterogeneity	*P* between-subgroup heterogeneity
**All RCTs**	**-0.033**	**-0.465 : 0.398**	**12**	**0.00**	**0.999**	
RCT conducted on healthy older adults	-0.021	-0.460 : 0.417	9	0.00	0.995	0.876
RCT conducted on sarcopenic/frail older adults	-0.373	-2.742 : 1.996	3	0.00	0.983	
RCTs with RE	-0.089	-0.611 : 0.432	7	0.00	0.998	0.706
RCTs without RE	0.089	-0.680 : 0.859	5	0.00	0.943	
RCTs with high dose of whey (>20g/d)	-0.022	-0.464 : 0.419	9	0.00	0.999	0.822
RCTs with low dose of whey (<20g/d)	-0.261	-2.291 : 1.768	3	0.00	0.745	
RCTs with high duration (>12 weeks)	0.090	-0.666 : 0.846	5	0.00	0.949	0.696
RCTs with low duration (<12 weeks)	-0.093	-0.619 : 0.433	7	0.00	0.997	

Table S12 Effect of whey protein and vitamin D supplementation on changes in LM

Groups/ subgroups	Effect size (SMD)	95% CI	Number of interventions/ outcomes	I^2 (%)	*P* heterogeneity	*P* between-subgroup heterogeneity
**All RCT**	**0.993**	**0.112 : 1.874**	**11**	**97.68**	**<0.001**	
RCT conducted on healthy older adults	1.135	-0.430 : 2.701	4	97.17	<0.001	0.027
RCT conducted on sarcopenic/frail older adults	0.911	-0.197 : 2.020	7	97.92	<0.001	
RCT measuring TLM	1.920	-1.972 : 5.811	3	99.28	<0.001	0.541
RCT measuring ALM	0.688	-0.005 : 1.380	8	95.18	<0.001	
RCT with RE	1.133	-0.452 : 2.719	6	98.27	<0.001	0.783
RCT without RE	0.860	-0.277 : 1.996	5	97.17	<0.001	
RCT with high dose of whey (>20g/d)	2.537	-0.356 : 5.430	3	99.07	<0.001	0.180
RCT with low dose of whey (<20g/d)	0.452	-0.514 : 1.418	8	96.74	<0.001	
RCT with high duration (>12 weeks)	-0.088	-0.916 : 0.741	4	95.59	<0.001	0.051
RCT with low duration (≤12 weeks)	1.651	0.118 : 3.183	7	97.98	<0.001	

Table S13 Effect of whey protein and vitamin D supplementation on changes in muscle strength

Groups/ subgroups	Effect size (SMD)	95% CI	Number of interventions/ outcomes	I^2 (%)	*P* heterogeneity	*P* between-subgroup heterogeneity
**All RCT**	**2.005**	**0.975 : 3.035**	**11**	**97.93**	**<0.001**	
RCT conducted on healthy older adults	2.386	0.741 : 4.032	6	97.95	<0.001	0.000
RCT conducted on sarcopenic/frail older adults	1.722	0.170 : 3.274	5	98.32	<0.001	
RCT measuring upper-body strength	2.943	1.431 : 4.455	8	98.50	<0.001	0.000
RCT measuring lower-body strength	-0.009	-0.540 : 0.521	3	74.29	0.020	
RCT with RE	1.478	-0.050 : 3.006	6	97.92	<0.001	0.261
RCT without RE	2.800	1.075 : 4.526	5	98.36	<0.001	
RCT with high dose of whey (>20g/d)	3.015	-0.073 : 6.102	3	99.12	<0.001	0.416
RCT with low dose of whey (<20g/d)	1.645	0.475 : 2.814	8	97.25	<0.001	
RCT with high duration (>12 weeks)	-0.081	-0.348 : 0.185	3	44.63	0.164	0.001
RCT with low duration (≤12 weeks)	3.078	1.299 : 4.857	8	98.46	<0.001	

Table S14 Effect of whey protein and vitamin D supplementation on changes in physical function

Groups/ subgroups	Effect size (SMD)	95% CI	Number of intervention / outcomes	I^2 (%)	*P* heterogeneity	*P* between-subgroup heterogeneity
**All RCT**	**3.038**	**2.196 : 3.879**	**18**	**98.44**	**<0.001**	
RCT conducted on healthy older adults	4.290	2.713 : 5.867	10	98.62	<0.001	0.000
RCT conducted on sarcopenic/frail older adults	1.666	0.676 : 2.656	6	98.37	<0.001	
RCT measuring SPPB	7.195	3.922 : 10.469	4	99.39	<0.001	0.000
RCT measuring GS	-0.031	-0.406 : 0.344	4	74.03	0.009	
RCT measuring other physical tests	3.187	1.879 : 4.494	10	98.33	<0.001	
RCT with RE	1.350	0.277 : 2.424	8	97.34	<0.001	0.000
RCT without RE	4.572	3.297 : 5.847	10	98.87	<0.001	
RCT with high dose of whey (>20g/d)	2.161	1.025 : 3.297	6	98.47	<0.001	0.080
RCT with low dose of whey (<20g/d)	3.728	2.390 : 5.066	12	98.54	<0.001	
RCT with high duration (>12 weeks)	0.197	-0.358 : 0.752	7	95.36	<0.001	0.000
RCT with low duration (≤12 weeks)	5.738	3.791 : 7.686	11	98.94	<0.001	

SUPPLEMENTAL FIGURES FOR

Whey protein supplementation with or without vitamin D on sarcopenia-related measures: A systematic review and meta-analysis

Nasrin Nasimi, [1,2] Zahra Sohrabi, [1,2] Everson A. Nunes, [3,4] Erfan Sadeghi, [5] Sanaz Jamshidi, [6] Zohreh Gholami,[1] Marzieh Akbarzadeh,[1,2] Shiva Faghih,[1,2] Masoumeh Akhlaghi, [1,2] and Stuart M. Phillips [3*]

[1] Department of Community Nutrition, School of Nutrition and Food Sciences, Shiraz University of Medical Sciences, Shiraz, Iran. [2] Nutrition Research Center, School of Nutrition and Food Sciences, Shiraz University of Medical Sciences, Shiraz, Iran. [3] Exercise Metabolism Research Group, Department of Kinesiology, McMaster University, Hamilton, Ontario, Canada. [4] Laboratory of Investigation of Chronic Diseases, Department of Physiological Sciences, Federal University of Santa Catarina, Florianópolis, Brazil. [5] Research Consultation Center (RCC), Shiraz University of Medical Sciences, Shiraz, Iran. [6] Department of Nutrition, School of Public Health, Iran University of Medical Sciences, Tehran, Iran.

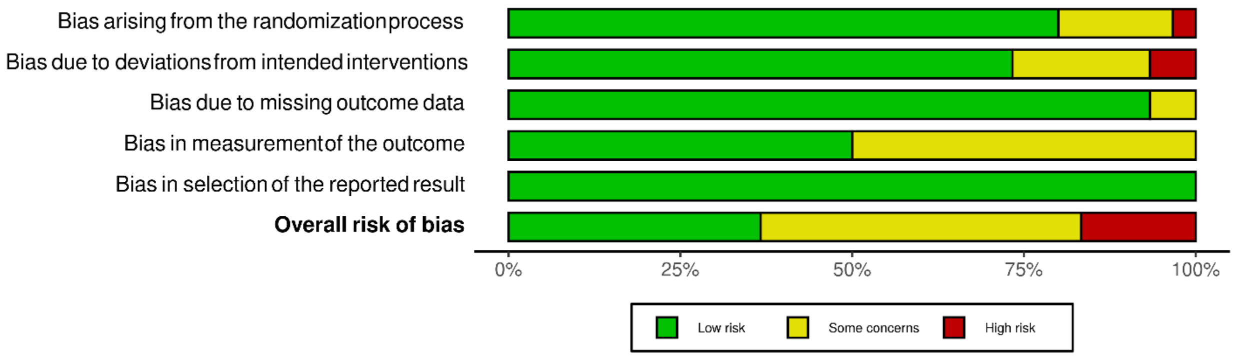

Figure S1a – Summary of risk of bias analysis showing the percentage of studies with potential some concerns or high risk of selection, performance, detection, attrition, reporting, or other bias.

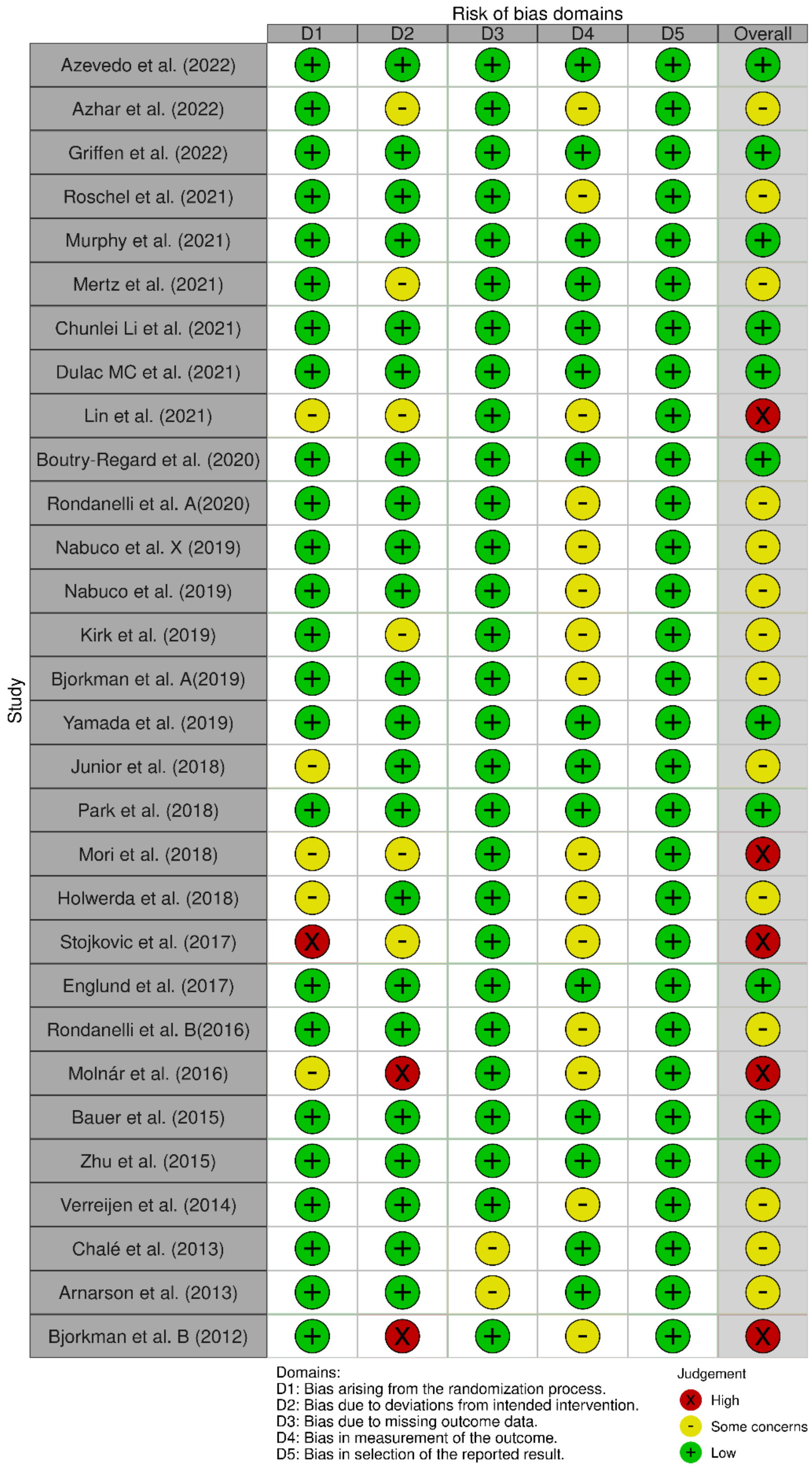

Figure S1b – Risk of bias analysis of all studies included in the meta-analysis.
(+) Circles filled in green = Low risk of bias
(-) Circles filled in yellow = Some concerns
(×) Circles filled in red = High risk of bias

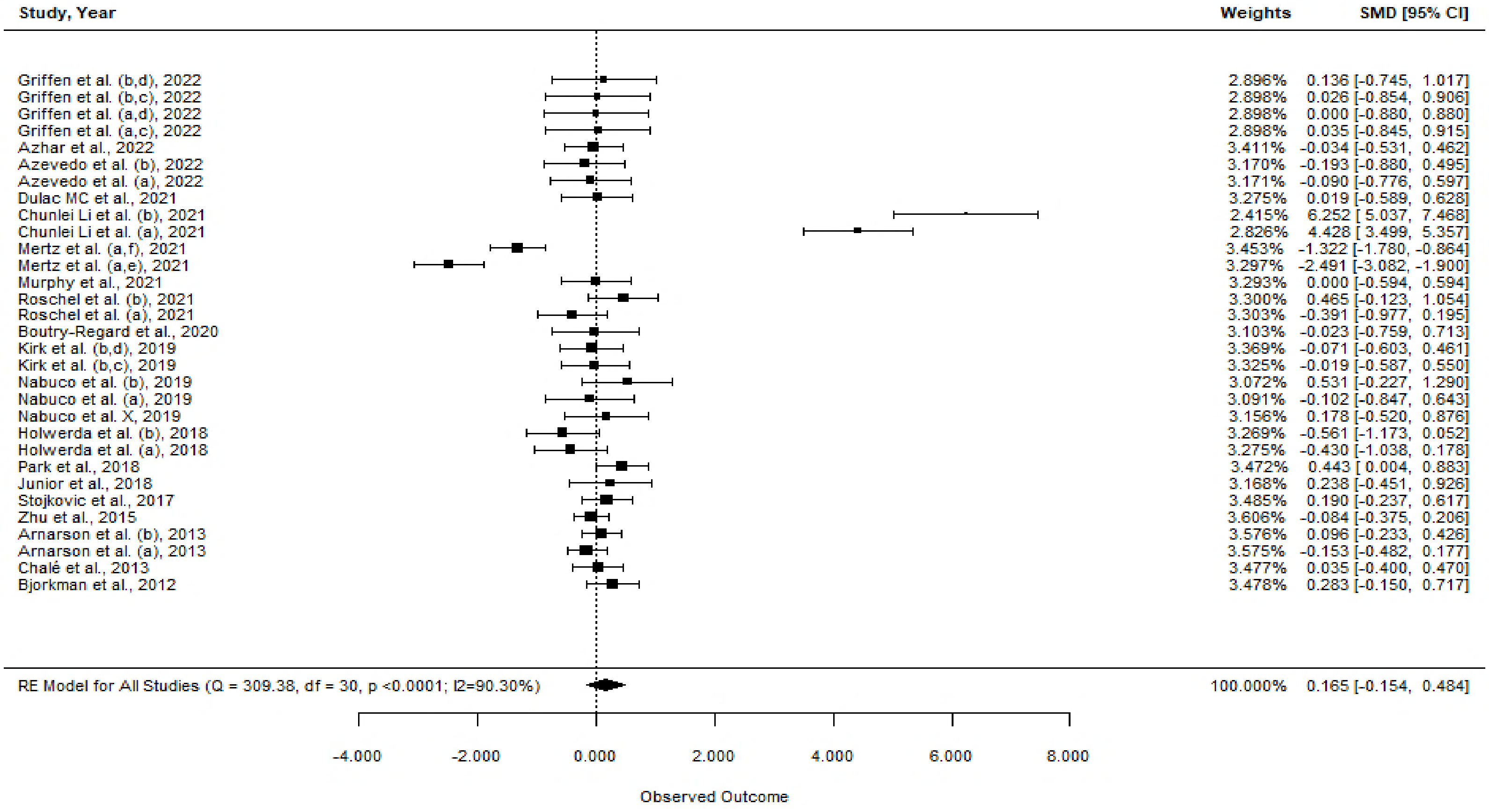

Figure S2. Forest plot of the Randomized Clinical Trials (RCTs) examining the effect of whey protein supplementation on LM. Data have been expressed as SMDs between intervention and control groups with 95% CI. Estimates were pooled using the random- effects, Hedges model. Letters between parentheses represent: a: TLM, b: ALM, c: with exercise training, d: without exercise training, e: control=CHO, f: control= collagen. Abbreviations: SMD= Standard Mean Difference, CI= Confidence Interval, TLM= Total Lean Mass, ALM= Appendicular Lean Mass.

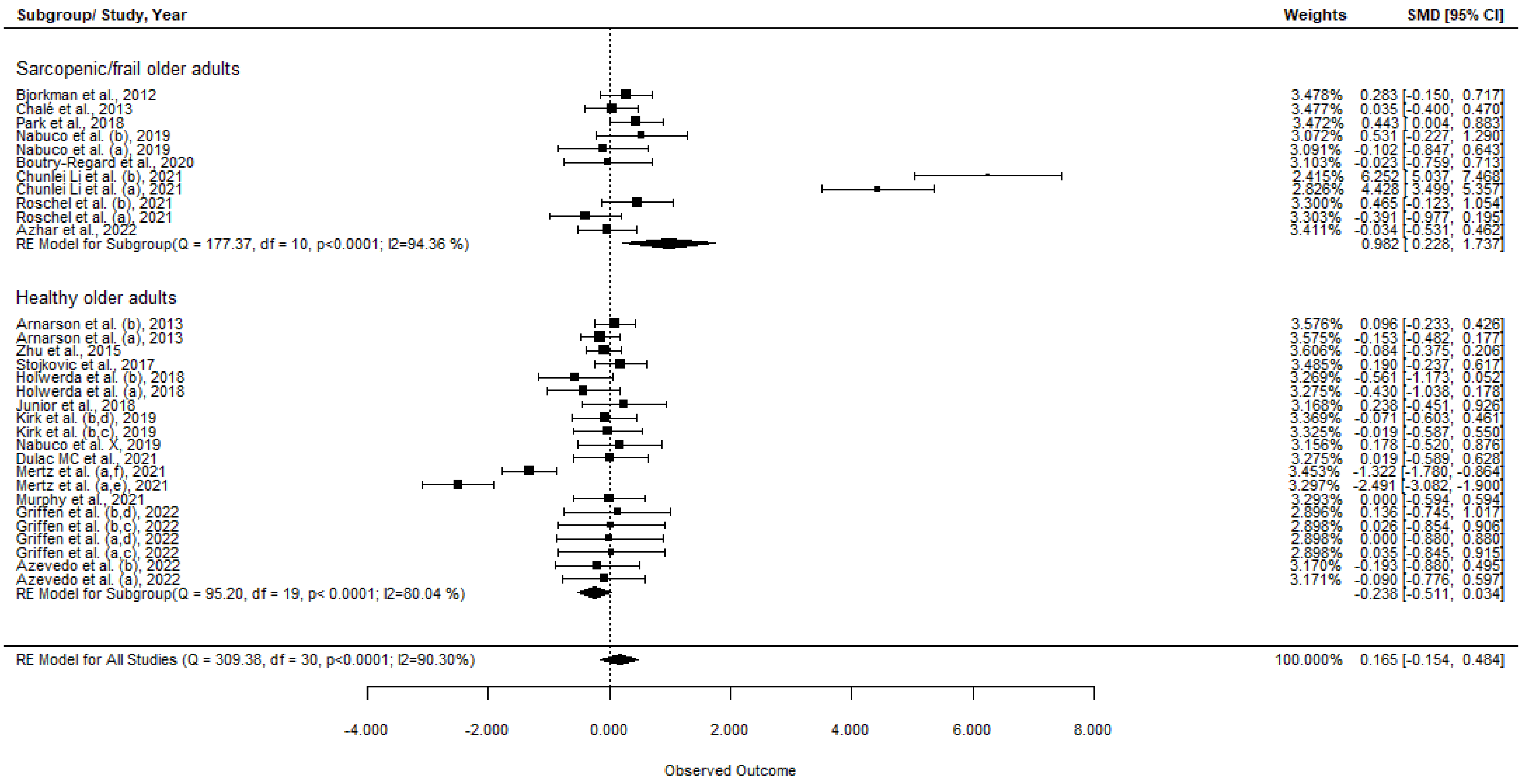

Figure S3. Forest plot of the Randomized Clinical Trials (RCT) examining the effect of whey protein supplementation on LM (subgrouping sarcopenic/frail and healthy older adults). Data are expressed as standardized mean differences (SMDs) between intervention and control groups with 95% CI. Estimates were pooled using the random-effects Hedges model. Letters between parentheses represent: a: TLM, b: ALM, c: with exercise training, d: without exercise training. SMD, Standard Mean Difference, CI, Confidence Interval, TLM, Total Lean Mass, ALM, Appendicular Lean Mass.

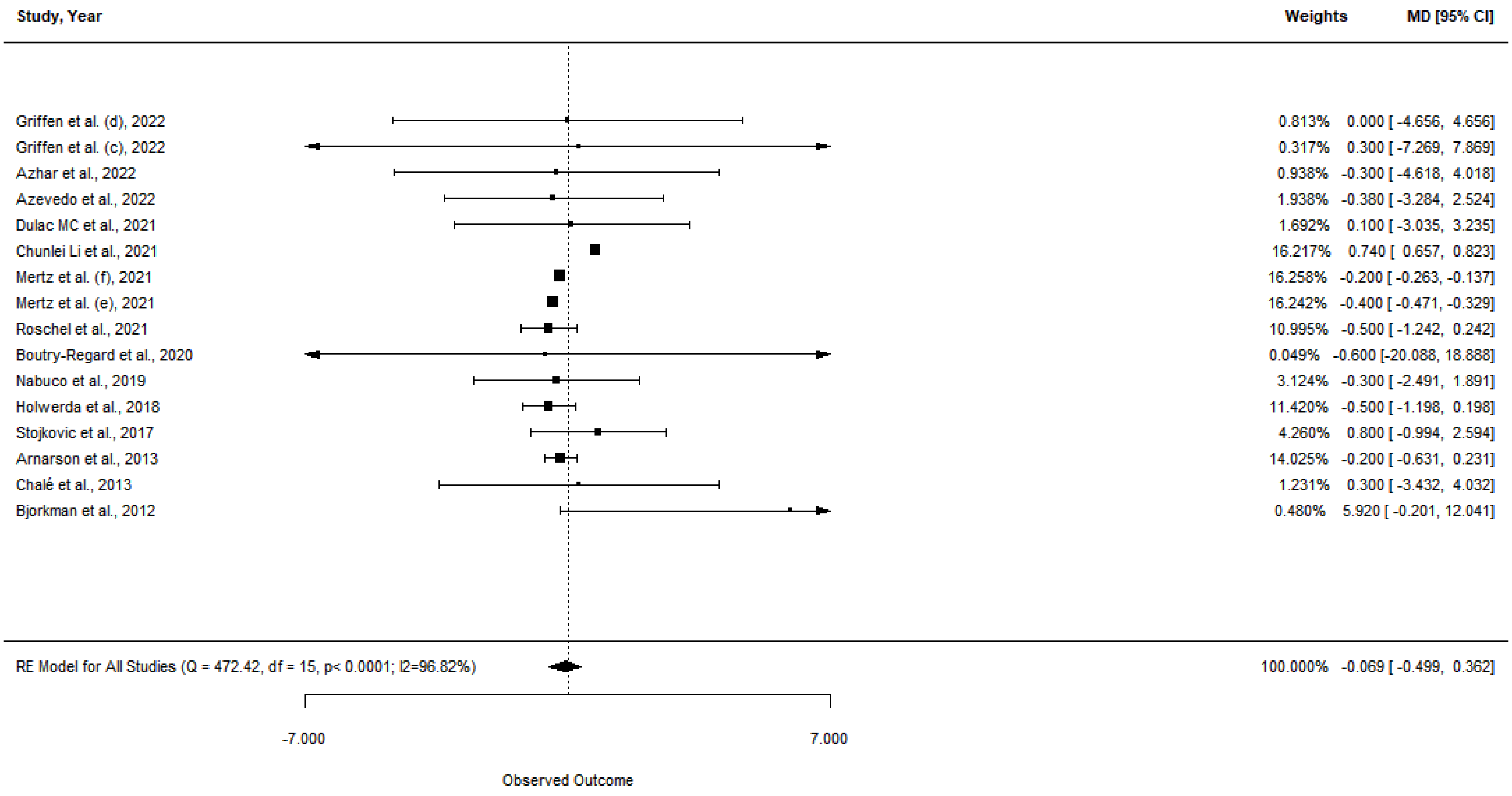

Figure S4. Forest plot of the Randomized Clinical Trials (RCTs) examining the effect of whey protein supplementation on TLM. Data have been expressed as MDs between intervention and control groups with 95% CI. Estimates were pooled using the random- effects. Letters between parentheses represent: c: with exercise training, d: without exercise training, e: control=CHO, f: control= collagen. Abbreviations: MD= Mean Difference, CI= Confidence Interval.

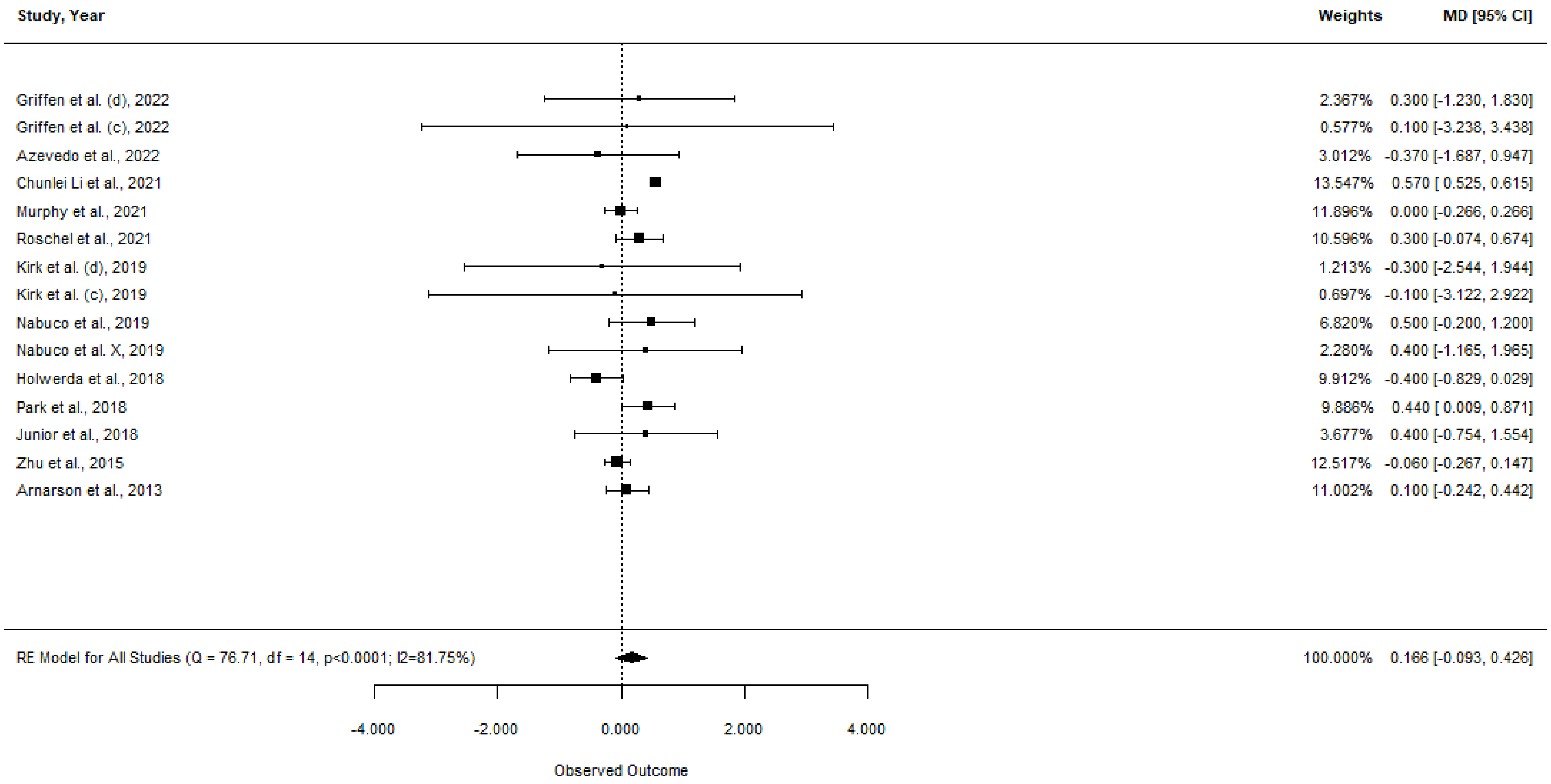

Figure S5. Forest plot of the Randomized Clinical Trials (RCTs) examining the effect of whey protein supplementation on ALM. Data have been expressed as MDs between intervention and control groups with 95% CI. Estimates were pooled using the random- effects. Letters between parentheses represent: c: with exercise training, d: without exercise training. Abbreviations: MD= Mean Difference, CI= Confidence Interval.

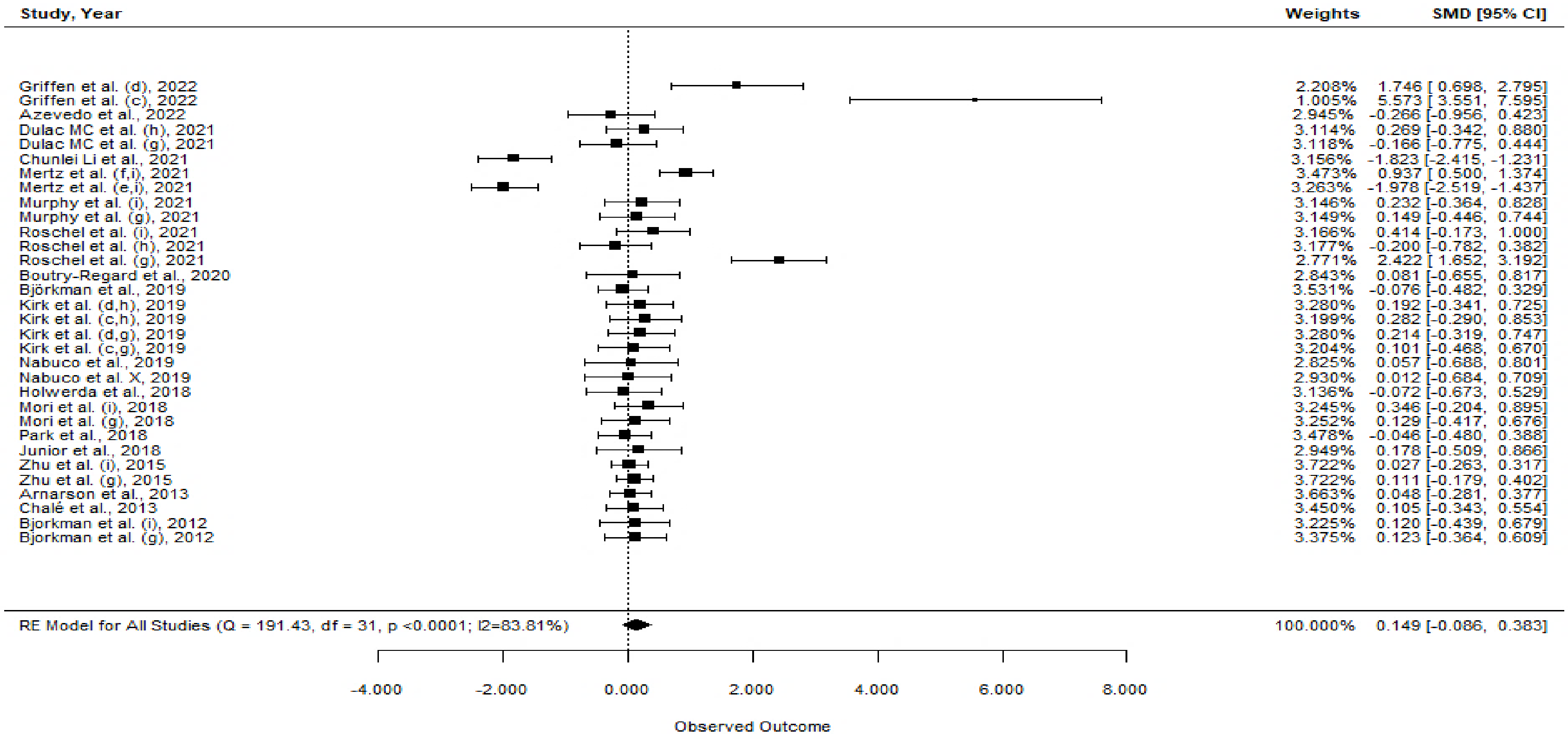

Figure S6. Forest plot of the Randomized Clinical Trials (RCTs) examining the effect of whey protein supplementation on muscle strength. Data have been expressed as SMDs between intervention and control groups with 95% CI. Estimates were pooled using the random- effects, Hedges model. Letters between parentheses represent: c: with exercise training, d: without exercise training, e: control=CHO, f: control= collagen, g= HGS, h= Leg Press, i= Knee Extension. Abbreviations: SMD= Standard Mean Difference, CI= Confidence Interval, HGS= handgrip strength.

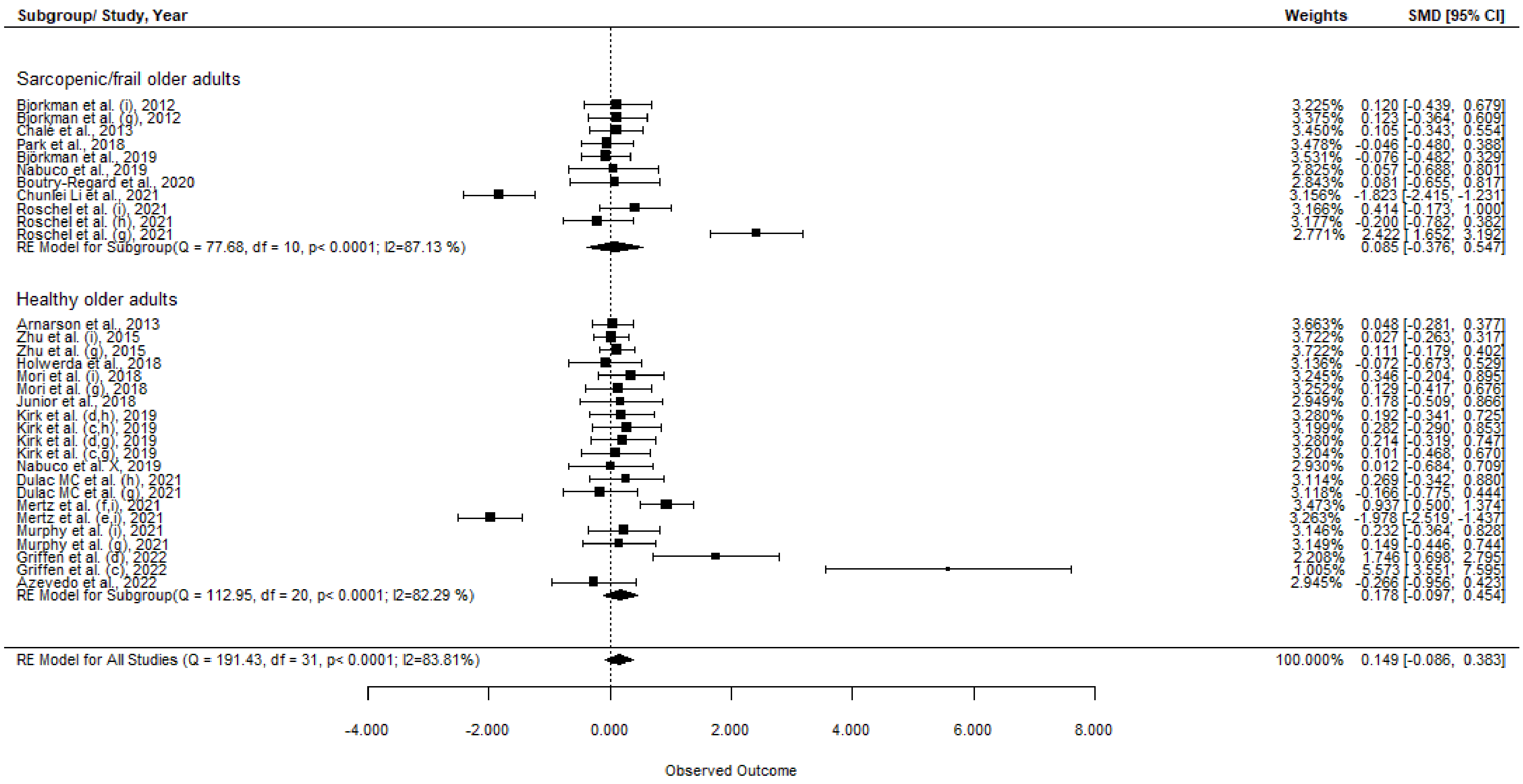

Figure S7. Forest plot of the Randomized Clinical Trials (RCT) examining the effect of whey protein supplementation on muscle strength (subgrouping sarcopenic/frail and healthy older adults). Data have been expressed as SMDs between intervention and control groups with 95% CI. Estimates were pooled using the random effects Hedges model. Letters between parentheses represent: c: with exercise training, d: without exercise training, f: control= collagen, g = HGS, h = Leg Press, I = Knee Extension. Abbreviations: SMD-Standard Mean Difference, CI-Confidence Interval, HGS-handgrip strength.

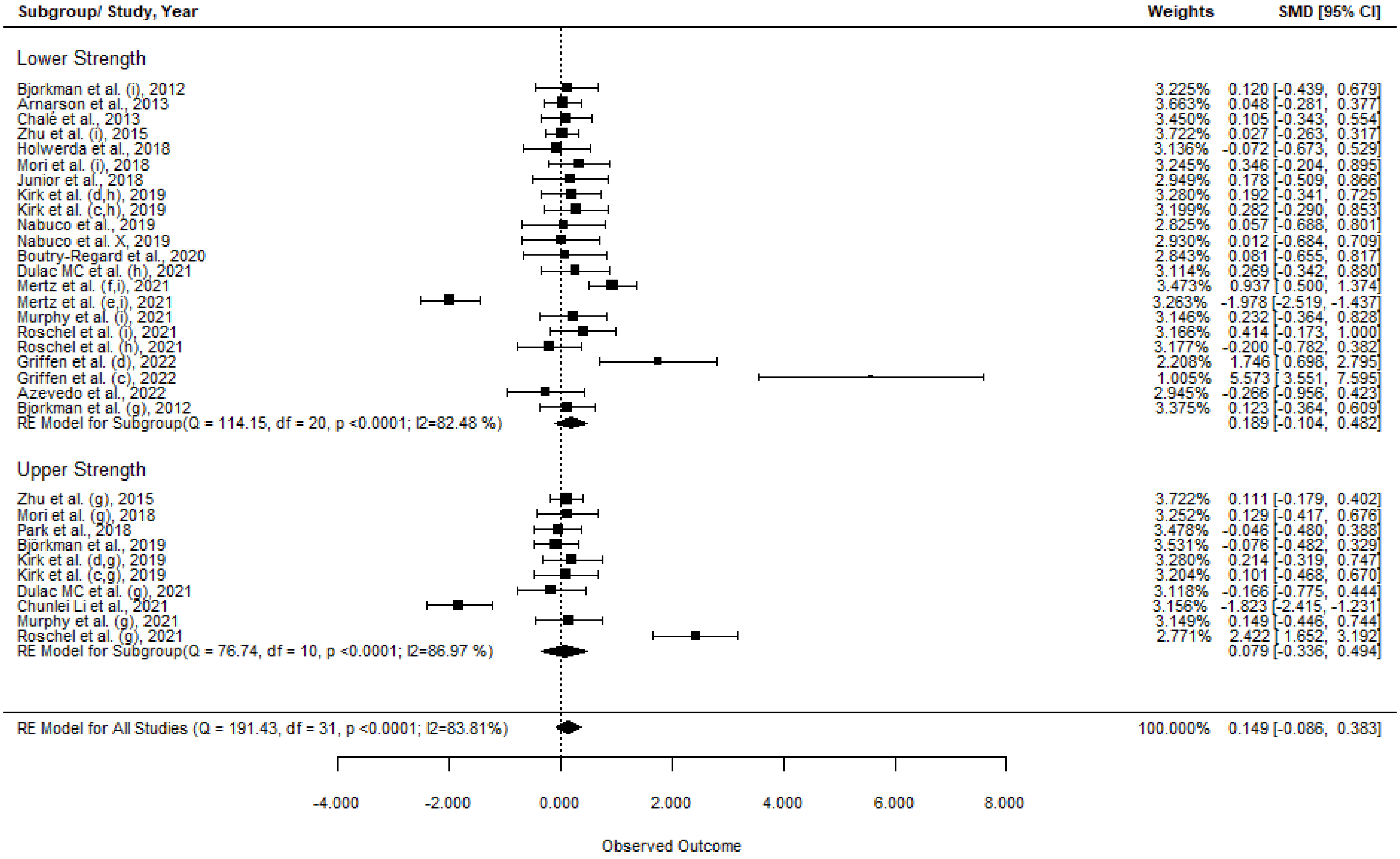

Figure S8. Forest plot of the Randomized Clinical Trials (RCT) examining the effect of whey protein supplementation on muscle strength (subgrouping upper and lower muscle strength). Data have been expressed as SMDs between intervention and control groups with 95% CI. Estimates were pooled using the random- effects, Hedges model. Letters between parentheses represent: c: with exercise training, d: without exercise training, f: control= collagen, g= HGS, h= Leg Press, i= Knee Extension. Abbreviations: SMD= Standard Mean Difference, CI= Confidence Interval, HGS= handgrip strength.

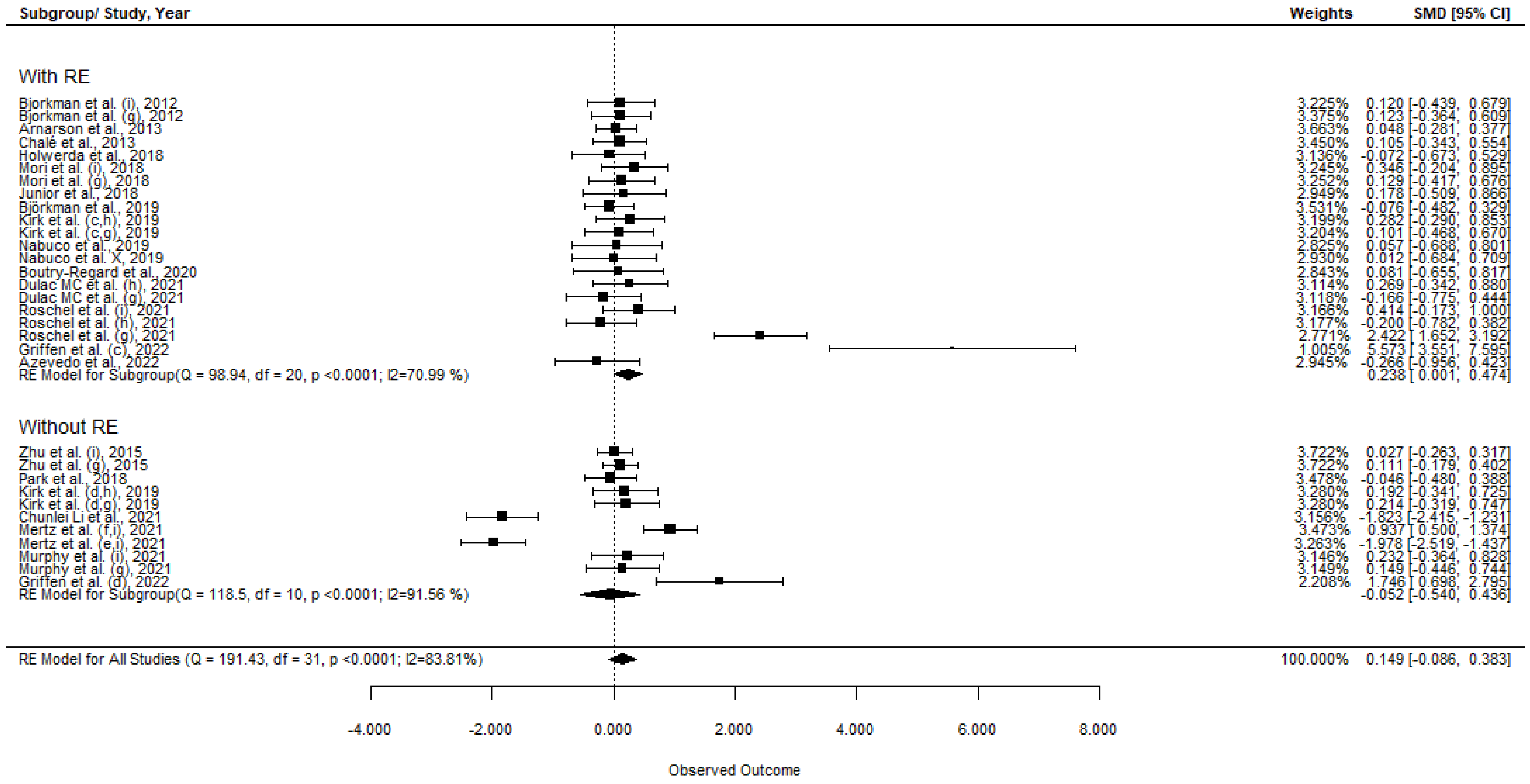

Figure S9. Forest plot of the Randomized Clinical Trials (RCTs) examining the effect of whey protein supplementation on muscle strength (subgrouping resistance exercise (RE)). Data have been expressed as SMDs between intervention and control groups with 95% CI. Estimates were pooled using the random- effects, Hedges model. Letters between parentheses represent: c: with exercise training, d: without exercise training, e: control=CHO, f: control= collagen, g= HGS, h= Leg Press, i= Knee Extension. Abbreviations: SMD= Standard Mean Difference, CI= Confidence Interval, HGS= handgrip strength.

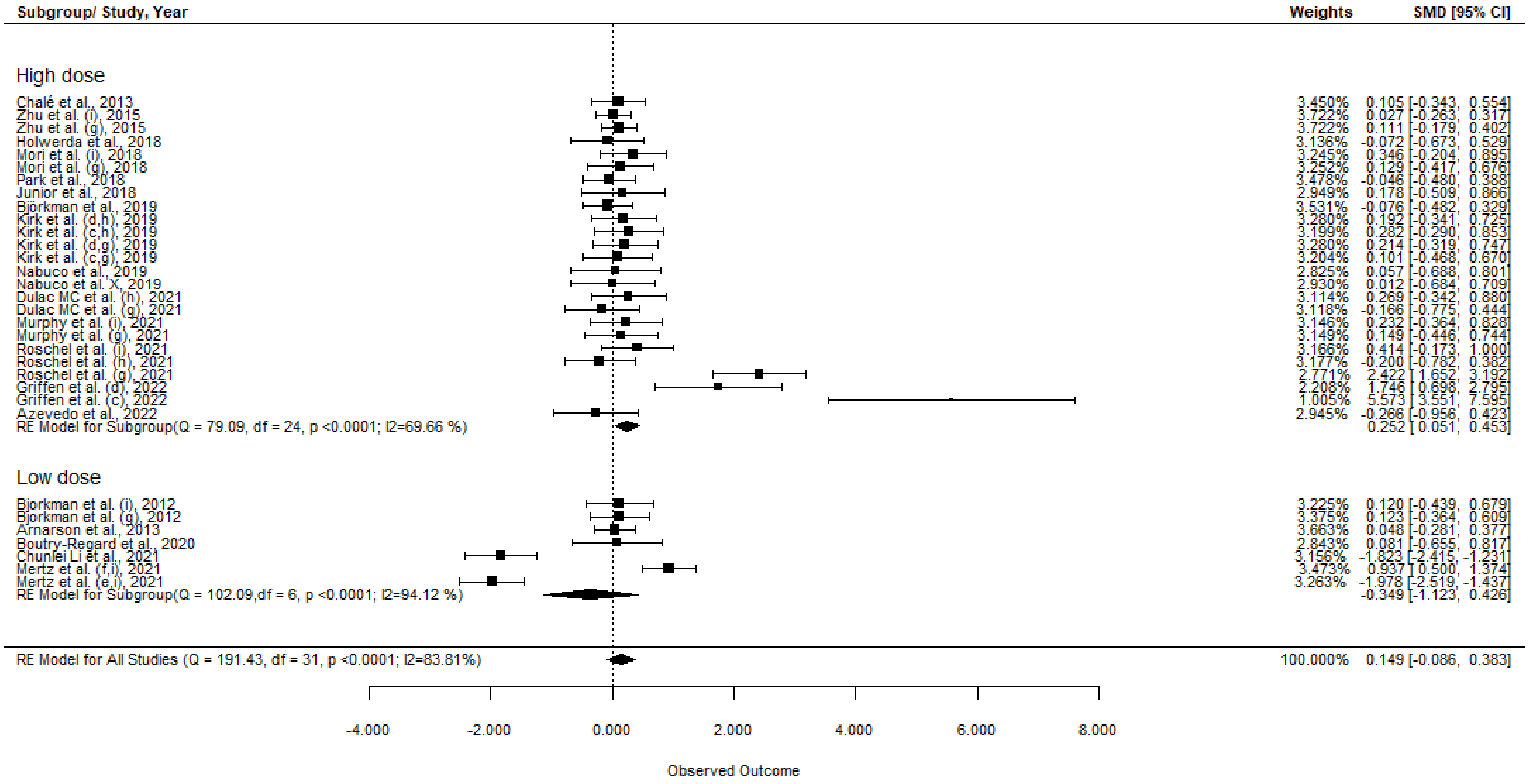

Figure S10. Forest plot of the Randomized Clinical Trials (RCTs) examining the effect of whey protein supplementation on muscle strength (subgrouping dose of whey). Data have been expressed as SMDs between intervention and control groups with 95% CI. Estimates were pooled using the random- effects, Hedges model. Letters between parentheses represent: c: with exercise training, d: without exercise training, e: control=CHO, f: control= collagen, g= HGS, h= Leg Press, i= Knee Extension. Abbreviations: SMD= Standard Mean Difference, CI= Confidence Interval, HGS= handgrip strength.

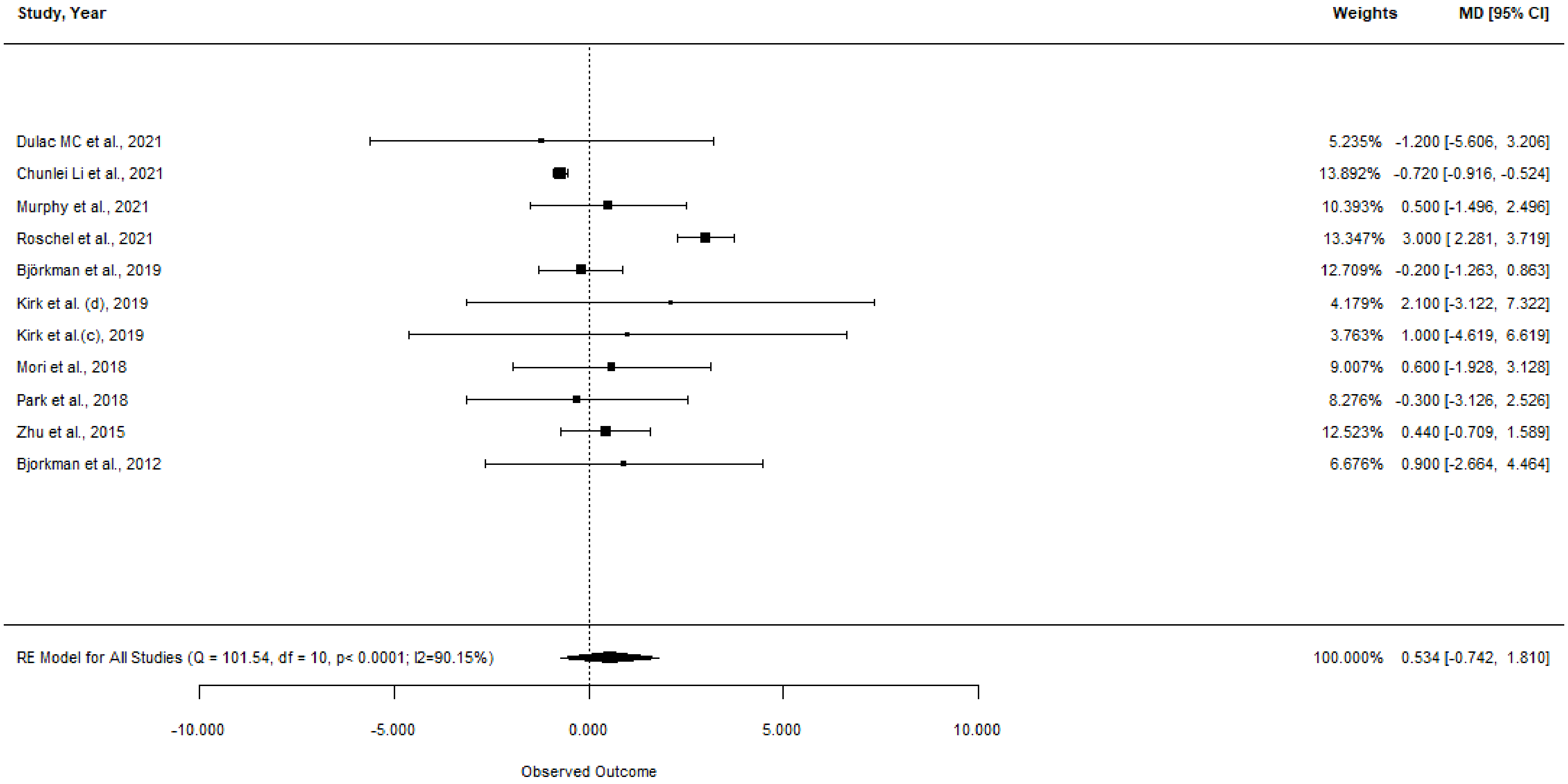

Figure S11. Forest plot of the Randomized Clinical Trials (RCTs) examining the effect of whey protein supplementation on handgrip strength. Data have been expressed as MDs between intervention and control groups with 95% CI. Estimates were pooled using the random- effects. Letters between parentheses represent: c: with exercise training, d: without exercise training. Abbreviations: MD= Mean Difference, CI= Confidence Interval.

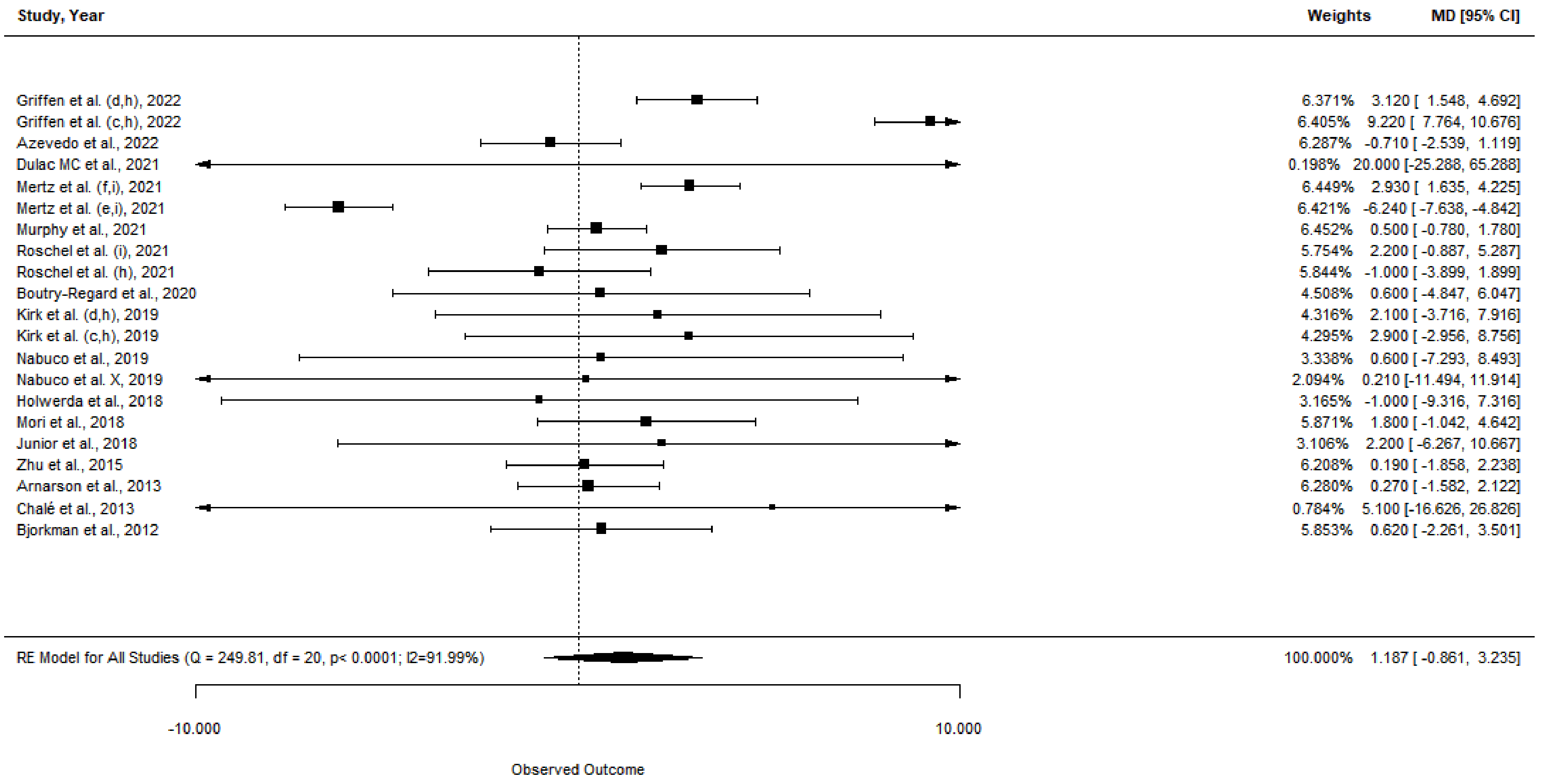

Figure S12. Forest plot of the Randomized Clinical Trials (RCTs) examining the effect of whey protein supplementation on lower body strength. Data have been expressed as MDs between intervention and control groups with 95% CI. Estimates were pooled using the random- effects. Letters between parentheses represent: c: with exercise training, d: without exercise training, d: without exercise training, e: control=CHO, f: control= collagen, h= Leg Press, i= Knee Extension. Abbreviations: MD= Mean Difference, CI= Confidence Interval.

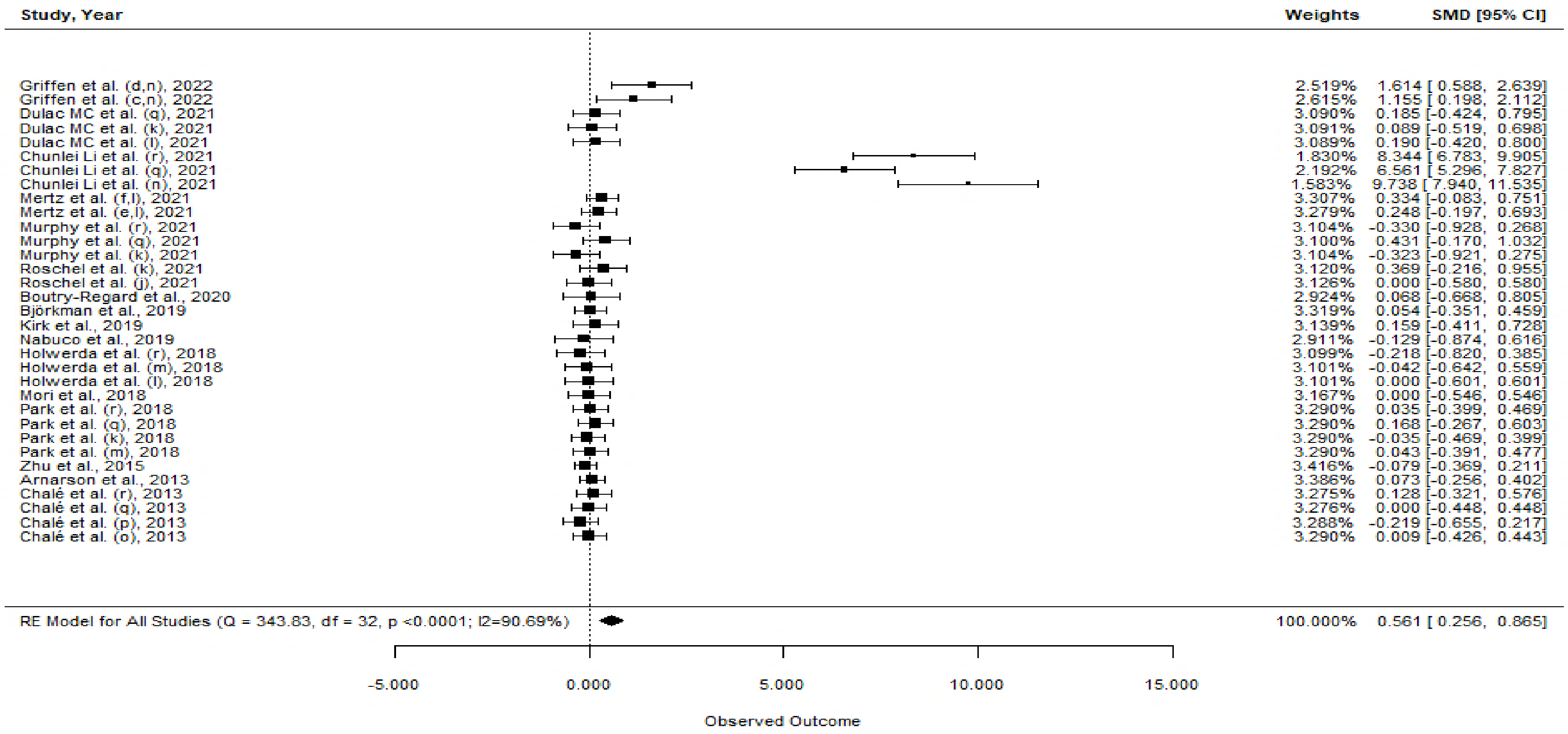

Figure S13. Forest plot of the Randomized Clinical Trials (RCTs) examining the effect of whey protein supplementation on physical function. Data have been expressed as SMDs between intervention and control groups with 95% CI. Estimates were pooled using the random- effects, Hedges model. Letters between parentheses represent: c: with exercise training, d: without exercise training, e: control=CHO, f: control= collagen, j: timed stand, k: TUG, l: walking time(s), m: sit to stand, n: chair stand, o: chair rise, p: stair climb, q: GS, r: SPPB. Abbreviations: SMD= Standard Mean Difference, CI= Confidence Interval, TUG= Timed up and go, GS= Gait speed, SPPB: Short physical performance battery.

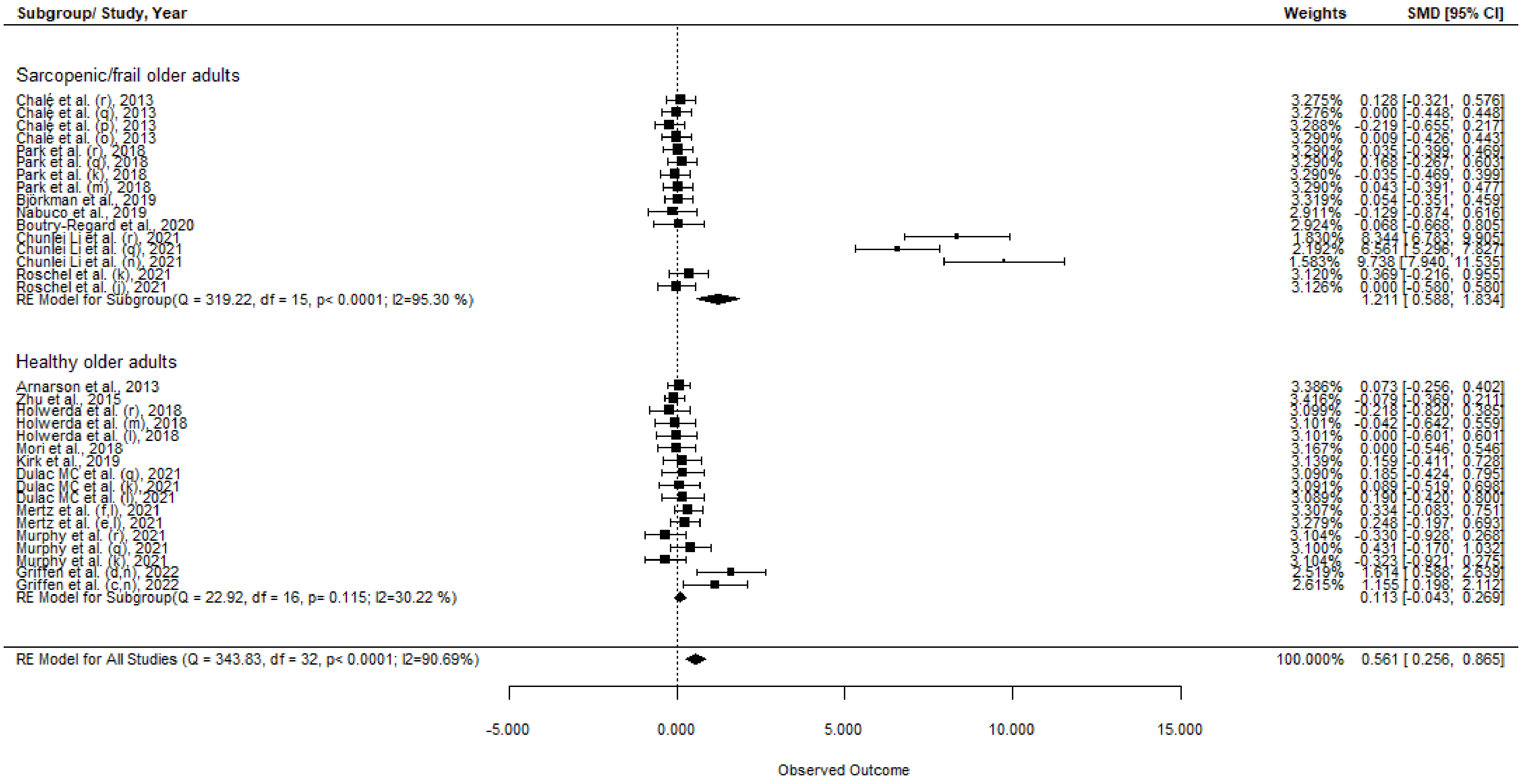

Figure S14. Forest plot of the Randomized Clinical Trials (RCT) examining the effect of whey protein supplementation on Physical Function (subgrouping sarcopenic/frail and healthy older adults). Data are expressed as SMDs between intervention and control groups with 95% CI. Estimates were pooled using the random-effects Hedges model. Letters between parentheses represent: c: with exercise training, d: without exercise training, e: control = CHO, f: control = collagen, j: timed stand, k: TUG, l: walking time(s), m: sit to stand, n: chair stand, o: chair rise, p: stair climb, q: GS, r: SPPB. Abbreviations: SMD= Standard Mean Difference, CI= Confidence Interval, TUG= Timed up and go, GS= Gait speed, SPPB: Short physical performance battery.

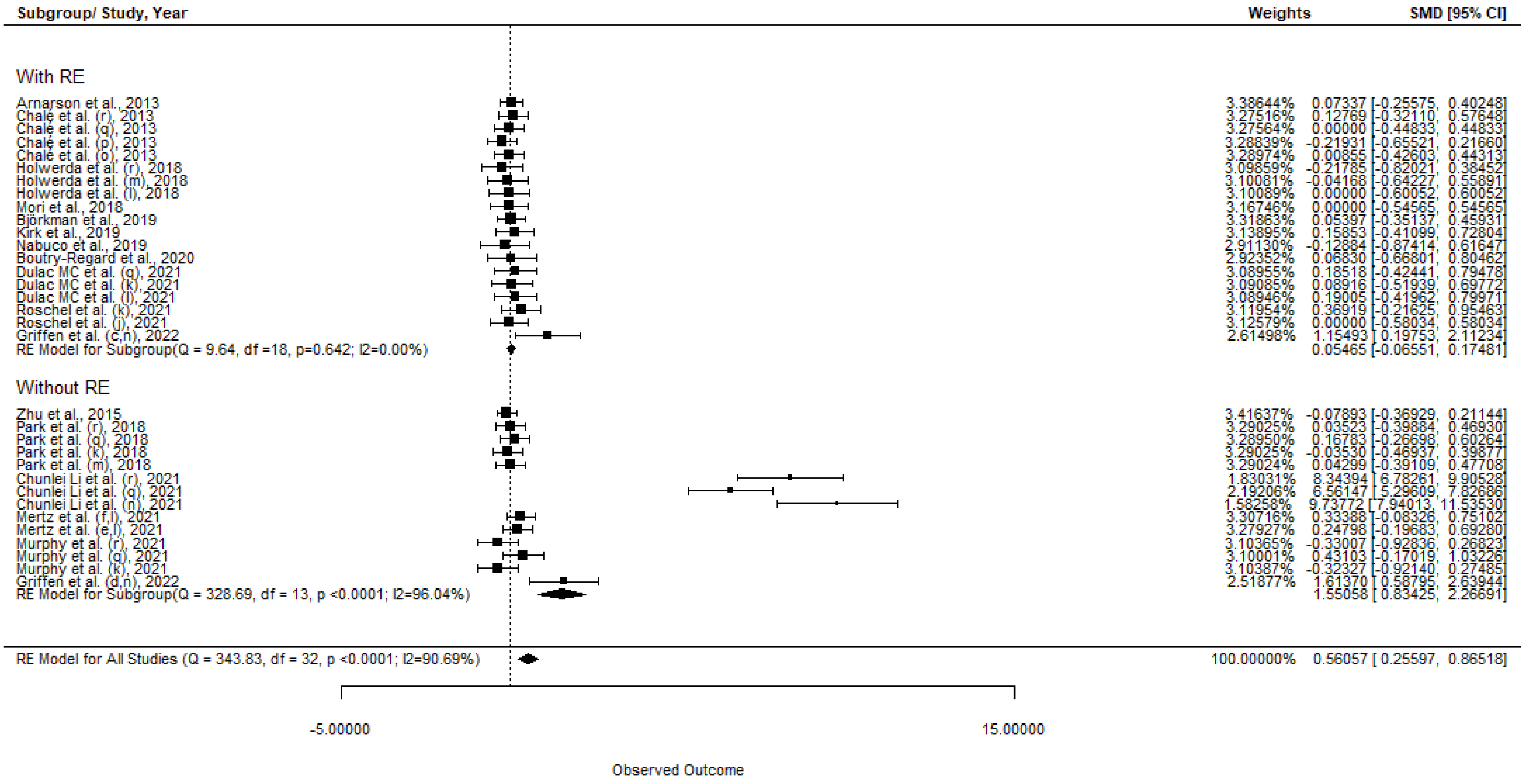

Figure S15. Forest plot of the Randomized Clinical Trials (RCTs) examining the effect of whey protein supplementation on physical function (subgrouping resistance exercise (RE)). Data have been expressed as SMDs between intervention and control groups with 95% CI. Estimates were pooled using the random- effects, Hedges model. Letters between parentheses represent: c: with exercise training, d: without exercise training, e: control=CHO, f: control= collagen, j: timed stand, k: TUG, l: walking time(s), m: sit to stand, n: chair stand, o: chair rise, p: stair climb, q: GS, r: SPPB. Abbreviations: SMD= Standard Mean Difference, CI= Confidence Interval, TUG= Timed up and go, GS= Gait speed, SPPB: Short physical performance battery.

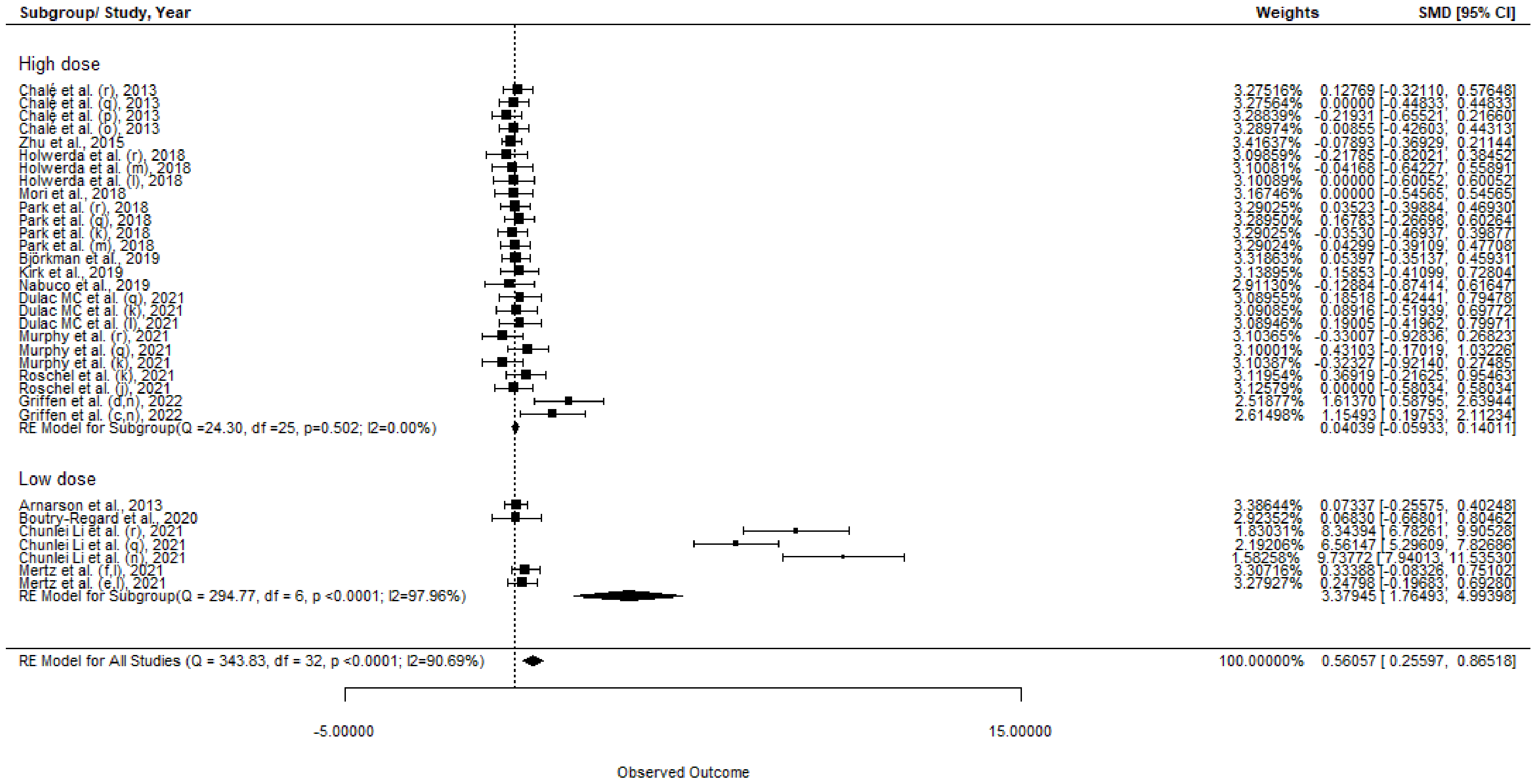

Figure S16. Forest plot of the Randomized Clinical Trials (RCTs) examining the effect of whey protein supplementation on physical function (subgrouping dose of whey). Data have been expressed as SMDs between intervention and control groups with 95% CI. Estimates were pooled using the random- effects, Hedges model. Letters between parentheses represent: c: with exercise training, d: without exercise training, e: control=CHO, f: control= collagen, j: timed stand, k: TUG, l: walking time(s), m: sit to stand, n: chair stand, o: chair rise, p: stair climb, q: GS, r: SPPB. Abbreviations: SMD= Standard Mean Difference, CI= Confidence Interval, TUG= Timed up and go, GS= Gait speed, SPPB: Short physical performance battery.

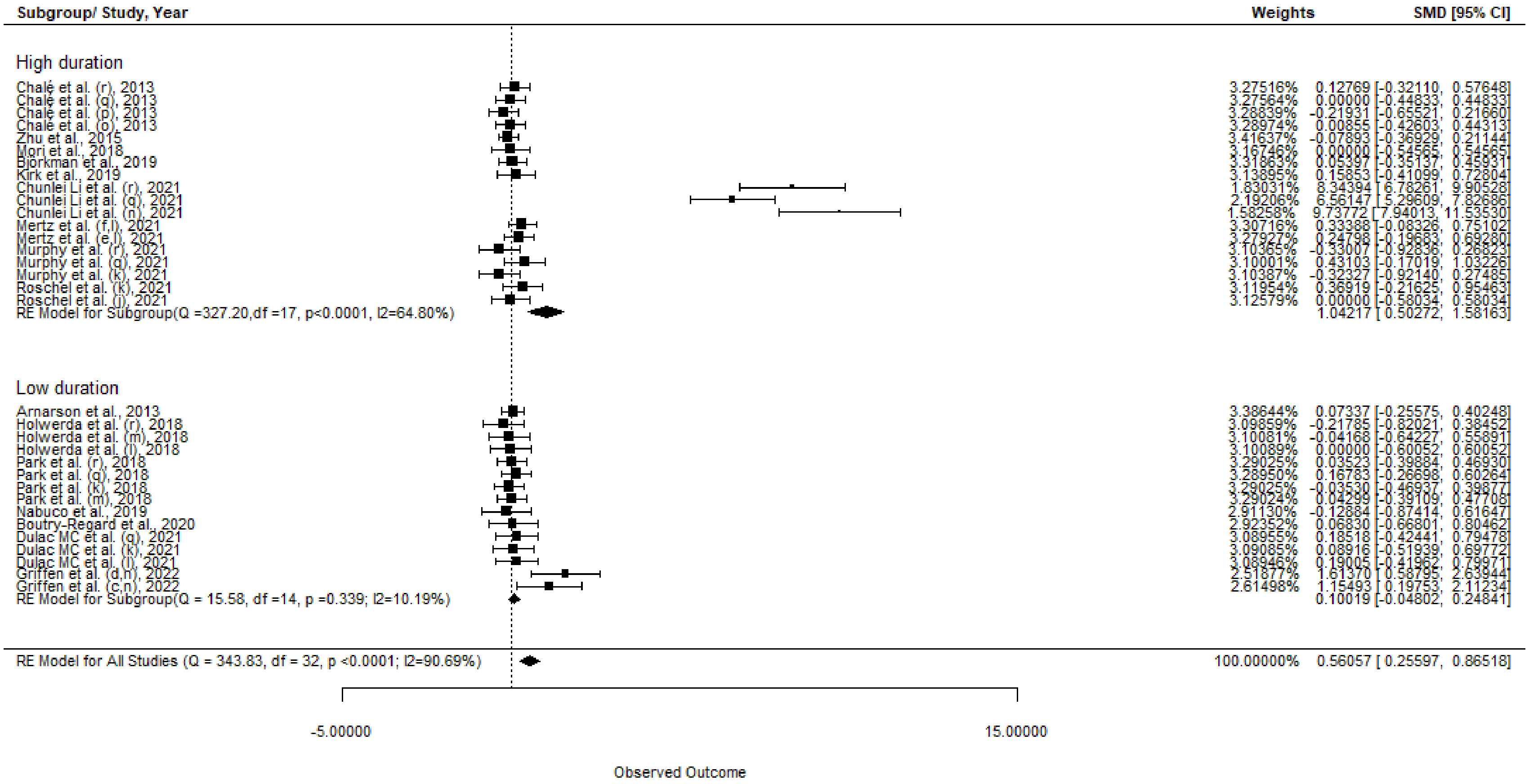

Figure S17. Forest plot of the Randomized Clinical Trials (RCTs) examining the effect of whey protein supplementation on physical function (subgrouping study duration). Data have been expressed as SMDs between intervention and control groups with 95% CI. Estimates were pooled using the random- effects, Hedges model. Letters between parentheses represent: c: with exercise training, d: without exercise training, e: control=CHO, f: control= collagen, j: timed stand, k: TUG, l: walking time(s), m: sit to stand, n: chair stand, o: chair rise, p: stair climb, q: GS, r: SPPB. Abbreviations: SMD= Standard Mean Difference, CI= Confidence Interval, TUG= Timed up and go, GS= Gait speed, SPPB: Short physical performance battery.

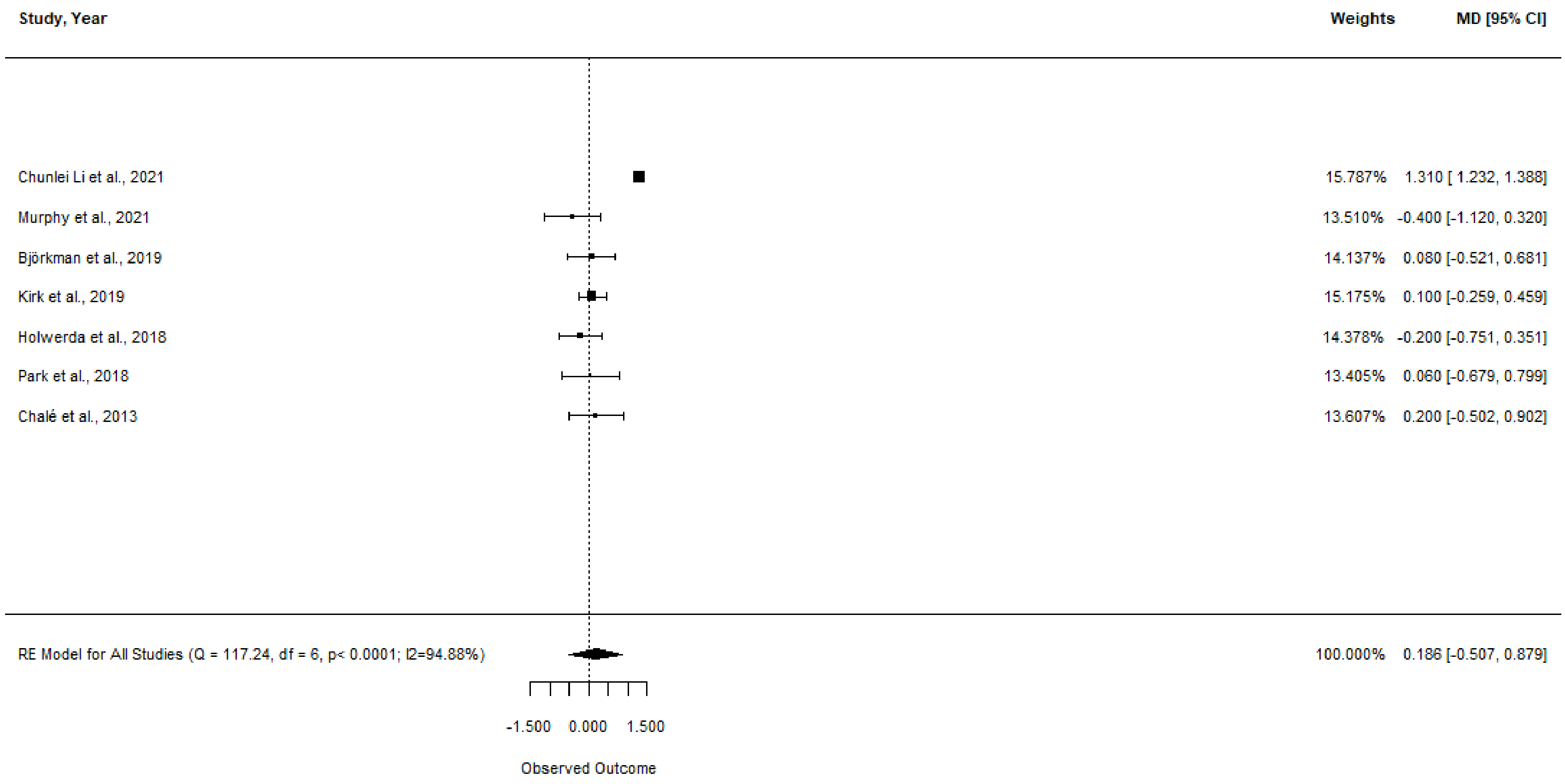

Figure S18. Forest plot of the Randomized Clinical Trials (RCTs) examining the effect of whey protein supplementation on SPPB. Data have been expressed as MDs between intervention and control groups with 95% CI. Estimates were pooled using the random- effects. Abbreviations: MD= Mean Difference, CI= Confidence Interval, SPPB: Short physical performance battery.

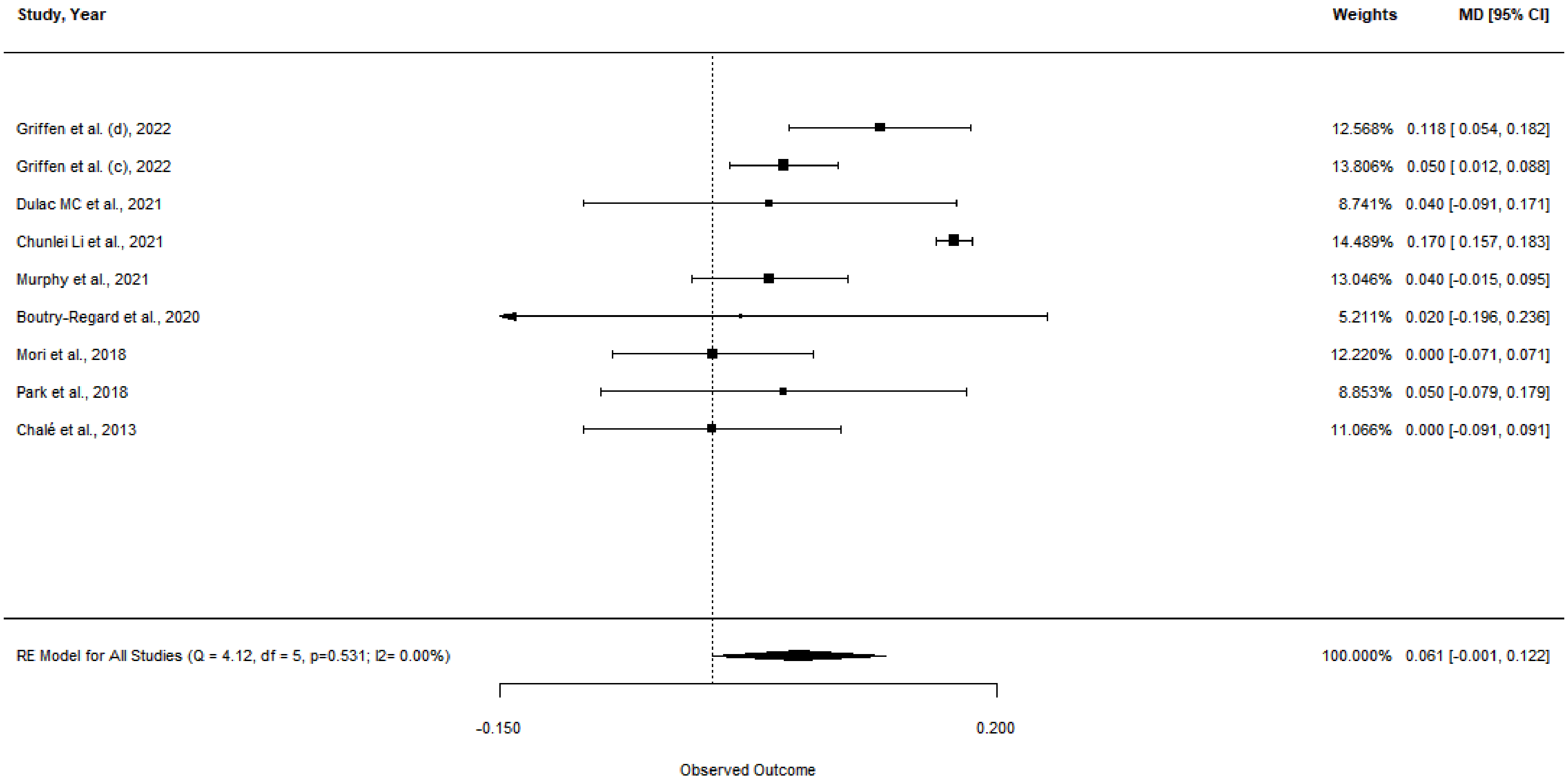

Figure S19. Forest plot of the Randomized Clinical Trials (RCTs) examining the effect of whey protein supplementation on gait speed (GS). Data have been expressed as MDs between intervention and control groups with 95% CI. Estimates were pooled using the random- effects. Letters between parentheses represent: c: with exercise training, d: without exercise training. Abbreviations: MD= Mean Difference, CI= Confidence Interval.

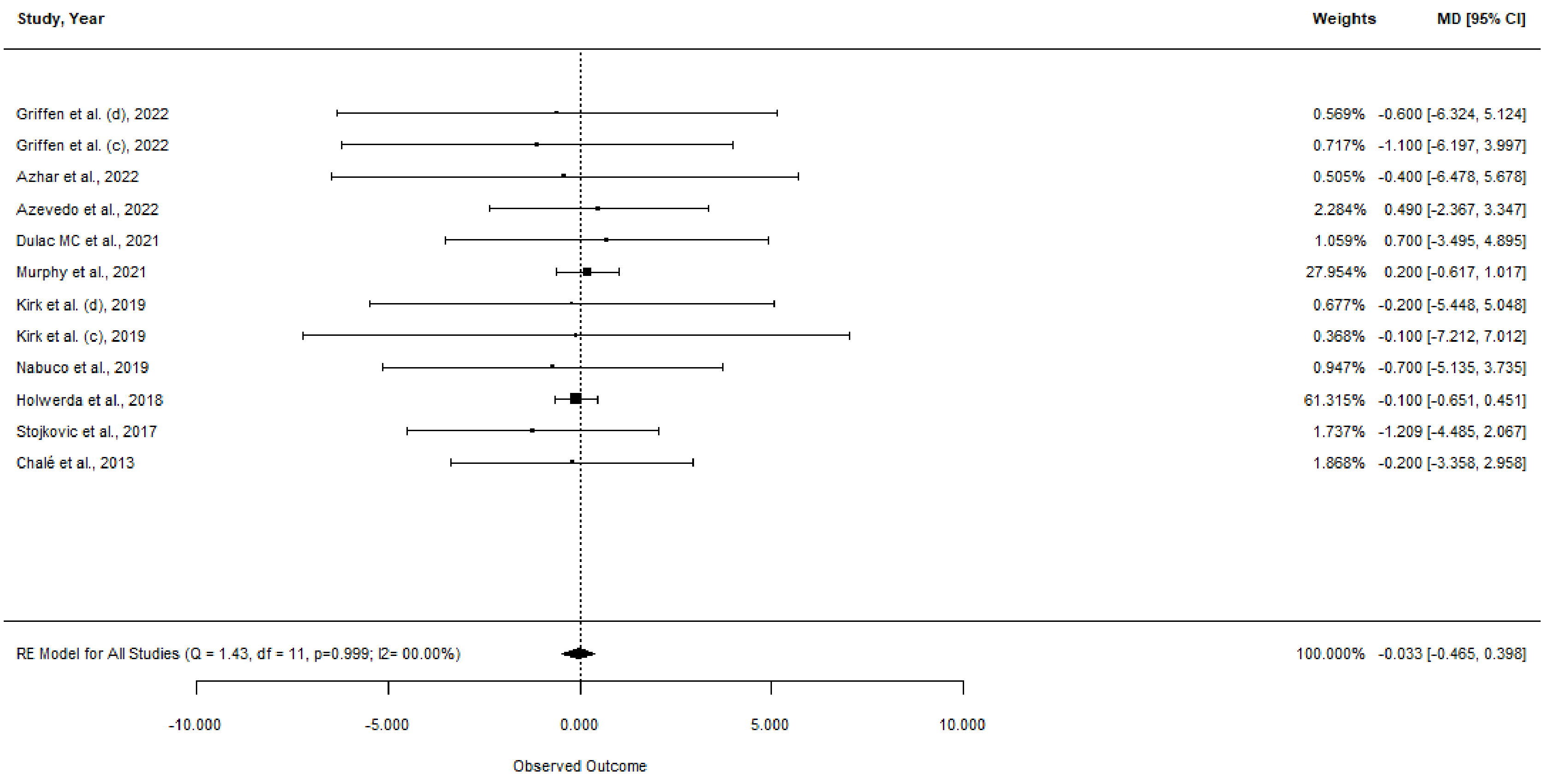

Figure S20. Forest plot of the Randomized Clinical Trials (RCTs) examining the effect of whey protein supplementation on general other physical tests. Data have been expressed as MDs between intervention and control groups with 95% CI. Estimates were pooled using the random- effects. Letters between parentheses represent: e: control=CHO, f: control= collagen, j: timed stand, k: TUG, l: walking time(s), m: sit to stand, n: chair stand, o: chair rise, p: stair climb. Abbreviations: MD= Mean Difference, CI= Confidence Interval, TUG= Timed up and go.

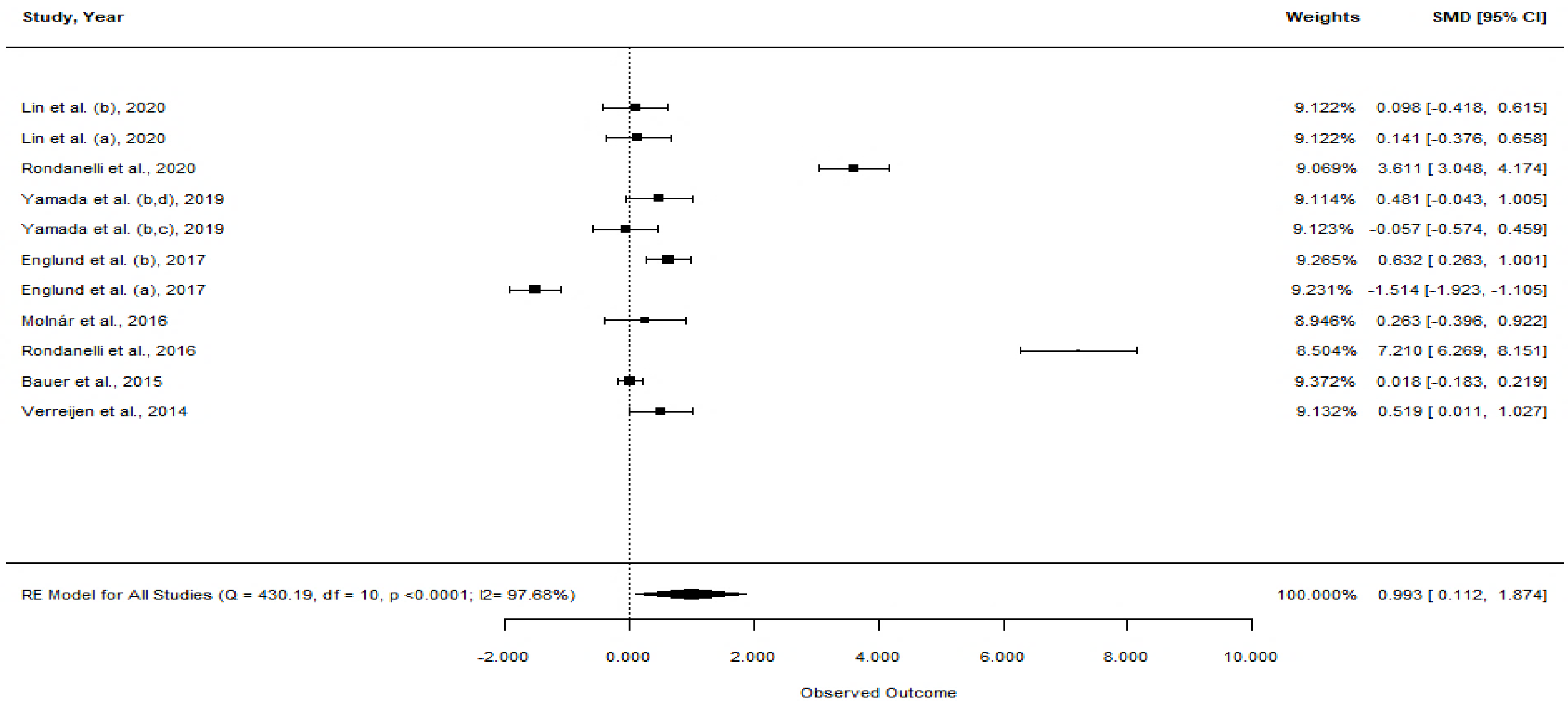

Figure S21. Forest plot of the Randomized Clinical Trials (RCTs) examining the effect of whey protein and vitamin D supplementation on LM. Data have been expressed as SMDs between intervention and control groups with 95% CI. Estimates were pooled using the random- effects, Hedges model. Letters between parentheses represent: a: TLM, b: ALM, c: with exercise training, d: without exercise training. Abbreviations: SMD= Standard Mean Difference, CI= Confidence Interval, TLM= Total Lean Mass, ALM= Appendicular Lean Mass.

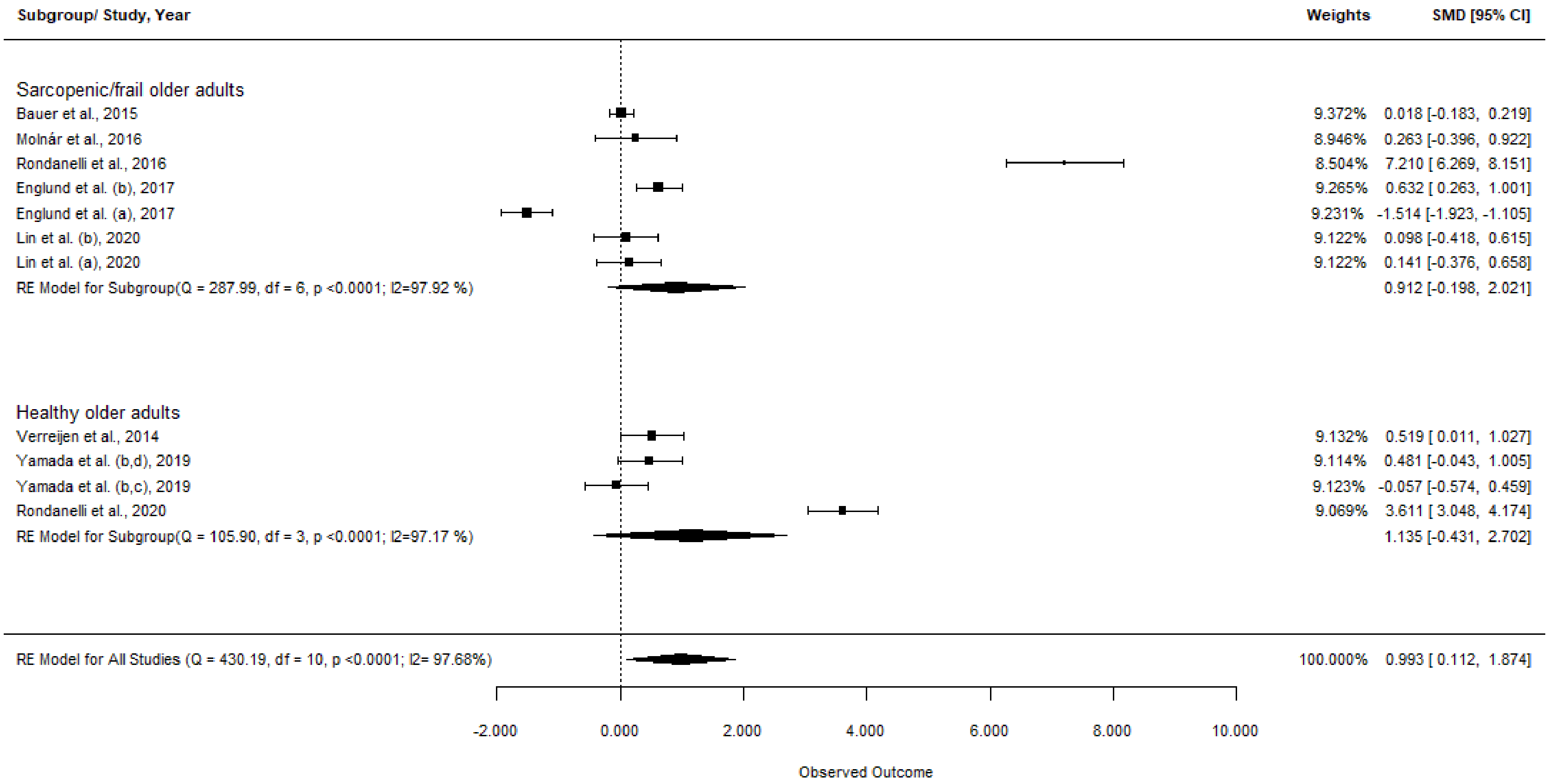

Figure S22. Forest plot of the Randomized Clinical Trials (RCTs) examining the effect of whey protein and vitamin D supplementation on LM (subgrouping sarcopenic/frail and healthy older adults). Data have been expressed as SMDs between intervention and control groups with 95% CI. Estimates were pooled using the random- effects, Hedges model. Letters between parentheses represent: a: TLM, b: ALM, c: with exercise training, d: without exercise training. Abbreviations: SMD= Standard Mean Difference, CI= Confidence Interval, TLM= Total Lean Mass, ALM= Appendicular Lean Mass.

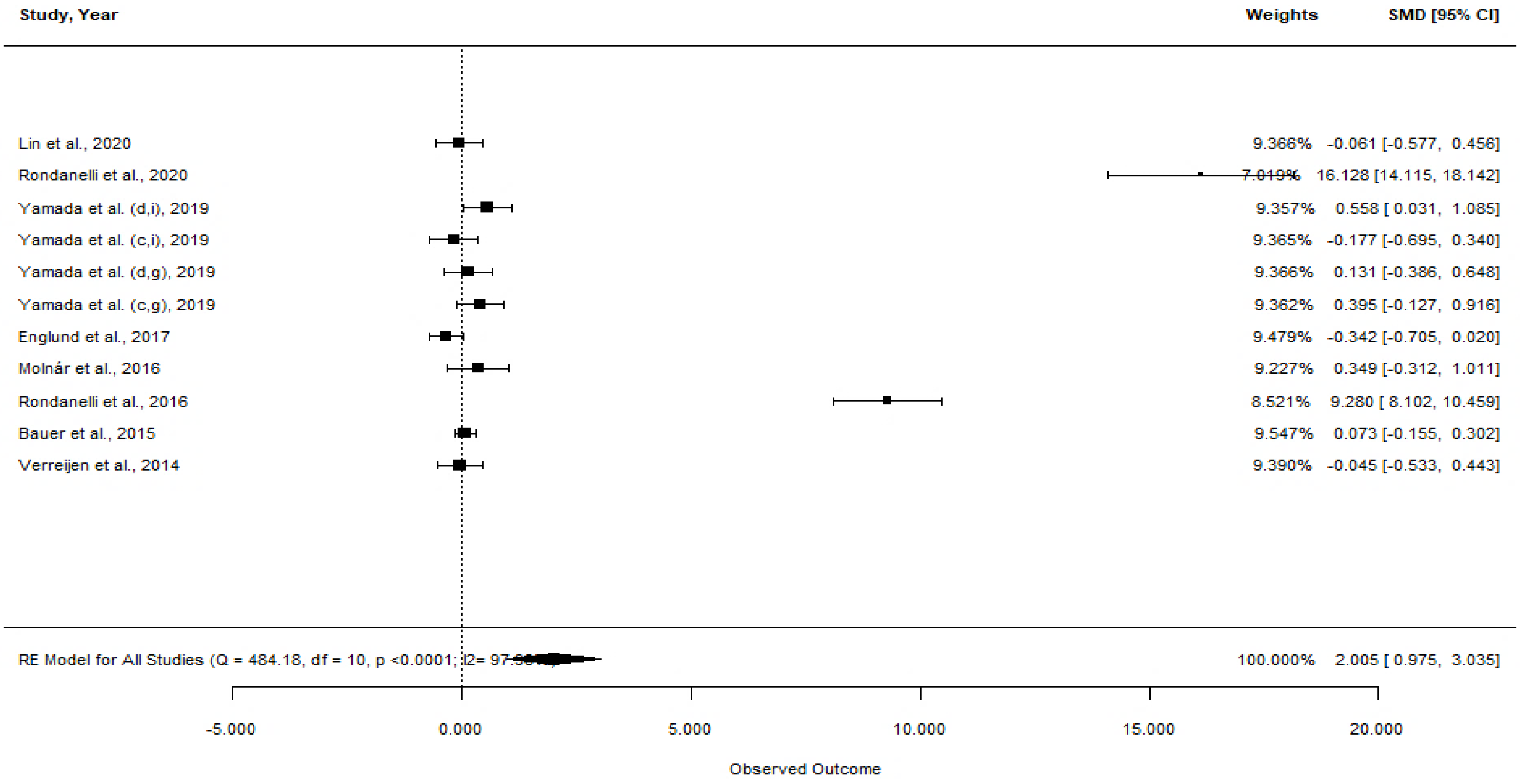

Figure S23. Forest plot of the Randomized Clinical Trials (RCTs) examining the effect of whey protein and vitamin D supplementation on muscle strength. Data have been expressed as SMDs between intervention and control groups with 95% CI. Estimates were pooled using the random- effects, Hedges model. Letters between parentheses represent: c: with exercise training, d: without exercise training, g= HGS, h= Leg Press, i= Knee Extension. Abbreviations: SMD= Standard Mean Difference, CI= Confidence Interval, HGS= handgrip strength.

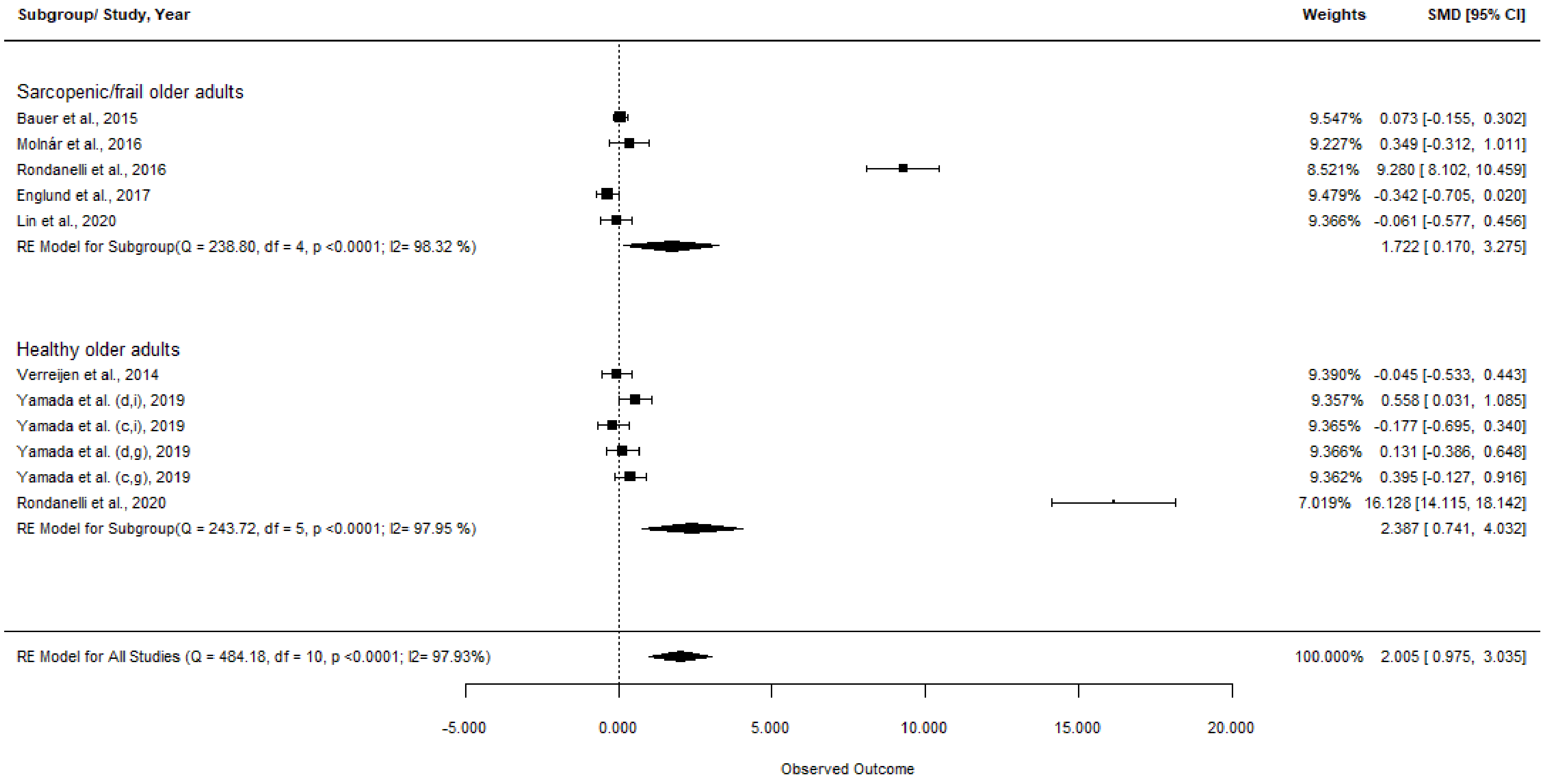

Figure S24. Forest plot of the Randomized Clinical Trials (RCTs) examining the effect of whey protein and vitamin D supplementation on muscle strength (subgrouping sarcopenic/frail and healthy older adults). Data have been expressed as SMDs between intervention and control groups with 95% CI. Estimates were pooled using the random- effects, Hedges model. Letters between parentheses represent: c: with exercise training, d: without exercise training, g= HGS, h= Leg Press, i= Knee Extension. Abbreviations: SMD= Standard Mean Difference, CI= Confidence Interval, HGS= handgrip strength.

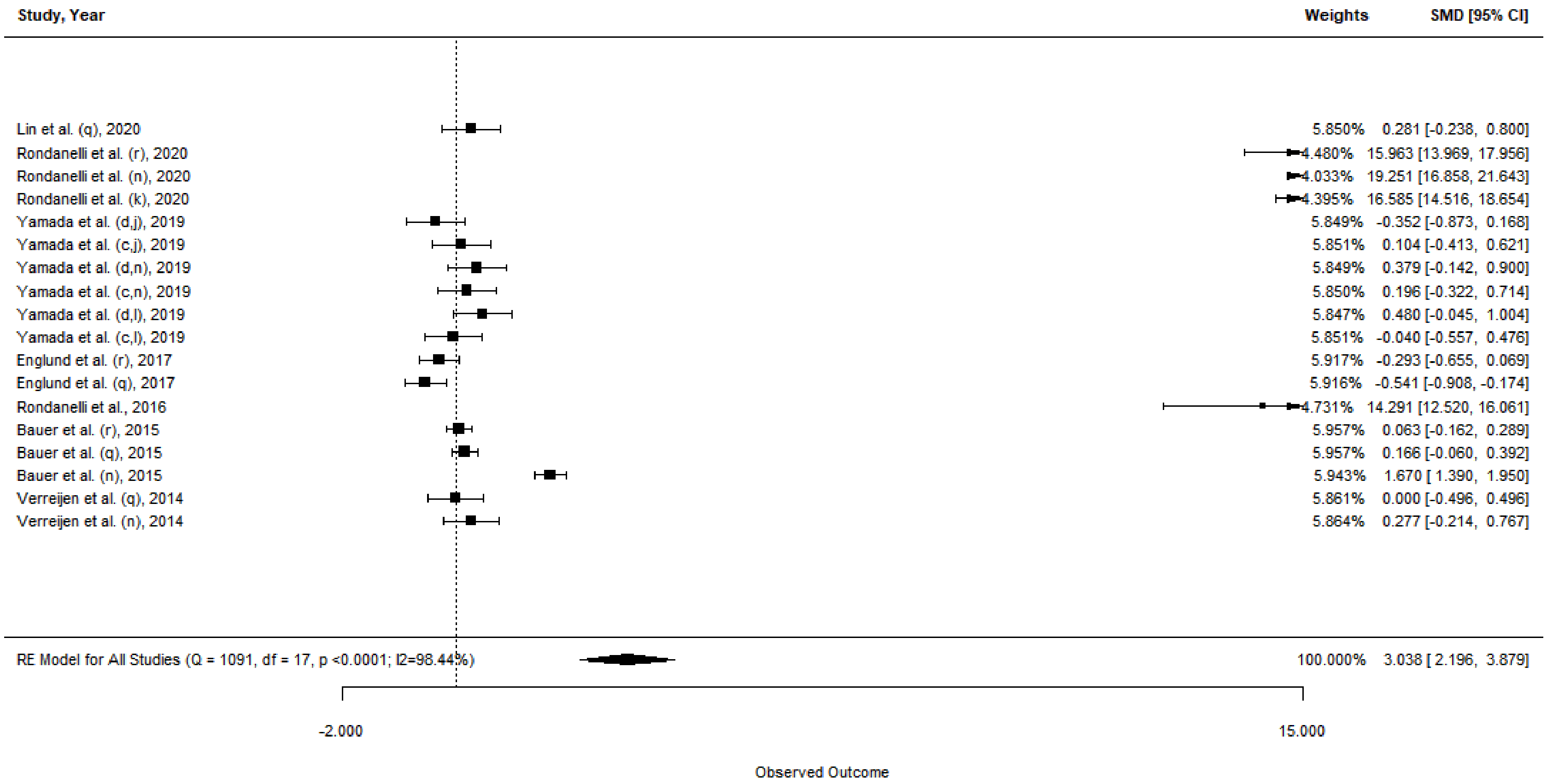

Figure S25. Forest plot of the Randomized Clinical Trials (RCTs) examining the effect of whey protein and vitamin D supplementation on physical function. Data have been expressed as SMDs between intervention and control groups with 95% CI. Estimates were pooled using the random- effects, Hedges model. Letters between parentheses represent: c: with exercise training, d: without exercise training, j: timed stand, k: TUG, l: walking time(s), m: sit to stand, n: chair stand, o: chair rise, p: stair climb, q: GS, r: SPPB. Abbreviations: SMD= Standard Mean Difference, CI= Confidence Interval, TUG= Timed up and go, GS= Gait speed, SPPB: Short physical performance battery.

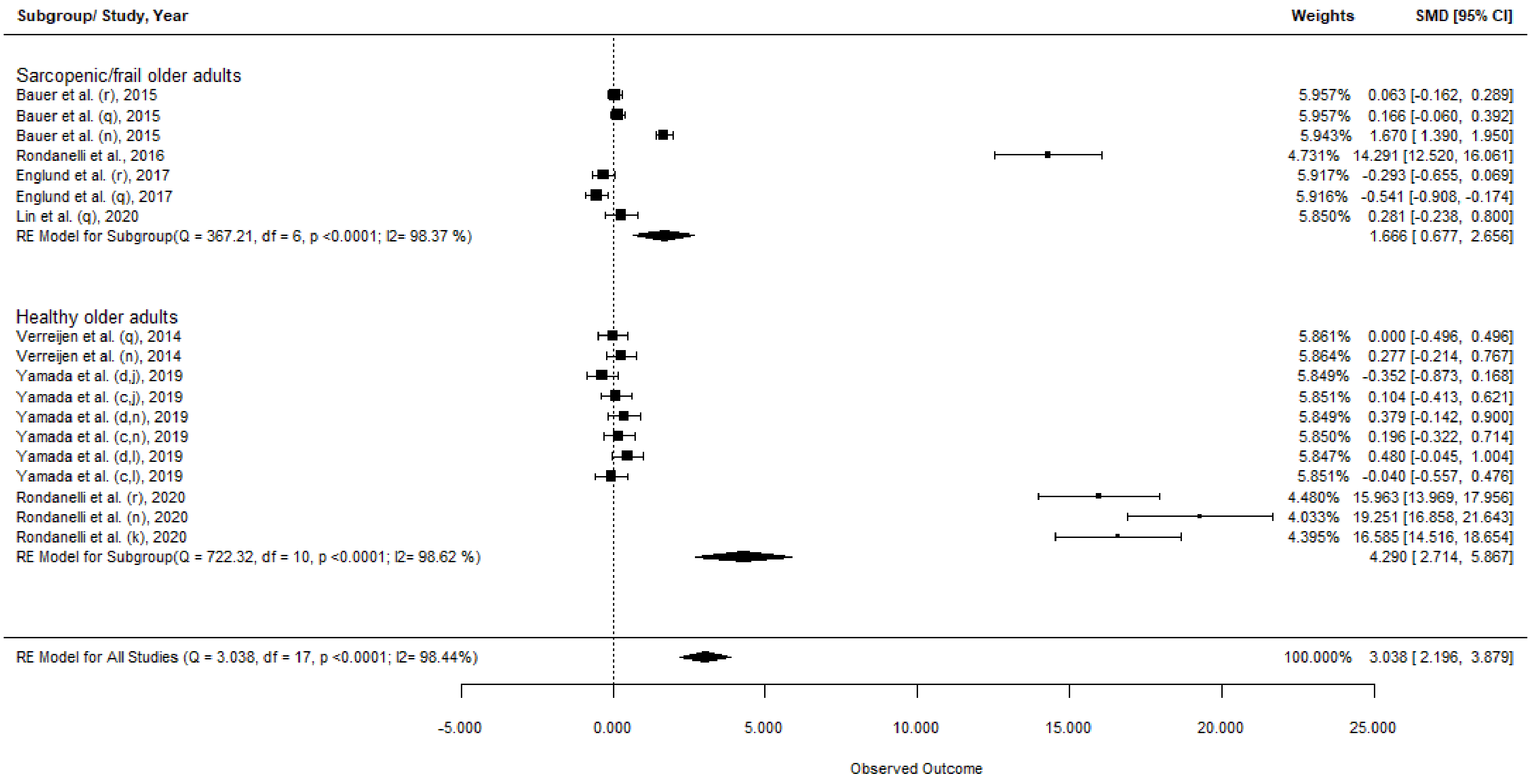

Figure S26. Forest plot of the Randomized Clinical Trials (RCTs) examining the effect of whey protein and vitamin D supplementation on physical function (subgrouping sarcopenic/frail and healthy older adults). Data have been expressed as SMDs between intervention and control groups with 95% CI. Estimates were pooled using the random- effects, Hedges model. Letters between parentheses represent: c: with exercise training, d: without exercise training, j: timed stand, k: TUG, l: walking time(s), m: sit to stand, n: chair stand, o: chair rise, p: stair climb, q: GS, r: SPPB. Abbreviations: SMD= Standard Mean Difference, CI= Confidence Interval, TUG= Timed up and go, GS= Gait speed, SPPB: Short physical performance battery.

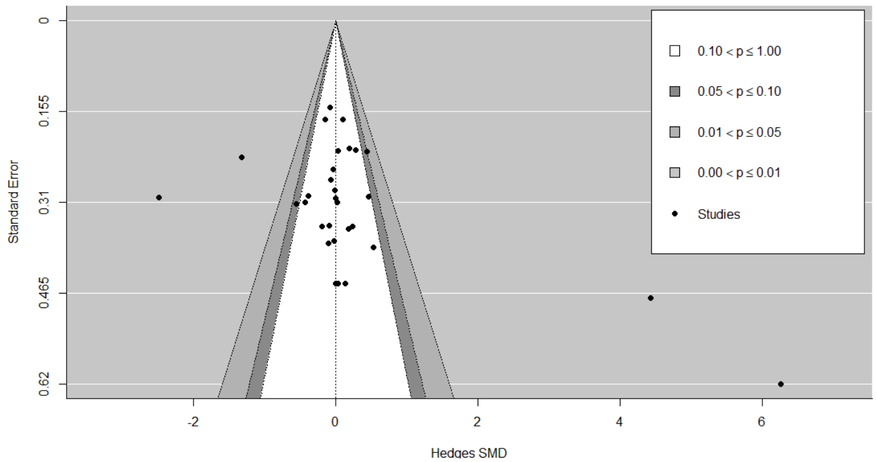

Figure S27 Funnel plot showing results all studies testing the effects of whey protein supplementation on LM.

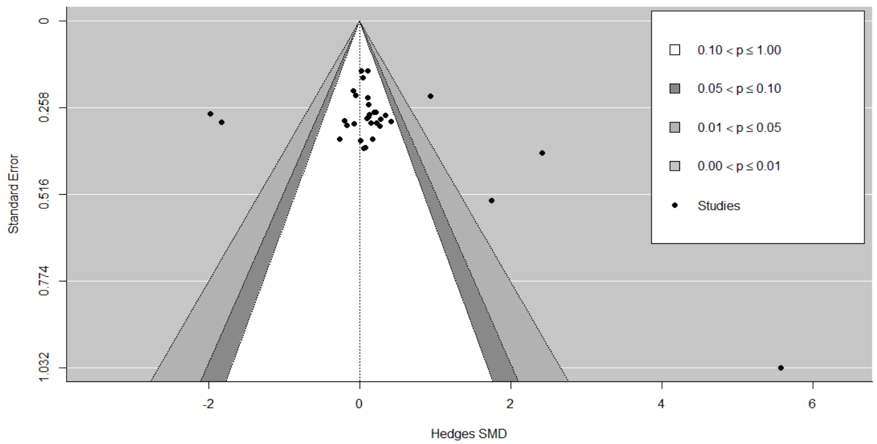

Figure S28 Funnel plot showing results all studies testing the effects of whey protein supplementation on muscle strength.

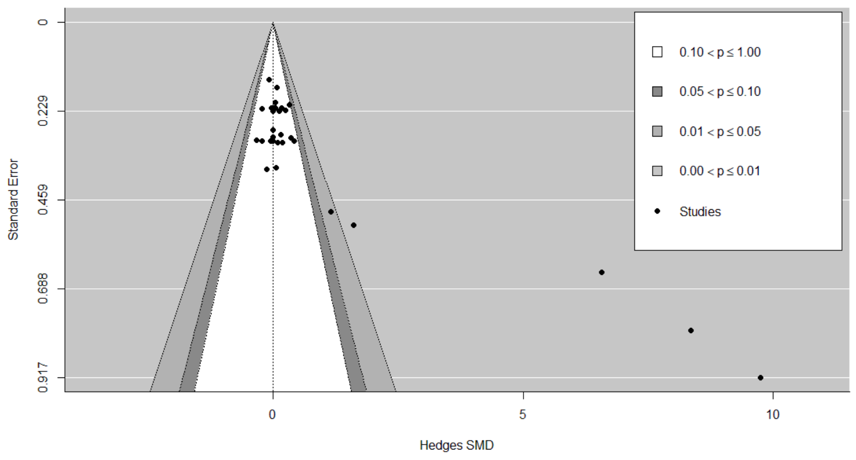

Figure S29 Funnel plot showing results all studies testing the effects of whey protein supplementation on muscle function.

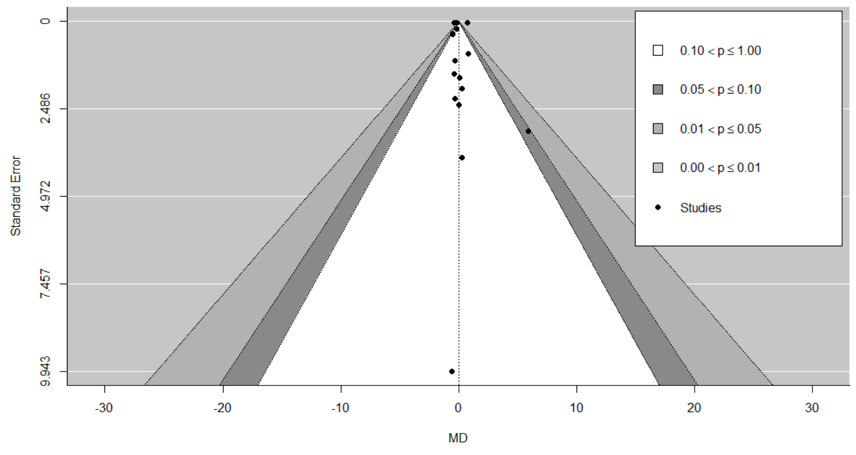

Figure S30 Funnel plot showing results all studies testing the effects of whey protein supplementation on TLM.

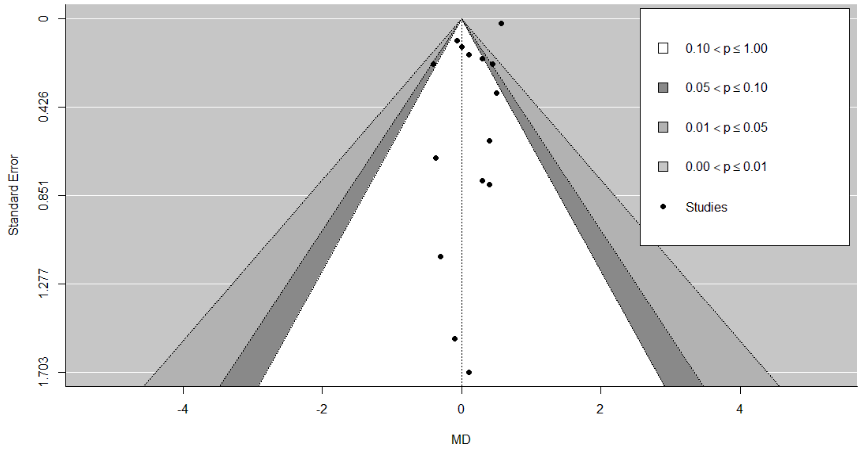

Figure S31 Funnel plot showing results all studies testing the effects of whey protein supplementation on ALM.

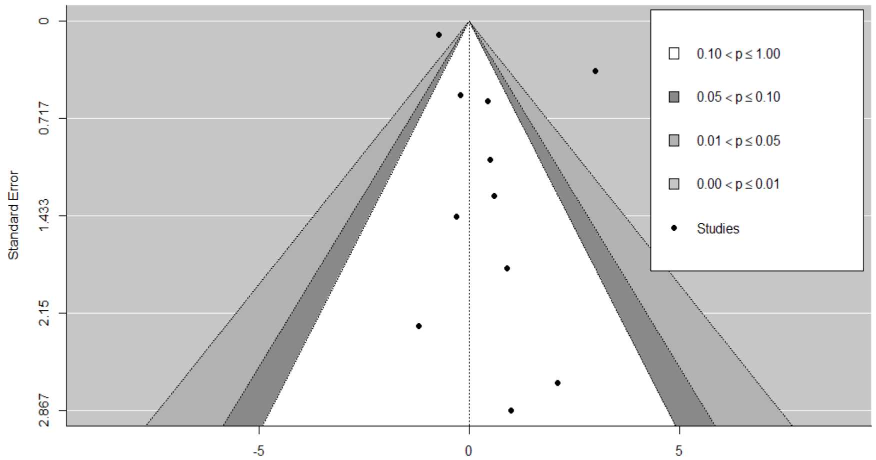

Figure S32 Funnel plot showing results all studies testing the effects of whey protein supplementation on handgrip strength.

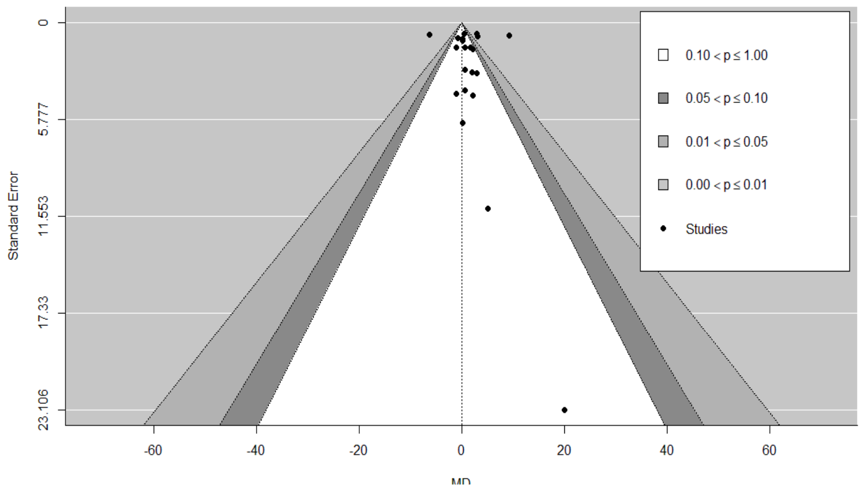

Figure S33 Funnel plot showing results all studies testing the effects of whey protein supplementation on lower body strength.

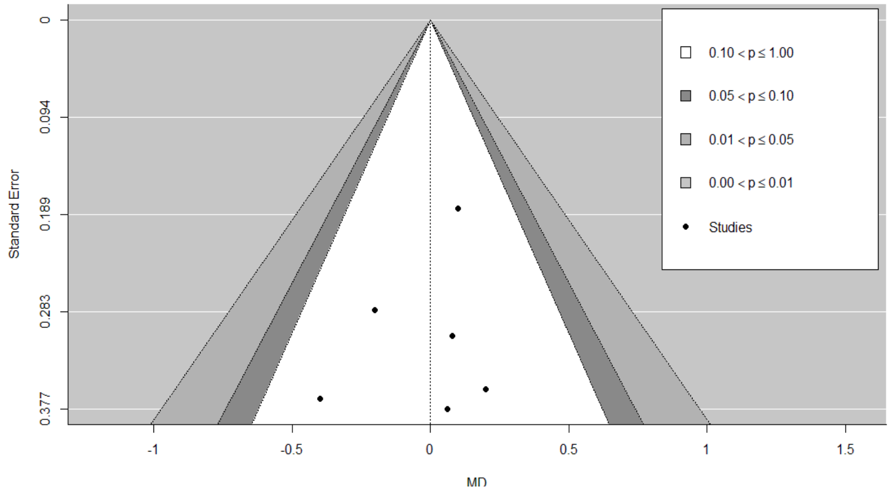

Figure S34 Funnel plot showing results all studies testing the effects of whey protein supplementation on SPPB.

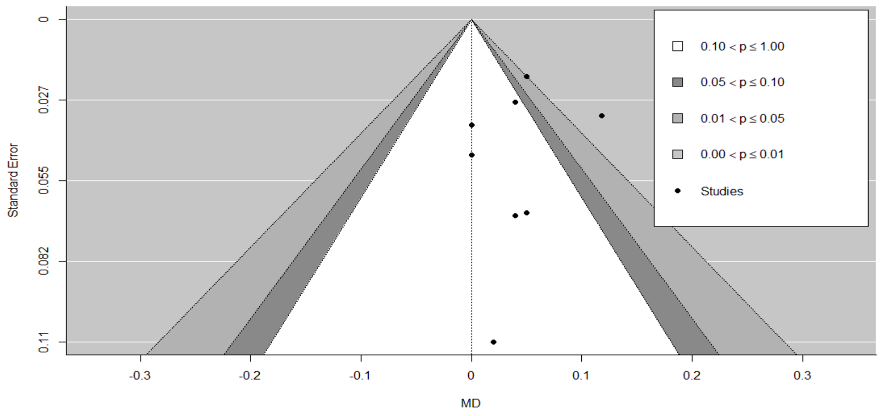

Figure S35 Funnel plot showing results all studies testing the effects of whey protein supplementation on GS.

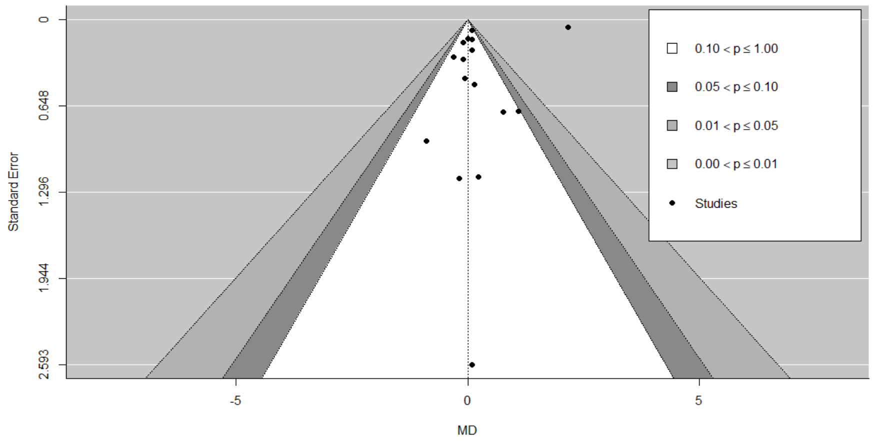

Figure S36 Funnel plot showing results all studies testing the effects of whey protein supplementation on other physical tests.

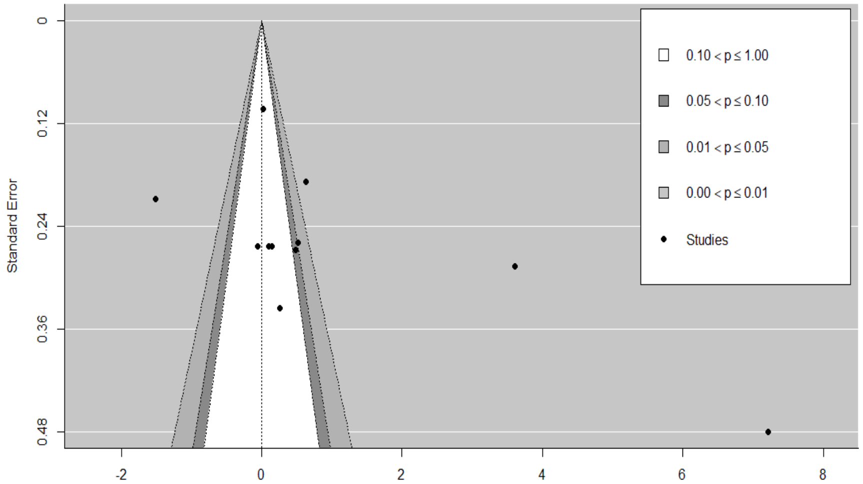

Figure S37 Funnel plot showing results all studies testing the effects of whey protein and vitamin D supplementation on LM.

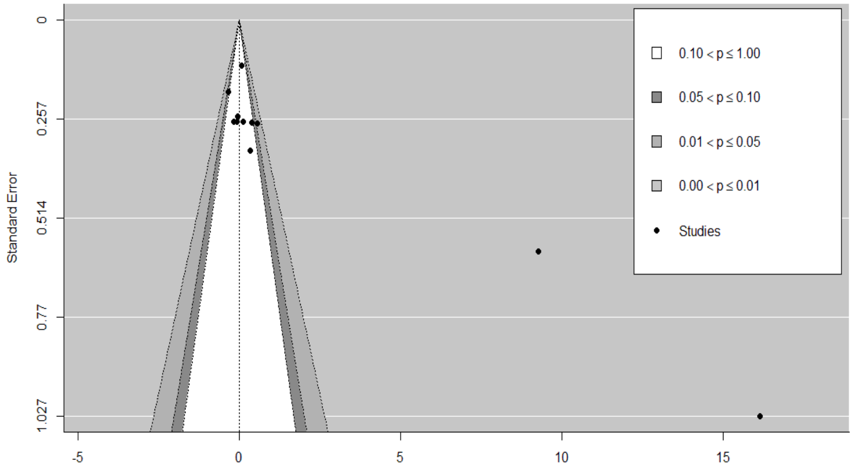

Figure S38 Funnel plot showing results all studies testing the effects of whey protein and vitamin D supplementation on muscle strength.

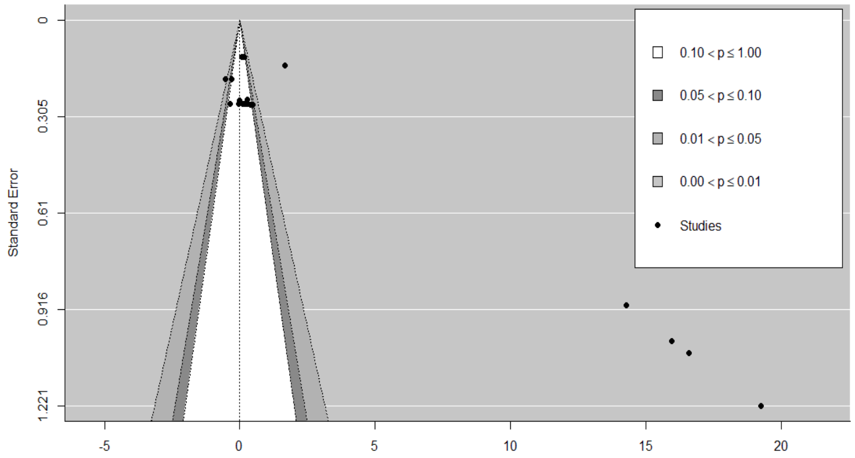

Figure S39 Funnel plot showing results all studies testing the effects of whey protein and vitamin D supplementation on muscle function.
